# Rational Design of Novel Thiazole-Clubbed Pyrimidine-Linked Hydrazone Conjugates as Promising RSK4 Inhibitors for Esophageal Squamous Cell Carcinoma: Molecular Dynamics Simulations and In Vitro Evaluation

**DOI:** 10.3390/ph19081323

**Published:** 2026-08-21

**Authors:** Mujeeb Ul Naeem, Syeda Farwa Naqvi, Yousaf Khan, Samina Aslam, Syed Aminullah, Azmatullah Khan, Thoraya A. Farghaly, Wajid Rehman

**Affiliations:** 1 Department of Chemistry, Hazara University, Mansehra 21120, Pakistan; mujeebulnaeem@gmail.com (M.U.N.); sono_waj@yahoo.com (W.R.); 2 Department of Zoology, Quaid-i-Azam University Islamabad, Islamabad 45320, Pakistan; farwa.naqvi@bs.qau.edu.pk (S.F.N.); azmat6520@gmail.com (A.K.); 3 Department of Chemistry, COMSATS University Islamabad, Islamabad 45550, Pakistan; syedaminlec999@gmail.com; 4 Department of Chemistry, Sardar Bahadur Khan Women University Quetta, Quetta 87300, Pakistan; saminaaslampanizai@yahoo.com; 5 Chemistry Department, Faculty of Science, Umm Al-Qura University, Makkah 21955, Saudi Arabia; thoraya-f@hotmail.com

**Keywords:** thiazole, pyrimidine, hydrazone, RSK4, ADMET, molecular docking and dynamic simulations, cytotoxicity

## Abstract

**Background:** Esophageal squamous cell carcinoma (ESCC) remains highly aggressive and continues to limit clinical treatment for substantial cancer-associated morbidity and mortality worldwide. Despite advances in therapeutic interventions the lack of effective molecularly targeted treatments continues to restrict clinical management beyond conventional chemotherapy and radiotherapy. Among these therapeutic targets the ribosomal S6 kinase 4 (RSK4) has gained considerable attention because of its critical involvement in ESCC progression, survival and proliferation, suggesting its potential as a potential target for anticancer drug development. **Methods:** A series of thiazole-clubbed pyrimidine linked hydrazone hybrids (**1**–**14**) were synthesized via 4-aminothiazole-5-carbohydrazide functionalized intermediates and fully characterized and evaluated for their inhibitory activity against RSK4. **Results:** Biological assessment demonstrated that the synthesized analogues exhibited remarkable potency, with IC_50_ values between 15.32 ± 1.35 and 54.61 ± 2.17 nM compared with the reference inhibitor BI-D1870 (IC_50_ = 33.16 ± 1.34 nM). Among the evaluated compounds, **2**, **8**, **9**, **13** and **14** emerged as the most potent candidates and showed pronounced activity towards RSK4. For further insights into the molecular basis of their activity the lead candidates were subjected to computational investigations, such as molecular docking, molecular dynamics simulations, in silico ADMET and ProTox-3.0 characterization. The computational analyses provided structural and pharmacological insights into experimentally observed RSK4 inhibitory activity, like predicted interactions, stability dynamically and preliminary ADMET/toxicity characteristics. This study supports the need for further optimization and experimental validation of the identified RSK4 active lead candidates. **Conclusions:** These findings highlight the thiazole-clubbed pyrimidine-linked hydrazone scaffold as a potential chemotype for promising scaffolds targeting RSK4 lead discovery, and the identified candidates warrant further investigation into ESCC cellular models to establish anticancer efficacy and pathway-level activity.

## 1. Introduction

Esophageal cancer (ESCA) constitutes a significant global health burden owing to its high incidence and mortality. According to the Global Cancer Statistics 2022 (GLOBOCAN), an estimated 511,000 new cases and 445,000 deaths were recorded worldwide, which ranks ESCA as the 11th most frequently diagnosed cancer and the seventh leading cause of cancer-related mortality. These alarming statistics highlight the urgent need for the development of more effective therapeutic strategies [1,2]. Despite considerable progress in diagnostic and therapeutic approaches the clinical outcome of ESCC remains highly unfavorable, with the overall five-year survival rate being below 20% [3]. Current treatment modalities, e.g., chemotherapy, surgical intervention, radiotherapy and chemoradiotherapy, provide only modest therapeutic benefit because of frequent disease recurrence, metastasis progression and limited availability of effective molecularly targeted therapies. The identification of novel therapeutic targets and the development of more promising anticancer agents remain critical priorities to improve ESCC management [4,5].

Recent studies have identified cancer stem cell-like cells (CSCs) as key regulators of ESCC development and progression. Their biological functions are strongly associated with tumorigenesis, metastatic progression, resistance to conventional therapies and post-treatment relapse [6,7]. Resistance to radiotherapy has been closely linked to CSC-associated signaling pathways. Previous mechanistic investigations have demonstrated that ribosomal S6 kinase 4 (RSK4) is markedly over expressed in ESCC-derived CSC populations and plays a central role to maintain stemness properties through modulation of the ΔNp63α-RSK4-GSK4-3β signaling axis [8,9,10]. Elevated RSK4 expression has been associated with enhanced radio-resistance and reduced patient survival, highlighting RSK4 as a potent molecular target for therapeutic intervention in ESCC [11,12].

RSK4 is a member of the p90 ribosomal S6 kinase (RSK) family of serine/threonine protein kinases, which comprises four highly homologous isoforms, namely RSK1, RSK2, RSK3 and RSK4 [13,14]. Members of this kinase family regulate a broad spectrum of cellular functions, such as migration, proliferation, survival, cell-cycle progression and differentiation [15]. RSK proteins contain two catalytically functional kinase domains, such as the C-terminal kinase domain (CTKD) and N-terminal kinase domain (NTKD). Canonical activation is initiated through Ras/ERK-mediated phosphorylation of the CTKD, which subsequently promotes autophosphorylation and activation of the NTKD and leads to phosphorylation of downstream effector proteins [13,16,17]. RSK4 exhibits a distinctive regulatory mechanism compared with other RSK isoforms and appears to retain activity independent of classical growth-factor-driven MAPK signaling pathways [18,19]. Although the precise physiological and pathological functions of RSK4 have not yet been fully elucidated emerging studies suggest its involvement in the regulation of apoptosis, p21-mediated pathways, p53-dependent signaling and cellular metabolism [20,21].

Aberrant RSK4 expression has been implicated in a variety of pathological conditions, such as breast, lung, renal and esophageal malignancies as well as metabolic disorders, such as diabetes mellitus. RSK4 displays context-dependent biological functions and may act either as a tumor suppressor or an oncogenic mediator, depending on the cellular context and tumor microenvironment. The substantial experimental evidence strongly supports its oncogenic role, particularly in facilitating therapeutic resistance and aggressive tumor phenotypes. These observations underscore the therapeutic potential of RSK4 and highlight the need for the development of potent and potential RSK4 inhibitors for targeted ESCC treatment [22,23,24,25].

**Figure 1:**
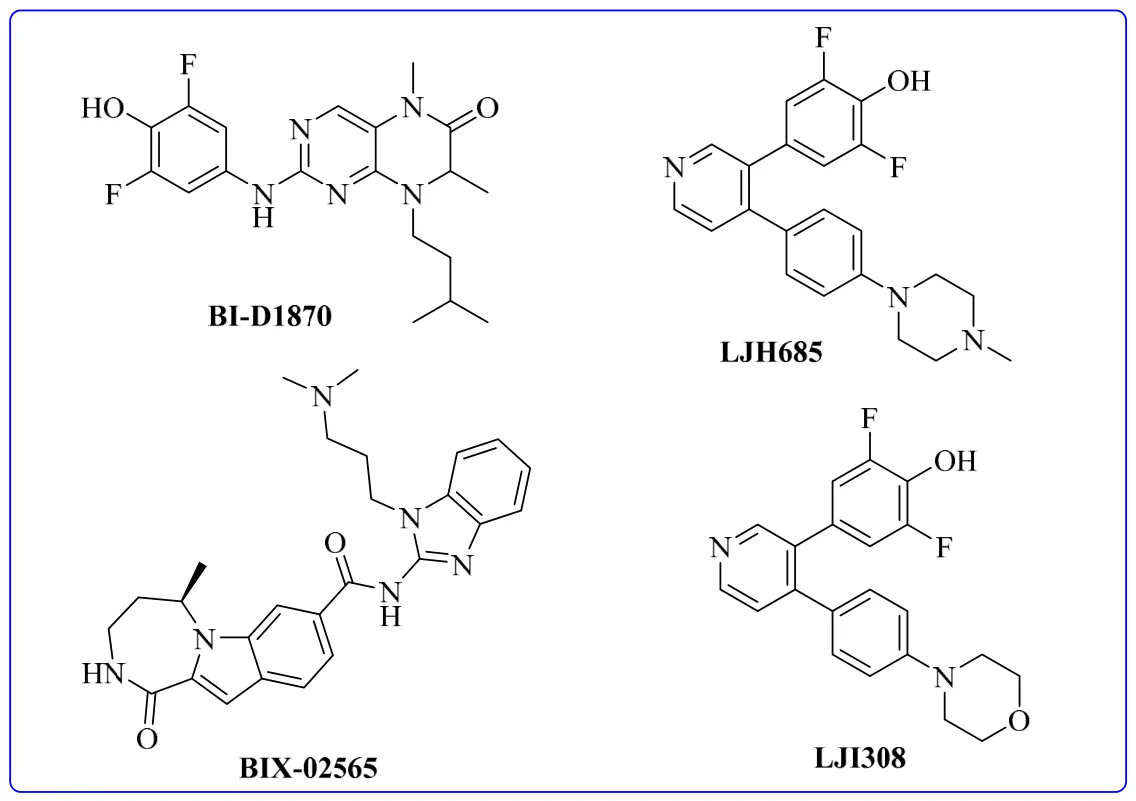
Structurally unique, highly potent small-molecule inhibitors of ribosomal S6 kinases (RSKs).

Only a limited number of RSK inhibitors have been reported, and none have received approval from the U.S. Food and Drug Administration (FDA). Early-generation inhibitors, such as SL0101, have demonstrated inhibitory activity against the NTKD of RSK family members [26,27]. Several ATP-competitive inhibitors, e.g., BI-D1870, BIX-02565, LJH685 and LJI308, were developed and exhibited potent nanomolar inhibitory activities against various RSK isoforms [28]. The chemical structures of these representative RSK inhibitors are presented in Figure 1.

**Figure 2:**
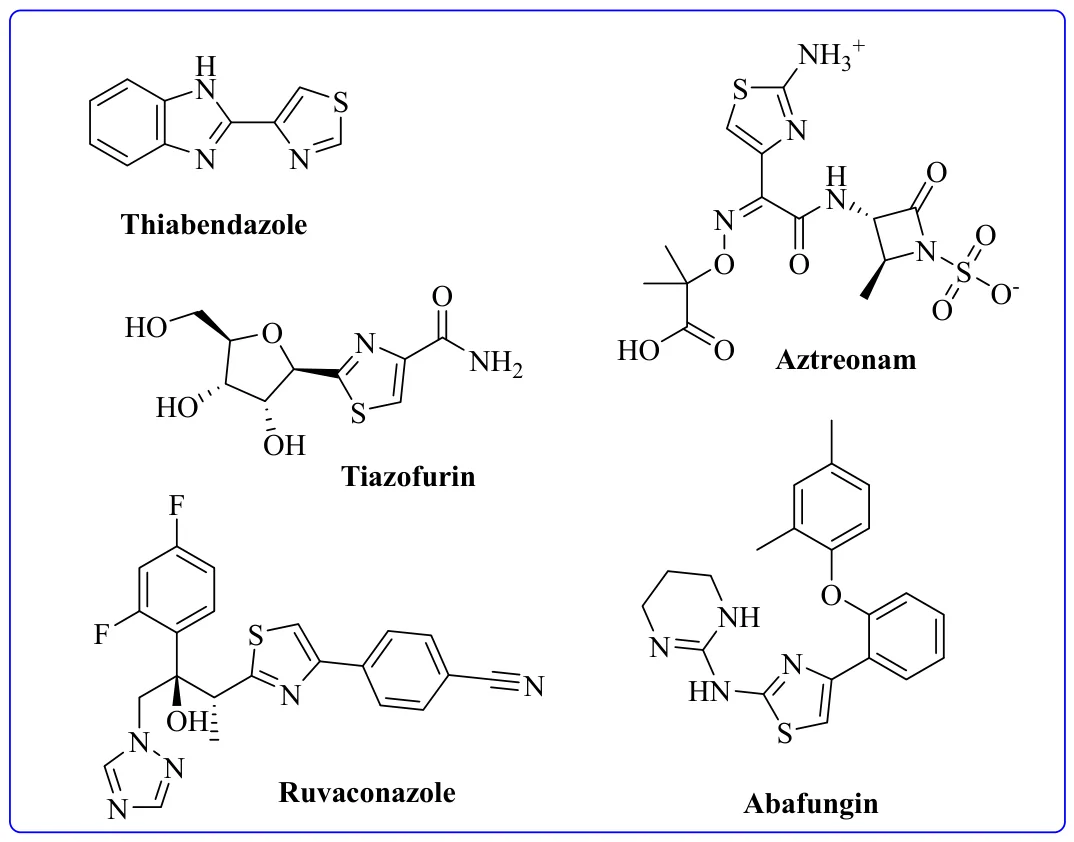
Commercially available drug containing thiazole skeleton.

Despite the considerable progress achieved in the development of RSK inhibitors, substantial off-target kinase interactions and many reported candidates continue to exhibit isoform selectivity, although their therapeutic applicability remains restricted. The irreversible C-terminal kinase domain (CTKD), directed by inhibitors such as FMK, has demonstrated enhanced selectivity through covalent target engagement. Its pharmacological effectiveness remains constrained as CTDK inhibition may be insufficient once activation of the N-terminal kinase domain (NTKD) has occurred, especially within the RSK4-dependent signaling axis [29,30,31]. These challenges highlight the persistent need for the rational design of structurally optimized small molecules with superior selectivity characteristics, enhanced inhibitory efficacy and favorable pharmacokinetic attributes. Nitrogen-containing heterocyclic scaffolds constitute a fundamental class of pharmacophores in contemporary medicinal chemistry because of their extensive structural versatility and diverse biological activities. Among these privileged frameworks, thiazole-clubbed pyrimidine-linked hydrazone derivatives have emerged as potent candidates because of their widespread occurrence in bioactive natural products and therapeutic candidates [32,33,34]. Thiazole-based molecules have been extensively reported to possess a broad spectrum of pharmacological properties, such as anti-microbial, anticancer, antidiabetic, anti-inflammatory, antihypertensive, analgesic and anti-Alzheimer’s activities. Clinical relevance of this heterocyclic motif is further substantiated by its incorporation into several approved candidates, such as triazofurin, thiabendazole, ruvaconazole, abafungin and aztreonam, further highlighting their pharmaceutical significance in drug discovery and development [35,36], as shown in Figure 2.

Inspired by the structural architecture of previously reported RSK inhibitors, the current investigation was directed toward the rational design and optimization of a novel class of thiazole-clubbed pyrimidine-linked hydrazone hybrids as potential RSK4-targeted anticancer candidates. The molecular-design strategy involved the incorporation of structurally diverse substituted thiazole motifs together with systematic modulation of peripheral substituents to fine-tune lipophilic, electronic and steric characteristics of hybrids. These modifications were anticipated to strengthen ligand–target interactions within the ATP-contact cleft of RSK4 and thereby enhance target engagement [37,38]. Particular attention was devoted to preserving key heteroaromatic pharmacophoric elements implicated in kinase recognition and hydrogen-bond interactions while simultaneously modifying molecular adaptability and molecular binding affinity. Systematic structural diversification was undertaken to establish meaningful structure–activity relationships (SAR) and identify favorable molecular determinants associated with enhanced RSK4 inhibitory potency.

A series of novel pyrimidine-linked hydrazone-based thiazole hybrids was synthesized and comprehensively evaluated for RSK4 inhibitory activity via an integrated experimental and computational workflow. Biological assessment was complemented by in silico investigations, such as molecular docking, molecular dynamics simulations, ADMET and ProTox-3.0, to elucidate interaction modes, intermolecular interaction patterns, conformational stability and drug-likeness attributes. These results highlight the therapeutic potential of this hybrid heterocyclic framework and establish a potential structural platform for the future development of potential RSK4-targeted therapeutics for the treatment of esophageal squamous cell carcinoma. This study was designed to identify potent RSK4-targeted lead development through an integrated experimental and computational approach while acknowledging that cellular anticancer activity and pathway-level mechanistic validation in ESCC require further experimental validation in future investigations.

## 2. Results and Discussion

### 2.1. Chemistry

**Scheme 1:**
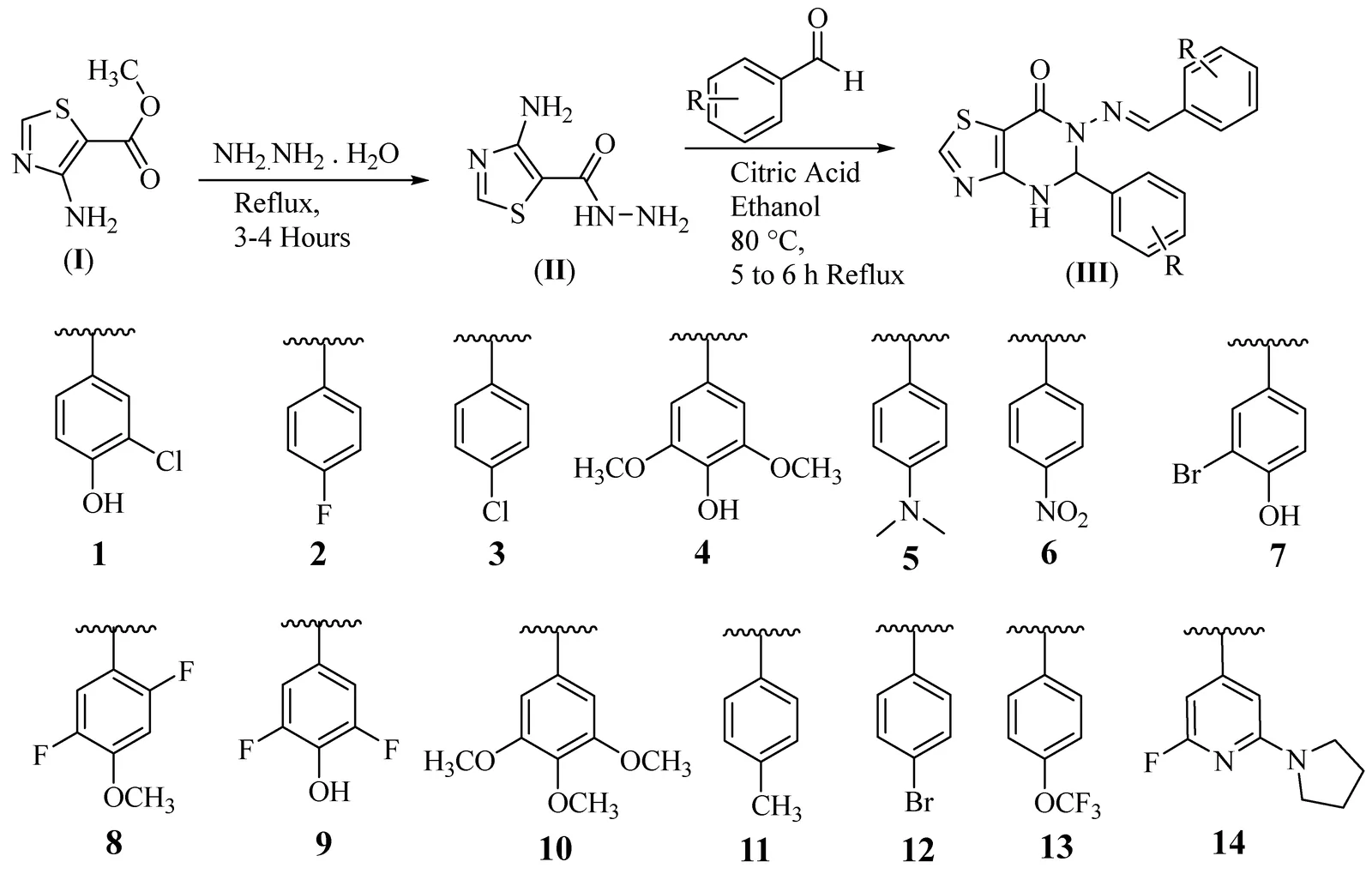
The synthetic route for the preparation of novel thiazole-clubbed pyrimidine-linked hydrazone conjugates **1–14**. The substituents (**R**) on the benzene ring corresponding to each synthesized derivative are also specified in the scheme to facilitate the identification and a comparison of the compounds.

Methyl 4-aminothiazole-5-carboxylate (**I**) (4.86 g 22 mmol) was subjected to hydrazinolysis using excess hydrazine monohydrate (16 mL) (110 °C, oil bath) with continuous stirring for 4 h under reflux and afforded the key intermediate 4-aminothiazole-5-carbohydrazide (II) with a % yield of 85 in an amount of 4.12 g. The mixture was subsequently allowed to cool to room temperature (25 °C) over 45 min, and the precipitated solid was isolated by vacuum filtration through a Buchner funnel. The reaction mixture was washed sequentially with cold petroleum ether to remove residual hydrazine and non-polar impurities. After that, the reaction mixture was dried under ambient conditions to afford the 4-aminothiazole-5-carbohydrazide (**II**) precursor as a pale-yellow solid. The solid precipitate was carried forward to the subsequent step without further purification. The dihydrothiazolo[4,5-d]pyrimidin-7(4H)-one framework was assembled through a three-component reaction of the 4-aminothiazole-5-carbohydrazide (**II**) precursor (1 g for each) with two equivalents of substituted aromatic benzaldehyde (5 mmol, 2.0 equivalent) in absolute ethanol (30 mL). The reaction mixture was under reflux conditions in the presence of citric acid (15 mol%) and ethanol to furnish the corresponding thiazole-clubbed pyrimidine-linked hydrazone (**III**). Citric acid was selected as a mild acid catalyst in ethanol based on its reported effectiveness in hydrazone condensation reactions and its favorable performance compared with conventional acid catalysts in related synthetic systems. The catalyst citric acid and ethanol give excellent eco-friendly yields with good yields (83–93%) [39]. The condensation reactions were continued for 5–6 h. The progress of reaction was monitored through TLC, employing n-hexane/ethyl acetate (7:3 and 8:2 *v*/*v*) as the eluent system. Upon confirmed completion, the reaction mixture was cooled to room temperature (25 °C) over 30 min and further chilled in an ice bath (0–5 °C) for 30 min to maximize product precipitation. The resulting solid was collected by vacuum filtration through a Buchner funnel. The precipitate was washed with cold absolute ethanol and acetone followed by pet-ether to remove unreacted aldehyde, residual citric acid, and non-polar impurities. The ppt was subsequently recrystallized from hot absolute ethanol (20–25 mL) by slow cooling first to room temperature and then to 0–5 °C overnight, yielding the target final products that are discussed below in Scheme 1.

In the FTIR spectra of the synthesized compounds, the NH stretching vibrations appeared in the range of 3331–3237 cm^−1^, while the C=O stretching vibrations of the amide were observed at 1658–1636 cm^−1^. The characteristic CH=N stretching bands were detected at 1611–1580 cm^−1^. In the ^1^H NMR spectra, the peak of amine N=CH protons gave a singlet at δ 9.29–8.21 ppm, integrating for one proton in each case. The NH proton of pyrimidine signals was observed at δ 6.89–7.72 ppm in DMSO-*d*_6_. The CH proton at position 2 of the pyrimidine ring appeared as a singlet in the δ 6.51–6.00 ppm range. The ^13^C-NMR spectra showed the C=O carbons resonating at δ 164.9–158.5 ppm, while the HC=N carbons appeared at δ 156.1–145.1 ppm. The CH carbons at position 2 of the pyrimidine ring were observed at δ 90.4–79.5 ppm.

### 2.2. In Vitro Biological Activities

During the second phase of structural optimization, a pyrimidine ring was introduced in the hydrazide along with a hydrazone core that was retained to preserve the favorable pharmacophoric framework. The systematic modifications were introduced at the 5 and 6 position of the pyrimidine ring to investigate the influence of electronic and steric variations on RSK4 inhibitory activity. Guided by this strategy, a structurally diverse series of pyrimidine functionalized derivatives (**1**–**14**) was synthesized and evaluated against RSK4 in-vitro. The synthesized analogues displayed a broad range of inhibitory activities, with IC_50_ (nM) values spanning from 15.32 ± 1.35 to 54.61 ± 2.17, relative to the reference inhibitor BI-D1870 IC_50_ (nM) = 33.16 ± 1.34. The present in vitro study showed the strong sensitivity of biological activity to peripheral substituent modulation. Novel candidates showed potent biochemical inhibition of RSK4, and their broader kinase selectivity cannot be established from the current single-target assay [39,40].

Among all the synthesized scaffolds, compound **9**, containing a 3,5-difluoro-4-hydroxy substitution, exhibited the highest inhibitory potential, with an IC_50_ value of 15.32 ± 1.35 nM. The remarkable potency of compound-**9** may be attributed to the synergistic electronic effect of two fluorine atoms together with the hydroxy group and hydrogen-bond-donating group. The fluorine atom and hydroxyl group promotes enhanced bonding affinity due to a more favorable hydrogen-bonding binding orientation, with the ATP-binding region of RSK4 having key amino acid residues. Similarly, compound **2**, which constitutes para-fluoro substitution and has an IC_50_ value of 17.08 ± 1.65 nM, also demonstrated promising inhibitory potential as compared to the reference inhibitor but lower than compound **9**. A fluorine group is highly favorable in binding interactions with RSK4 inhibition because of the high electronegativity, small atomic size and strong electron-withdrawing nature of fluorine. However, -F atoms likely optimize hydrophobic interaction and improve kinase binding orientation of the synthesized analogues [40].

Similarly, compound **14**, which has a substation-pattern 2-fluoro-6-(pyrrolidine-1-yl)pyridine skeleton, has demonstrated potent RSK4 inhibitory potential as compared to the reference inhibitor. The enhanced inhibitory potential of this moiety may be attributed to the combined influence of the fluorinated heteroaromatic pyrrolidine and pyridine ring. The pyridine ring is likely to contribute favorable electronic effects that improve lipophilic interaction which leads to stabilizing ligand binding affinity towards the RSK4 kinase active site. However, compound **14** has lower inhibitory potential as compared to compounds **2**, **8**, **9** and **13**, possibly due to increased steric bulk introduced by the pyrrolidine that hinders optimal binding orientation.

Further structural refinement demonstrated that compound **8**, possessing a 2,5-difluoro-4-methoxy group, also showed good potency as compared to compound **13** and as well as the reference inhibitor but showed lower potency as compared to compound **9**, because hydroxy substitution is more beneficial than methoxy substitution for RSK4 due to its strong hydrogen-bonding potential. The combined effect of difluoro substitution and electron-donating methoxy functionality would improve both electronic and hydrophobic interactions. A comparative evaluation of compound **13** showed that replacement of the para-trifluoromethoxy group with a para-hydroxyl substituent produced the most potent analogue in the series. The enhanced activity associated with this derivative may be attributed to the fact that -OCF_3_ is highly lipophilic and has strong electron-withdrawing capability. The Van der Waals interaction likely facilitated stronger non-covalent interactions and improved stabilization within the catalytic binding pocket.

Additional SAR investigations involving halogenated derivatives demonstrated that compounds **1** and **3** exhibited notable RSK4 inhibition, with IC_50_ values of 22.87 ± 1.93 and 24.13 ± 1.08 nM, respectively. Among these analogues, compound **1**, bearing a meta-chloro and para-hydroxyphenyl substituent, displayed superior activity compared to the mono-chloro derivative **3**. The SAR indicates that the increased hydrogen-bonding interaction of hydroxy with RSK4 enzymes and the electron-withdrawing characteristic associated with chloro substitution enhance target affinity and inhibitory efficiency. Similarly, compound **7**, containing 3-bromo-4-hydroxy substitution, showed reduced activity as compared to the reference inhibitor but higher activity as compared to the para-bromine substitution of compound **12**. The decreased potency of brominated compounds as a result of steric hinderance caused by the high polarizability of bromine atoms caused adverse effects on the optimal fitting inside the RSK4 active pocket [41].

Electron-donating groups, such as -OCH_3_ and -CH_3_ groups, displayed moderate activity. Compound **10**, with 3,4,5-trimethoxy, showed a lower potency value as compared to compound **4**, because compound **4** has a hydroxyl group that showed hydrogen bonding that enhanced the biological activity of synthesized analogues as compared to the reference inhibitor. Similarly, electron-donating methoxy-containing derivatives displayed moderate activity.

Hydroxyl groups can enhance RSK4 recognition through hydrogen interactions, and their contribution is context dependent. A higher potency of compound **8**, with an IC_50_ of 18.08 ± 0.99 nM relative to compound **4** at 27.25 ± 1.89 nM, indicates that overall electronics and complementarity may outweigh hydrogen interaction capacity alone. Hydrogen interactions should be considered as a contributor rather than a dominant determinant of RSK4 inhibitory activity [42,43].

Compound **11**, containing a methyl substitute, demonstrated appreciable activity as compared to the reference inhibitor, although its potency remained higher than that of the analogue **5** and reference inhibitor. In contrast, compound **5**, incorporating di-methyl amine substituents, generally exhibited weak or negligible inhibition comparable to the reference standard, with no statistically significant difference between the two treatments (*p* > 0.05). These functionalities are less compatible with the steric and electronic requirements of the RSK4 binding cavity. The reduced activity observed for these derivatives may result from unfavorable conformational effects or disruption of critical ligand–protein interactions necessary for effective kinase inhibition (Table 1).

As a result, the SAR analysis demonstrated that RSK4 inhibitory activity was strongly affected by the position, nature and size of the substituent in the aryl ring of the thiazole–pyrimidine skeleton. Electron-withdrawing groups, particularly fluorinated atoms, significantly enhanced biological activity. The presence of hydroxy groups further improved activity through possible hydrogen-bonding interactions, whereas bulky substitutions such as -Br, -OCH_3_, and -N(CH_3_) reduced activity due to steric hinderance and moderate lipophilicity [44].

### 2.3. Molecular Docking Study

The molecular binding profile of the novel series against RSK4 (PDB ID: 6G77) was systematically investigated through blind molecular docking analysis. Ligands were allowed to explore all accessible interacting regions and favorable affinity poses were selected for interaction evaluation. Several candidates that occupied the canonical ATP-interacting pocket (compound **9**) adopted alternative contact sites and showed interactions with different amino acid residues. All evaluated ligands exhibited a well-accommodation fit within the enzyme’s active site, consisting of energetically favorable and conformationally stable binding poses. These interactions were primarily stabilized via an intricate interplay of hydrogen contact, π-π contacts and related aromatic interactions, and extensive hydrophobic interactions that collectively contribute to robust ligand–target recognition. The core scaffold of the novel series demonstrated a consistent and well-aligned orientation within the catalytic cavity, which enabled efficient engagement with catalytically and structurally relevant residues. Key amino acid residues were repeatedly implicated in ligand recognition and stabilization across the dataset. This recurrent residue involvement highlights their pivotal role in governing substrate accommodation and inhibitor contact within the active-site architecture of ribosomal S6 kinase 4 **(**Table 1). The consistent participation of these residues across multiple interactive poses further underscores their significance as primary-anchor determinants for pharmacophoric interactions, with the catalytic cleft being the principal inhibitory hotspot responsible for the modulation of (RSK4) activity.

The molecular docking scores did not fully re-capitative the experimental RSK4 potency, and compound **8** showed a most favorable score of −10.2 kcal/mol, while compound **9** exhibited the highest inhibitory potency at IC_50_ = 15.32 nM, with a weaker predicted score of −7.2 kcal/mol. This divergence highlights the qualitative nature of scores which are not direct surrogates for experimental IC_50_ values and are primarily used to elucidate plausible contact modes and key complex interactions, while differences in scores may arise from the simplified treatment of solvation, flexibility and other energetic contributions.

Molecular docking was performed against the crystal structure of the human RSK4 kinase domain with PDB ID: 6G77 at a resolution of 2.50 Å. Crystallographic water was removed, and the native ligands were saved as L1, L2, L3 and L4 for the docking validations, and ligands were geometrically optimized and appropriately converted to PDBQT format. The molecular grid was centered and covered the complex within the grid box.

**Figure 3:**
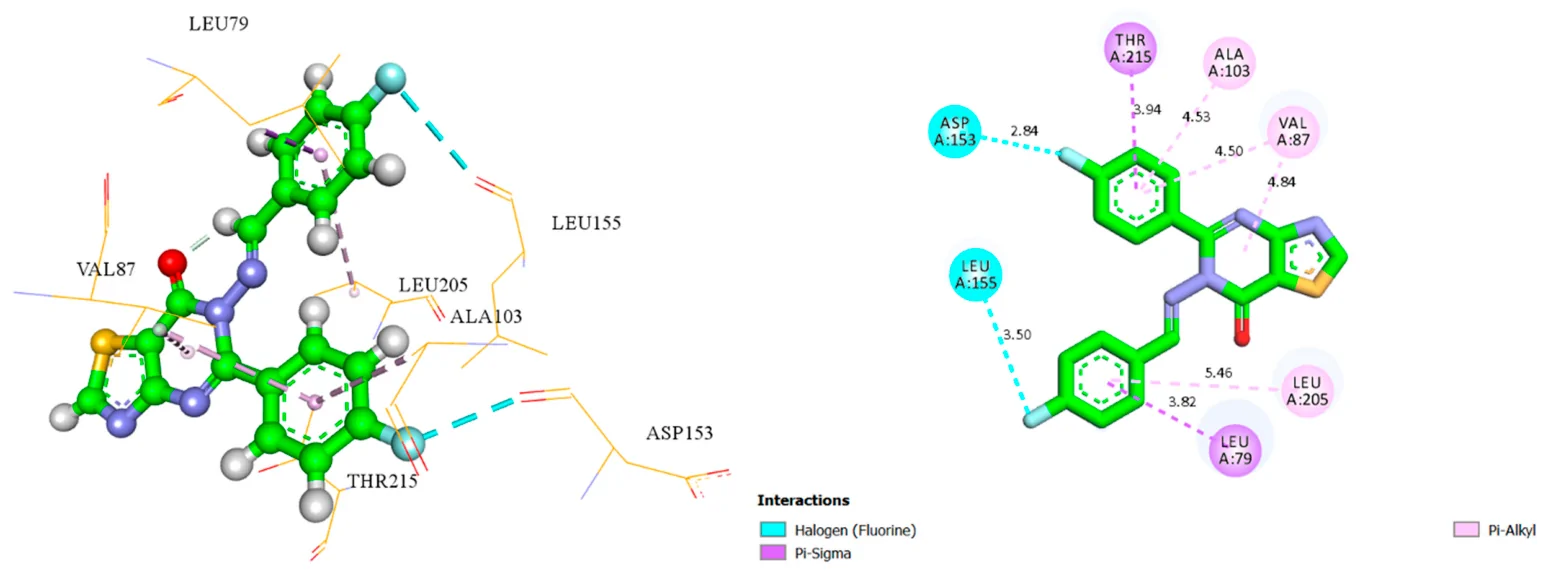
Predicted molecular docking analysis of novel compound **2** interactions within the catalytic pocket of RSK4 (**left**). A two-dimensional schematic representation of the ligand–protein interaction complex, in which the principal intermolecular contacts stabilize the complex, is presented in the (**right**) panel.

Compound **2** adopts an extended contact conformation spanning the catalytic cavity that enabled multivalent interactions across the active-site architecture and showed a favorable molecular binding affinity of −9.5 Kcal/mol and interacted predominantly through hydrophobic (Leu205, Val87 and Ala103) and halogen-mediated interactions (Asp153 and Leu155). The absence of classical hydrogen bonds was compensated by multiple interactions which effectively stabilized the ligand within the active site. These interactions suggest that hydrophobic complementarity played a dominant role in governing the contact-orientation behavior of this novel candidate (Figure 3).

**Figure 4:**
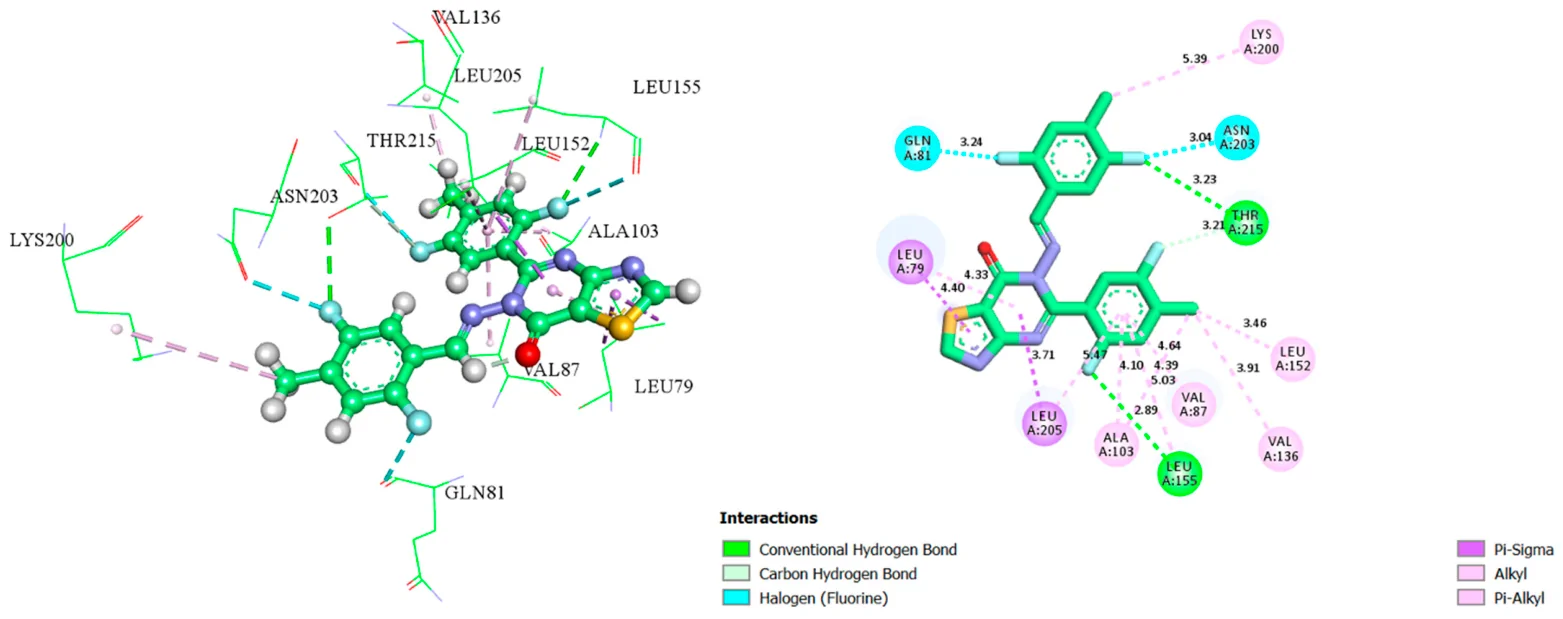
Predicted molecular docking analysis of novel compound **8** interactions within the catalytic pocket of RSK4 (**left**). A two-dimensional schematic representation of the ligand–protein interaction complex, in which the principal intermolecular contacts stabilize the complex, is presented in the (**right**) panel.

Figure 4 delineates the molecular docking poses and associated interaction profiles and shows that compound **8** orients aromatic pharmacophore in close proximity and exhibited the highest molecular binding affinity (−10.2 Kcal/mol), showing the most favorable interaction with the target. The superior molecular interaction affinity of this novel candidate was attributed to a well-balanced network of interactions to facilitate pronounced two-conventional hydrogen bonds with Leu155 and Thr215 at distances of 2.89 and 3.23 Å and one additional carbon–hydrogen bond with Thr215 (3.15 Å) within the catalytic microenvironment. The ligand engages to stabilize hydrophobic interactions including sub-classes of π–alkyl contacts with Lys200, Leu152, Val136, Ala103 and Val87 and two π–sigma (Leu79 and Leu205) interactions collectively. The hydrophobic milieu defined by these residues further contributes to conformational stabilization that enables compound **8** to adopt a compact, energetically optimized contact geometry that closely emulates the native substrate-bound conformation. The co-existence of these interactions, including conventional hydrogen bonds, carbon–hydrogen bonds and extensive hydrophobic contacts, contributed to the stabilization of the ligand within the active-site cavity, and this supports its optimal contact orientation and strong receptor complementarity (Figure 4).

**Figure 5:**
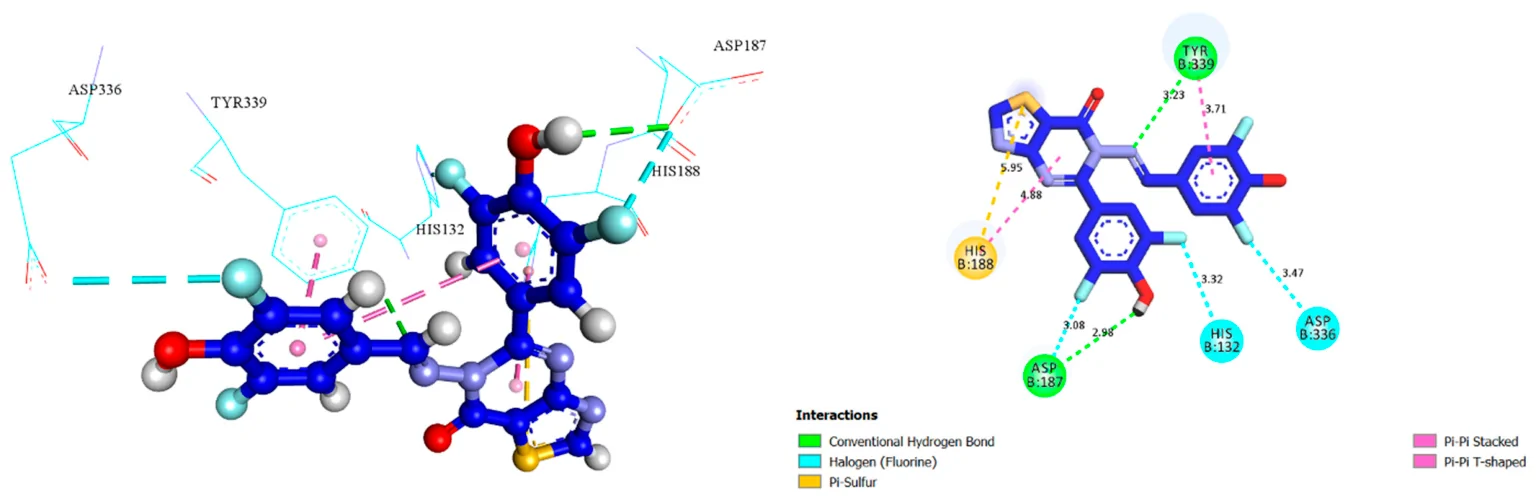
Predicted molecular docking analysis of novel compound **9** interactions within the catalytic pocket of RSK4 (**left**). A two-dimensional schematic representation of the ligand–protein interaction complex, in which the principal intermolecular contacts stabilize the complex, is presented in the (**right**) panel.

The novel compound **9** exhibited the lowest affinity profile (−7.2 Kcal/mol) among the evaluated compounds. The ligand formed a diverse array of intermolecular interactions, like conventional hydrogen bonds (Tyr339 and Asp187) as well as one π–sulfur (His188), three halogen (His132, Asp336, and Asp187) and two π-π stacked and π-π T-shaped (His188 and Tyr339) interactions. In spite of this broad interaction profile the comparatively weaker molecular binding affinity score suggests that the spatial arrangement and cumulative strength of these interactions were less favorable than those observed for the higher-ranked scaffolds (Figure 5).

**Figure 6:**
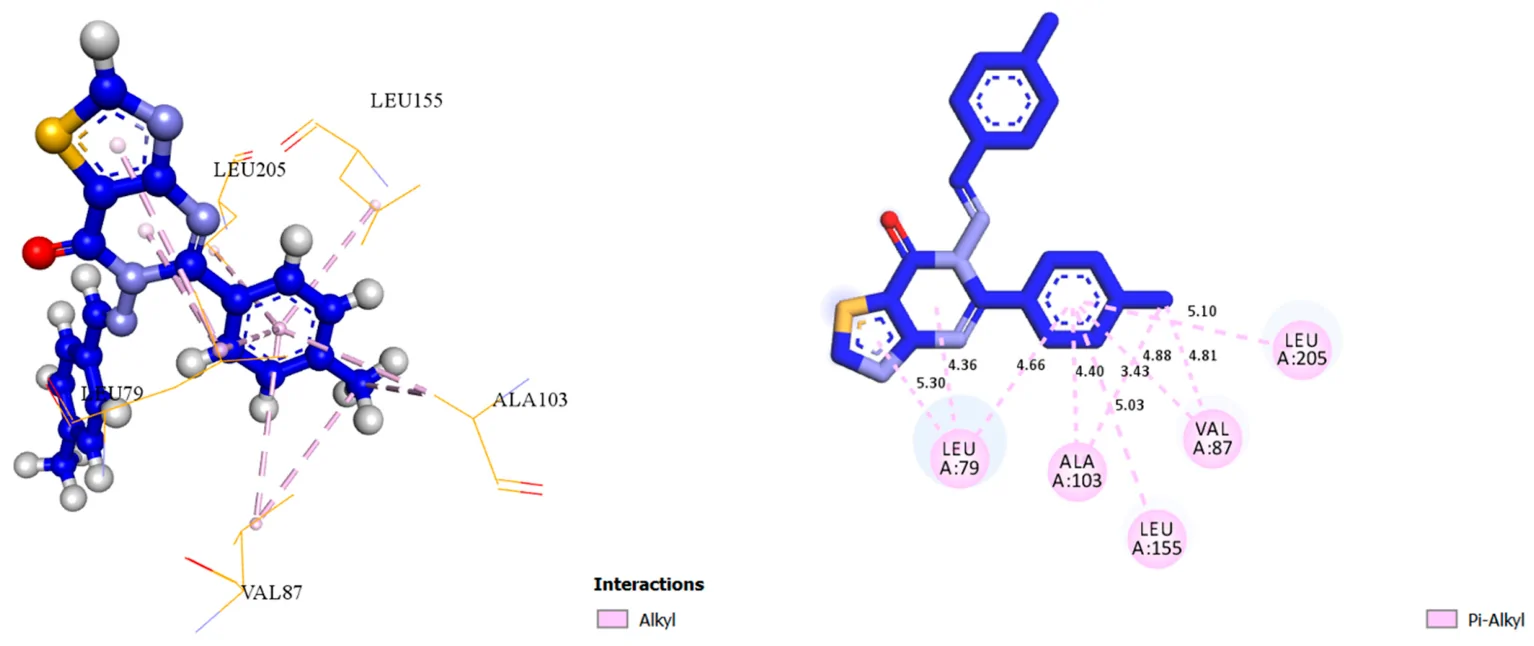
Predicted molecular docking analysis of novel compound **13** interactions within the catalytic pocket of RSK4 (**left**). A two-dimensional schematic representation of the ligand–protein interaction complex, in which the principal intermolecular contacts stabilize the complex, is presented in the (**right**) panel.

The novel drug **13** displayed a molecular binding affinity of −9 Kcal/mol and was primarily stabilized through hydrophobic interactions. The formation of several hydrophobic interactions with Leu205, Val87, Leu155, Ala103 and Leu79 indicated efficient occupancy of the hydrophobic regions of the active site and supported a stable conformational binding orientation despite the lack of hydrogen interactions (Figure 6).

**Figure 7:**
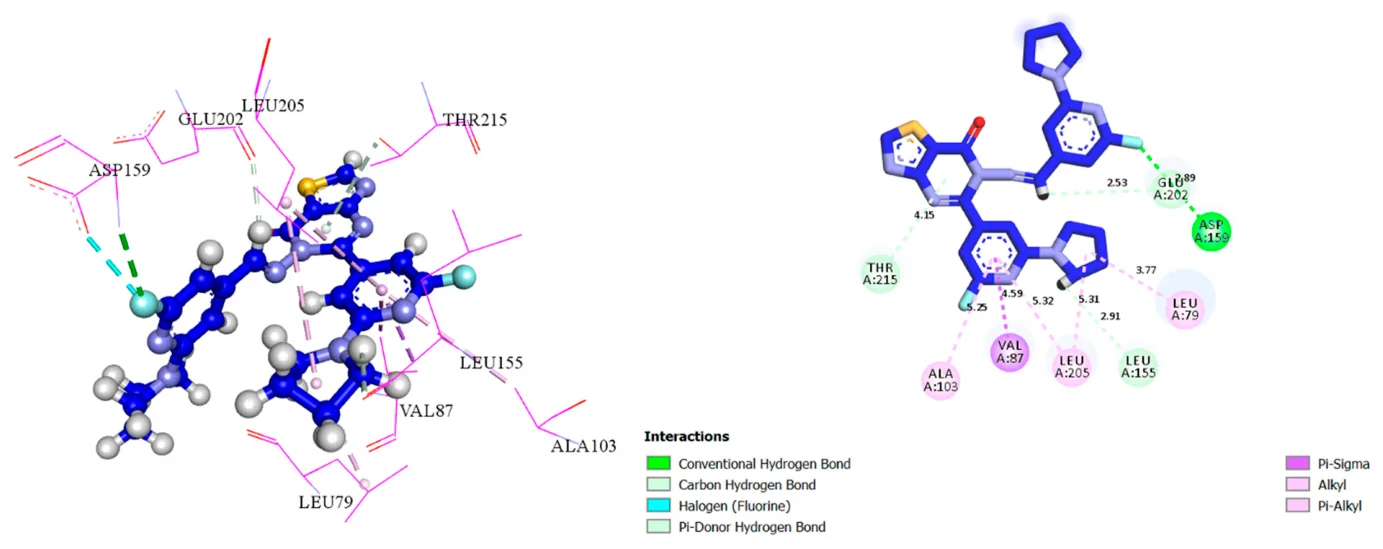
Predicted molecular docking analysis of novel compound **14** interactions within the catalytic pocket of RSK4 (**left**). A two-dimensional schematic representation of the ligand–protein interaction complex, in which the principal intermolecular contacts stabilize the complex, is presented in the (**right**) panel.

Compound **14** demonstrated a docking score of −10 Kcal/mol, and a distinct contact orientation is evident, wherein the ligand establishes a more direct engagement with key catalytic residues. The ligand established a combination of one conventional hydrogen bond with Asp159 at distance of 2.89 Å, and three additional carbon hydrogen bonds and pi-donor hydrogen bonds (Thr215, Leu155, and Glu202) underscore robust hub interactions within the catalytic pocket, which collectively enhanced the stability of the protein–ligand complex. The presence of multiple hydrophobic interactions further strengthened the molecular intervention mode (Figure 7). These contacts further facilitate spatial accommodation and enhance complementarity within the cavity. The co-existence of persistent hydrogen interactions and hydrophobic stabilization suggests that compound **5** may exhibit elevated affinity alongside appreciable target selectivity towards RSK4.

**Figure 8:**
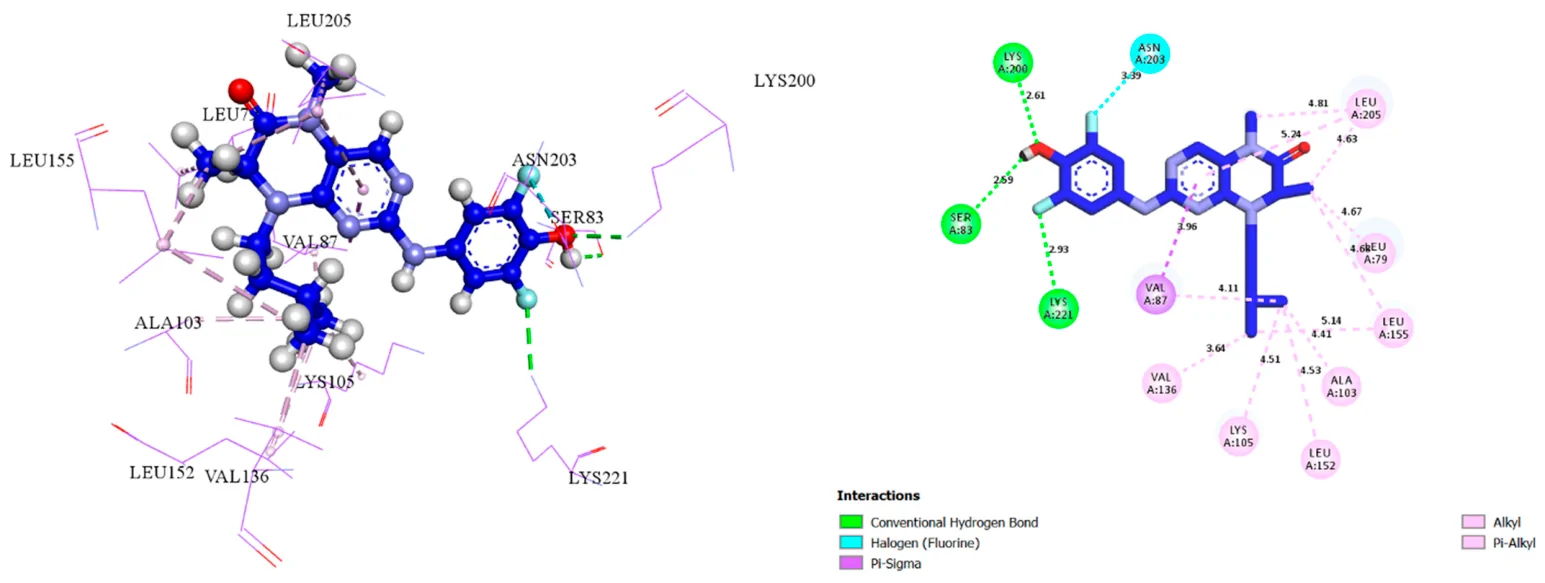
Predicted molecular docking analysis of reference inhibitor interactions within the catalytic pocket of RSK4 (**left**). A two-dimensional schematic representation of the ligand–protein interaction complex, in which the principal intermolecular contacts stabilize the complex, is presented in the (**right**) panel.

The reference inhibitor exhibited a molecular docking score of 9.8 Kcal/mol and formed an extensive interaction network characterized by multiple conventional hydrogen bonds and abundant hydrophobic contacts. The reference inhibitor interacted predominantly through three conventional hydrogen bonds with Lys200, Ser83 and Lys221 at distances of 2.61, 2.59, and 2.93 Å and abundant pi–alkyl interactions with Leu205, Leu79, Leu155, Ala103, Leu152, Lys105, Val136 and Val87. Although the reference inhibitor displayed strong ligand–protein recognition and favorable stabilization within the active site, its molecular binding energy remained slightly lower than those observed for the novel drugs 14 and 8. These results show the potential superiority of the newly designed candidates (Figure 8).

Among the investigated analogues, compound **8** exhibits the most favorable interaction landscape. Its deep insertion into the catalytic pocket enables a strong electrostatic-engagement bridge-like interaction that substantially enhances complex stabilization. The hydrophobic contacts further consolidate its orientation within the active site. The synergistic integration of contact forces culminates in a highly cooperate interactive network, supporting the proposition that compound **8** may function as a superior inhibitor relative to the other candidates. Contact maps were consistently identified as a principal hub in stabilization within the catalytic environment, whereas they appear to serve as secondary yet structurally contributory interaction nodes. Compounds **8** and **14** emerge as potential candidates, which is attributable to their ability to establish dense, multipoint interaction networks, e.g., conventional hydrogen bonds, π-π contact, electrostatic stabilization and hydrophobic interactions. Collectively, these interaction profiles strongly substantiate the potential of novel candidates as potent (RSK4) inhibitors with high affinity and strong active-site complementarity.

For further validation of the molecular docking protocol the four co-crystallized native ligands (L1, L2, L3 and L4) associated with the target protein were re-docked into the cavity. The native ligands exhibited a molecular contact affinity ranging from −7.4 to 8.6 Kcal/mol, which was notably lower than that observed for the top-ranked-designed compounds. These results suggest that the novel synthesized derivatives possess enhanced potential and stronger engagement compared with the native ligands, as shown in Table 2 and Table 3 and the supplementary files (Table S5, Figures S1–S4).

### 2.4. ADMET Analysis

#### 2.4.1. Physicochemical Profile and Structural Complexity

The physicochemical properties of drug candidates play a pivotal role in determining their pharmacological behavior, overall developability and molecular recognition characteristics. A comparative assessment of the novel synthesized candidates (**2**, **8**, **9**, **13** and **14**) against the reference inhibitor revealed notable differences in structural architecture and molecular complexity. The reference inhibitor exhibited a highly saturated chemical framework characterized by a favorable fraction of Fsp3 (0.421). Fsp3 is a parameter that is frequently associated with enhanced 3-dimensionality, reduced attrition and improved solubility within drug development. Compound **14** demonstrated the closest resemblance to this desirable structural feature, with an Fsp3 of 0.375, which is attributable to the incorporation of a conformationally flexible moiety.

#### 2.4.2. Molecular Optimization and Drug-Likeness

An evaluation of molecular desirability parameters demonstrated that several members of the synthesized series possess favorable drug-like profiles. Compound **2** achieved a favorable quantitative estimate of drug-likeness, with a QED of 0.709, which approaches the value recorded for the reference inhibitor at a QED of 0.760. This favorable score can be attributed to its balanced physicochemical characteristics, e.g., a moderate molecular weight of 370.07 g.mol^−1^, and well-optimized lipophilicity parameters of log P (2.61) and log D (2.90), which collectively support efficient membrane permeation while decreasing excessive hydrophobicity. Compounds **13** and **14** occupied the upper boundary of the conventional drug-like chemical space, with molecular weights >500 g.mol^−1^. Although these larger molecular frameworks may offer enhanced target interactions through expanded pharmacophoric features, they may also introduce challenges associated with oral bioavailability and systemic exposure, and this warrants further pharmacokinetic optimization.

#### 2.4.3. Pharmacokinetic Adaptability

Successful translation of a bioactive molecule into a therapeutic agent requires not only potent target engagement but also favorable pharmacokinetic behavior. The synthesized compounds exhibited high absorption and distribution characteristics, and computational permeability analyses revealed consistently high transcellular diffusion capabilities across all investigated scaffolds.

Compounds **2**, **8**, **13** and **14** match the moderate tissue distribution of the reference inhibitor (VD 0.17 to 0.42 L.kg^−1^). Compounds **8**, **9** and **13** are fully excluded, crossing the BBB (0.00). These characteristics are highly attractive for systemic therapeutic applications. The novel drugs maintain low to moderate plasma rates ranging from 4.21 to 6.01 mL.min^−1^.kg^−1^ as were shown in Table 4.

A comparative evaluation of the physicochemical descriptors demonstrated notable variations in molecular lipophilicity and polar surface characteristics among the investigated analogues. The reference drug exhibited a well-balanced drug-like profile, with a molecular weight of 391.18, moderate lipophilicity (log P: 4.172) and a comparatively compact topological polar surface area (TPSA (81.59)). These parameters provided a suitable benchmark to assign the structural behavior of the synthesized scaffolds (1–5).

Among the experimental analogues, compound **2** represents the smallest molecular framework within the series and displays an MW of 370.07. Despite its reduced molecular size, the compound showed pronounced lipophilic behavior (log P: 2.614) together with a relatively low surface area (TPSA: 57.59). Progressive structural modification further intensified this trend. The incorporation of dimethoxy substituents in compound **8** increased the molecular mass to 466.07, accompanied by a marked expansion in TPSA to 76.05.

Compound **9** displayed an alternative substitution architecture that maintained a moderately elevated lipophilic balance (log P: 2.352) while simultaneously rising the TPSA (98.05). This behavior suggests enhanced localized hydrogen-interaction potential and improved intermolecular interaction capacity.

The highest hydrophobic characteristic within the investigated series was observed for compound **13** (MW: 502.05), which exhibited a substantially elevated lipophilicity value (logP: 3.734). Such a profile extends beyond the generally accepted Lipinski drug-likeness threshold and may therefore increase the probability of membrane retention, reduced pharmacokinetic selectivity and non-specific biomolecular interactions.

**Figure 9:**
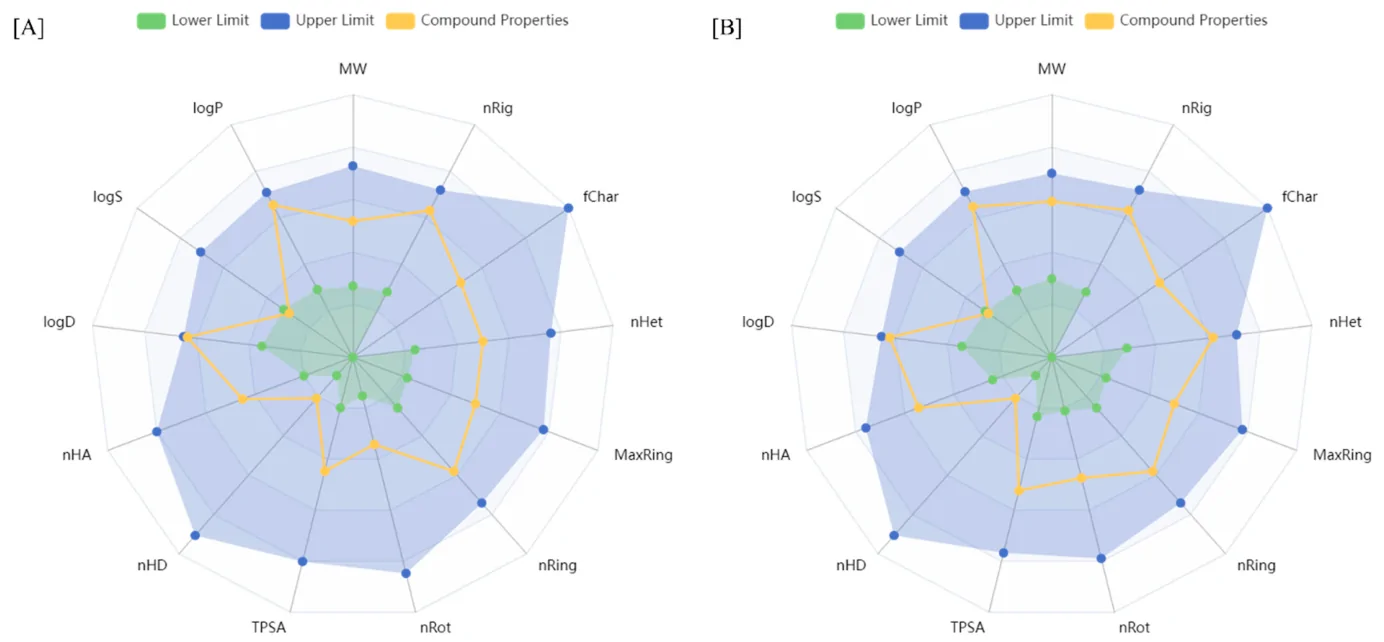
Bioavailability radar plots of novel compound **2** [A] and compound **8** [B] showing physicochemical profiles. The evaluated physicochemical parameters are consistent with accepted oral drug-likeness requirements and support the suitable pharmacokinetic and bioavailability-related profiles.

**Figure 10:**
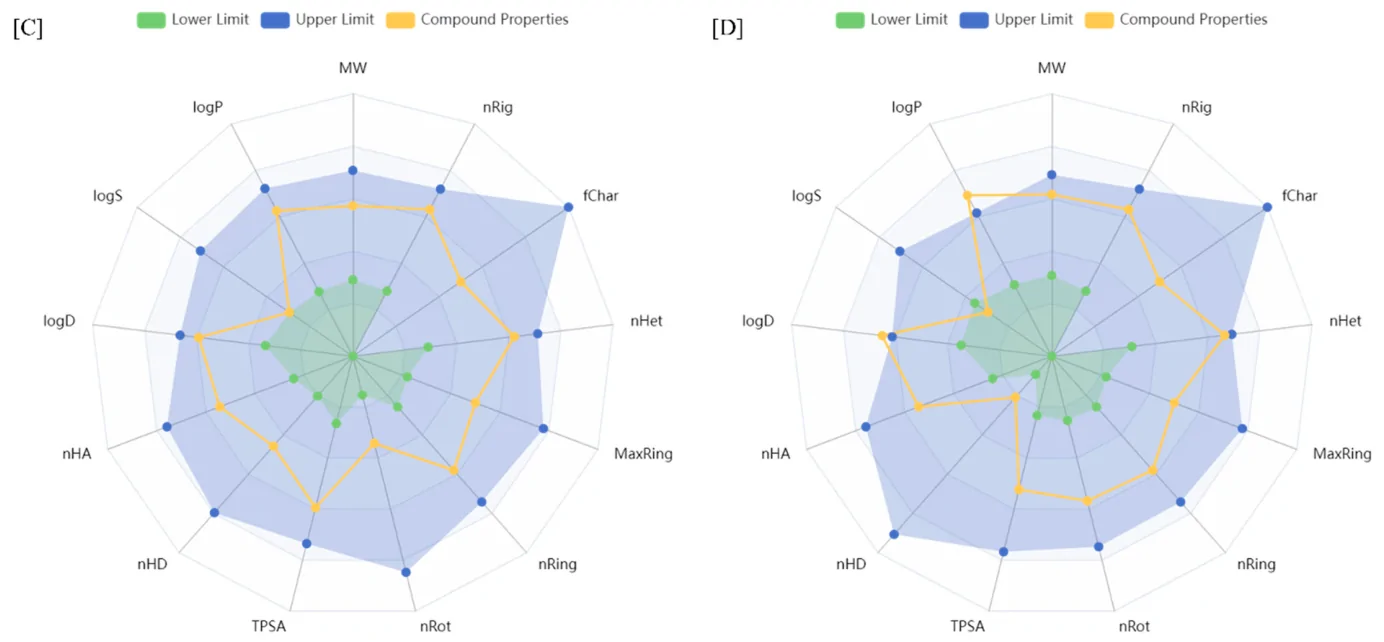
Bioavailability radar plots of novel compound **9** [C] and compound **13** [D] showing physicochemical profiles. The evaluated physicochemical parameters are consistent with accepted oral drug-likeness requirements and support the suitable pharmacokinetic and bioavailability-related profiles.

Compound **14** demonstrated a comparatively optimized physicochemical profile (MW: 510.18). The introduction of basic heterocyclic moieties effectively moderated lipophilicity to a log *p* value of 3.652, although the compound retained a relatively large TPSA (89.85). These results indicate that structural incorporation of heterocyclic functionalities may contribute to balance hydrophobicity while preserving sufficient polarity for favorable pharmacological parameters (Figure 9 and Figure 10, supplementary Figure S32).

In silico ADMET assessment suggests that several of the synthesized candidates possess favorable drug-like and pharmacokinetic characteristics. So, these results should be validated and regarded as experimental pharmacokinetic, toxicological and metabolic studies.

#### 2.4.4. ProTox-3.0 Analysis

**Figure 11:**
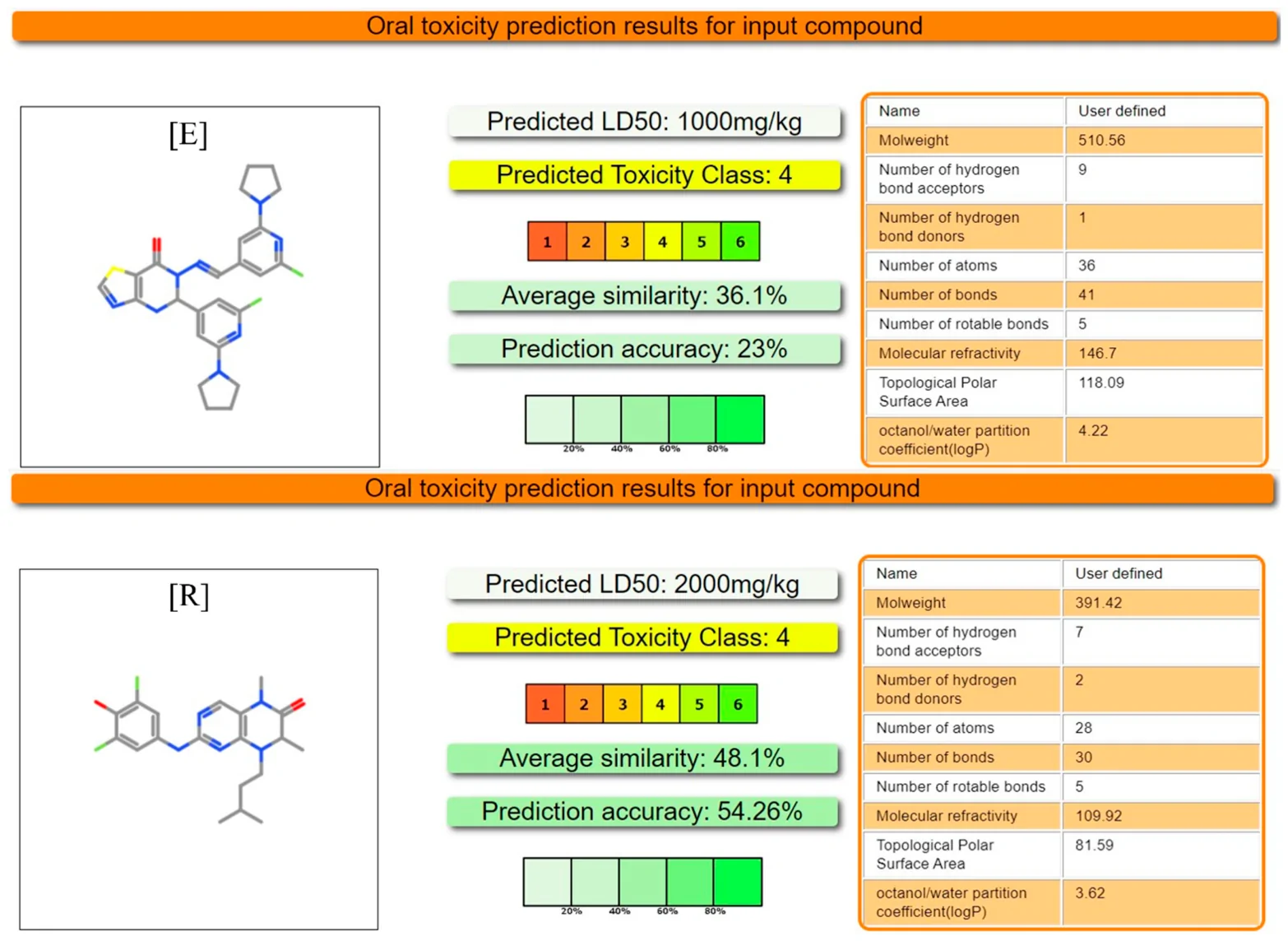
Computational prediction of oral toxicity profiles and toxicity class assignment for novel compound **14** [E] and reference drug [R]. Toxicity assessment provides preliminary insights into the safety profile of the investigated candidates and their prospective suitability for further pharmacological applicability.

In silico toxicity evaluation revealed that all of the designed candidates (**2**, **8**, **9**, **13** and **14**) were classified within toxicity class 4 and showed a favorable safety profile that was comparable to that of the reference inhibitor. The predicted median lethal dose of LD_50_ for all derivatives was 1000 mg/kg and suggests low acute toxicity. The molecular weights of the designed candidates (**2**, **8**, **9**, **13** and **14**) ranged from 370.38 to 510.56 Da, indicating that they are within the acceptable range for drug-like candidates. Moreover, the synthesized candidates possessed 5 to 9 H-bond acceptors and 1 to 3 H-bond donors, while the reference inhibitor showed 7 hydrogen-bond acceptors and 2 hydrogen-bond donors. The number of rotatable bonds varied from 3 to 7, showing moderate conformational flexibility that may facilitate favorable target contacts. The molecular refractivity values ranged from 100 to 146, while the reference inhibitor displayed a molecular refractivity of 109.92. The predicted toxicological and physicochemical parameters suggest that the designed candidates possess favorable drug-likeness profiles and pharmacokinetic attributes that support their being promising therapeutic candidates, as shown in Table 5, Figure 11 and the supplementary files (Figures S33–S41).

#### 2.4.5. Endpoint Specific Analysis: Mutagenicity and Immunotoxicity

An analysis of endpoint-specific toxicological parameters revealed clear differences in mutagenic potential among the investigated compounds. Compounds **2**, **8**, **9** and **13** were predicted to exhibit active mutagenicity profiles, with probabilities of 0.52, 0.57, 0.54 and 0.52. These outcomes may be associated with the presence of structurally reactive motifs. Compound **14** (0.51, inactive) and the reference inhibitor (0.51, inactive) demonstrated no predicted mutagenic liability, which suggests their respective structural frameworks maintain improved genomic safety characteristics, as summarized in Table 6 and the supplementary files (Table S1).

So, immunotoxicity predictions revealed a differentiated safety profile across the novel series, with compounds **8** and **9** predicted to be active, whereas compounds **2**, **13** and **14** were predicted to be inactive. This pattern suggests that the changes in substitution pattern and overall molecular physicochemical properties may influence predicted immunotoxicity profiles. The current dataset does not establish a single structural determinant that is responsible for this difference. The results are therefore best regarded as a preliminary lead optimization signal rather than as definitive structure–immunotoxicity relationships. The structural diversification changed the predicted immunotoxicity characteristics, predicting a potential safety window for further optimization.

The immunotoxicity predictions displayed a distinct distribution pattern across the series. The reference inhibitor showed active immunotoxicity potential, with a probability of 0.72(active), and compound **2** (0.81) and compound **14** (0.74) showed inactive profiles in immunotoxicity, suggesting reduced immune-related adverse liability.

Compound **2** showed a favorable QED value of 0.709, which is close to that of the standard inhibitor at 0.760, and its predicted mutagenicity of 0.52 represents an important liability for further development. Compound **2** should be regarded as a potent but incompletely optimized lead. Future optimization will explore controlled modifications of peripheral substituents, while other structural features associated with RSK4 inhibition, using iterative computational toxicity identification and experimental validation, will be used to predict analogues with improved safety profiles and preserved potency.

#### 2.4.6. Tox-21 Predictive Characteristics

An evaluation of the compounds via the Tox-21 predictive platform demonstrated minimal interactions with major endocrine-related signal networks. All investigated compounds, including the reference inhibitor, were predicted to remain inactive toward the androgen receptor (AR), AR ligand-binding domain (AR-LBD), estrogen receptor alpha and estrogen receptor LBD. This collective inactivity suggests a relatively low probability of disturbing effects.

**Figure 12:**
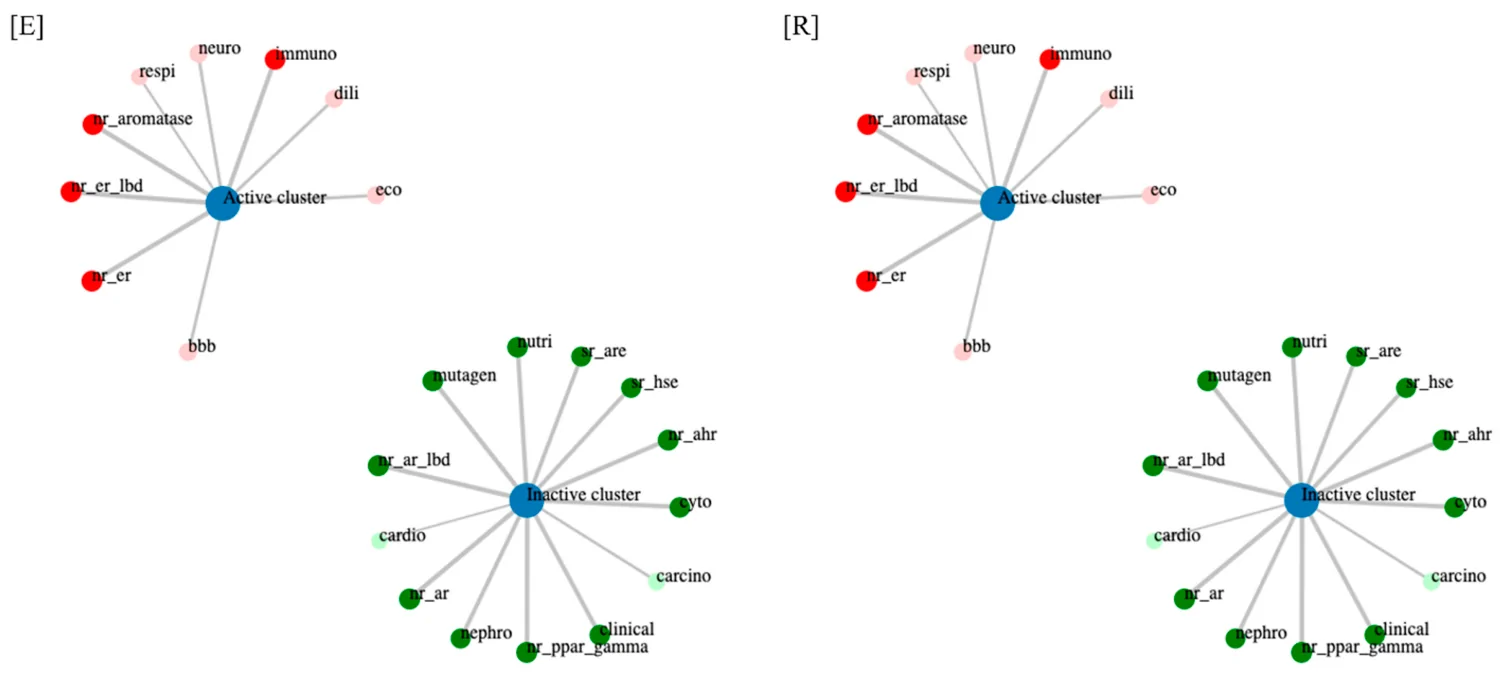
Bioavailability radar plot for the prediction of drug-likeness and pharmacokinetic profiles of novel compound **14** [E] and reference drug [R].

**Figure 13:**
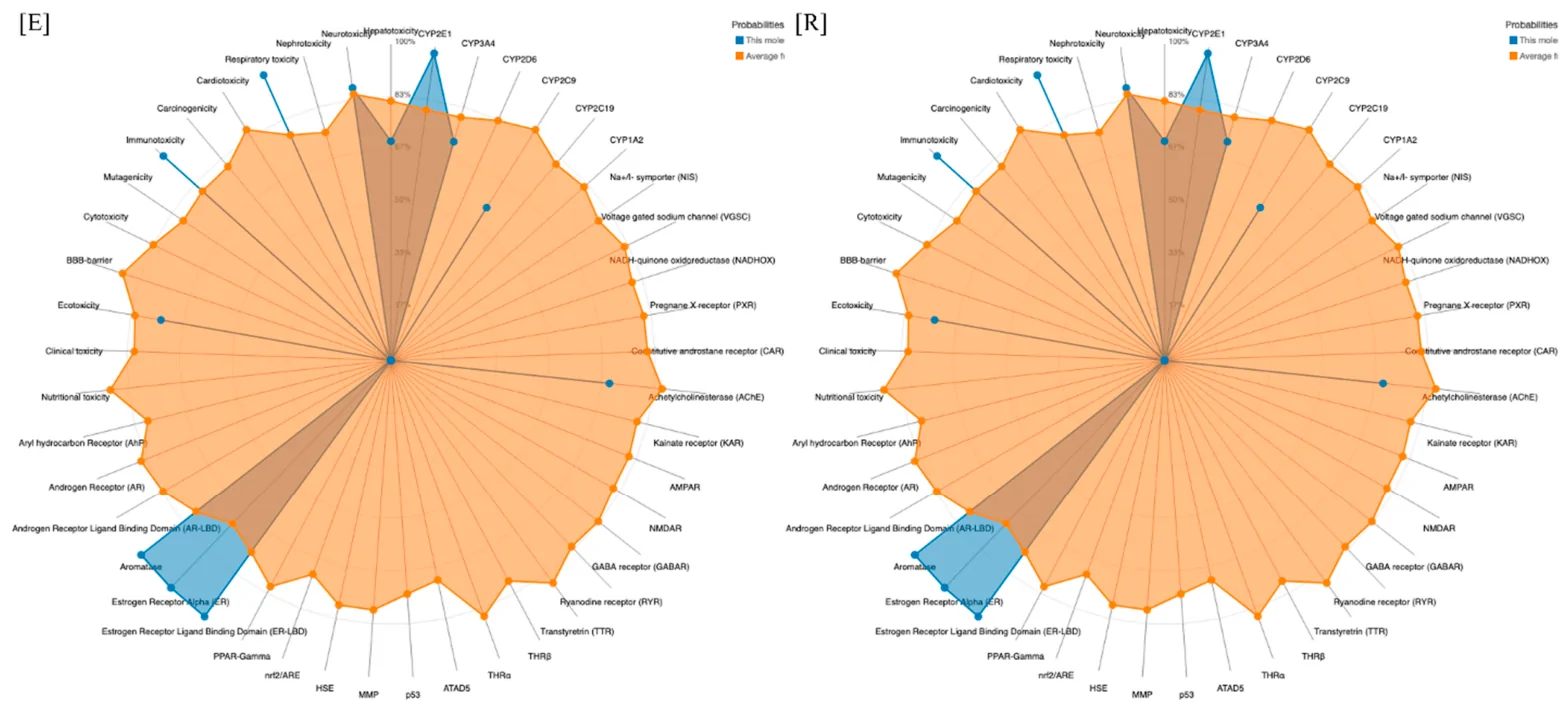
Network-chart-based profile prediction of novel compound **14** [E] and reference drug [R].

**Figure 14:**
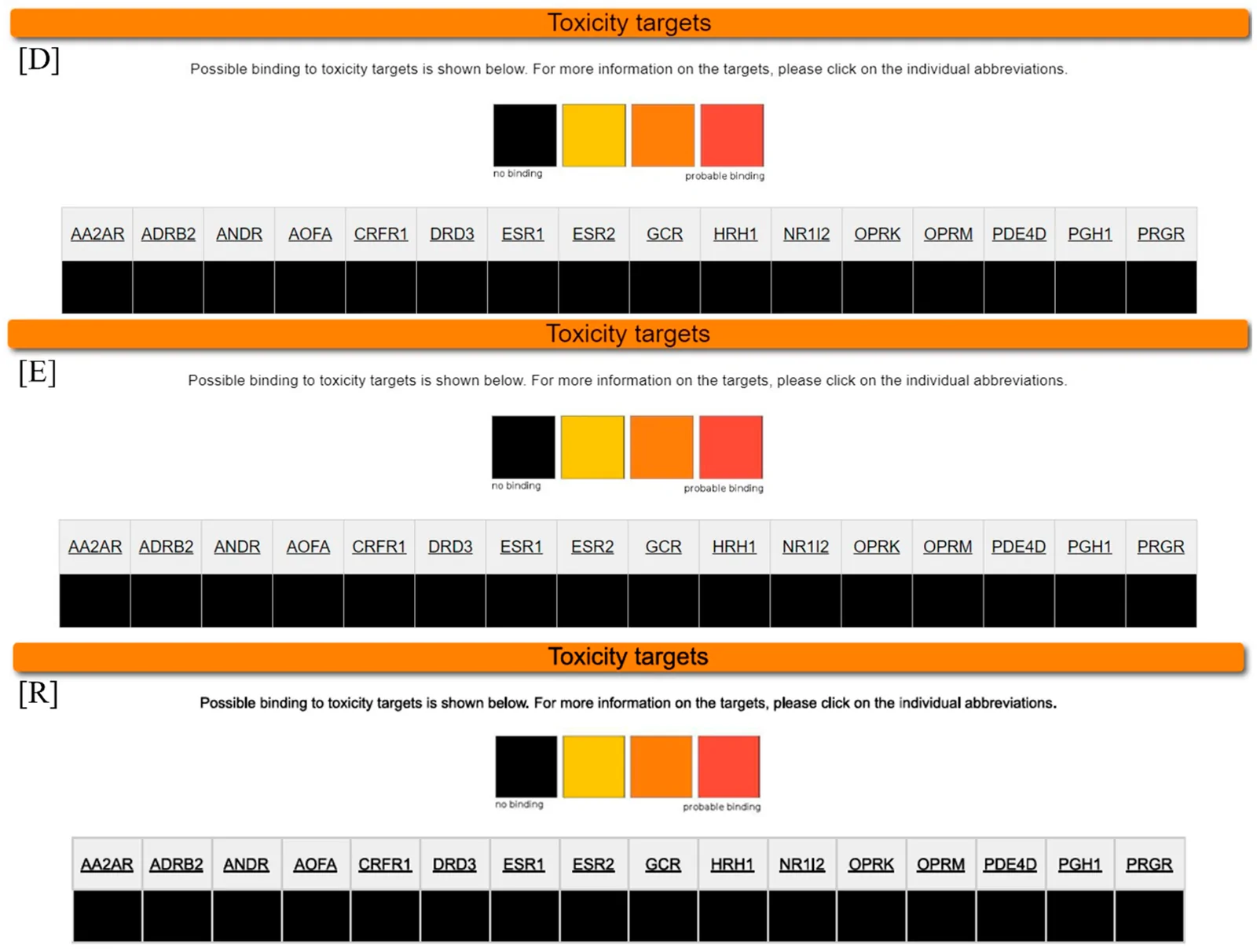
Network-chart-based profile prediction of novel compound **13** [D], compound **14** [E] and reference drug [R].

A notable exception was observed for the Aryl Hydrocarbon Receptor (AhR). The reference inhibitor displayed an inactive AhR interaction prediction, with a probability of 0.81. Similar activity trends were observed for compound **9** (0.58), compound **13** (0.52) and compound **14** (0.69). The activation of AhR-associated pathways may have included cytochrome P450 expression, which potentially alters metabolic clearance and promotes the formation of hepatotoxic reactive metabolites. Compound **14** deviated from this pattern and exhibited an inactive AhR prediction probability (0.69, inactive), which indicates a potentially safer metabolic interaction profile, as summarized in Figure 12, Figure 13 and Figure 14 and the supplementary files (Tables S2 and S3). The predicted absence of mutagenicity for compound **14** may be associated with its distinct substitution pattern, especially the 2-fluoro-6-(pyrrolidin-1-yl)pyridine moiety, together with its relatively balanced physicochemical profiles. The current data do not establish a specific structural determinant that is responsible for this prediction. So, these features should be considered as a preliminary design hypothesis that may be incorporated into related RSK4 active analogues to explore a potential safety advantage followed by experimental mutagenicity validation.

#### 2.4.7. Off-Target Interaction Characterization and Molecular Initiating Events (MIEs)

An investigation of MIEs revealed distinct off-target interaction patterns across the compound series. Particular attention was directed toward Transthyretin (TTR), which transports proteins involved in thyroid hormone homeostasis. Computational analysis predicted active TTR contact for compound **2** (0.55, active), compound **8** (0.52, active), compound **9** (0.54, active) and compound **13** (0.53, active). These results suggest that structural modifications within these derivatives may interfere with physiological thyroid hormone transport mechanisms, as shown in Table 7.

Compound **14** exhibited no predicted TTR interaction probability (0.86, inactive), which closely parallels the reference inhibitor at a probability of 0.81(inactive). Compound **8** alone demonstrated active interaction with thyroid hormone receptor beta (THR-β) at a probability of 0.55(active), whereas all other scaffolds were predicted to be inactive across additional MIE pathways, e.g., NMDA receptor systems, Ryanodine and GABA.

### 2.5. Structure–Activity Relationship (SAR) Implications for Lead Optimization

The current comparative in silico investigation provides important SAR insights that are relevant to future lead optimization strategies.

Compound **14** exhibited a favorable predicted TTR profile, although the structural basis of this difference requires experimental confirmation. The structural incorporation of heterocyclic functionalities successfully eliminated the predicted mutagenicity, TTR-contact liabilities, DILI risk and AhR activation observed in the other derivatives. Although compound **14** demonstrated an improved systemic safety characteristic, respiratory toxicity predictions persisted, and substantial neurotoxicity was likely due to its pronounced BBB permeability. Future structural refinements should focus on precise modulation of molecular polarity and basicity to minimize central-nervous-system (CNS) penetration while maintaining the favorable safety profile identified in the current investigation.

### 2.6. Molecular Dynamic (MD) Simulation Analysis

**Figure 15:**
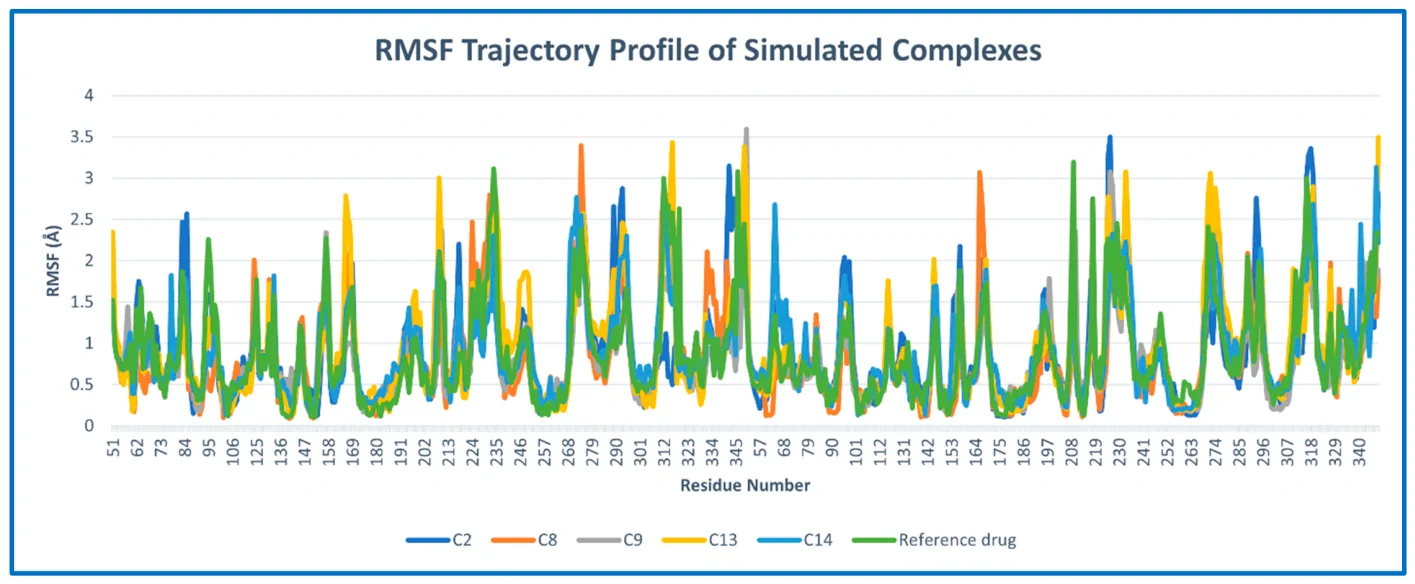
RMSF trajectory profiles of novel compound **2**, compound **8**, compound **9**, compound **13**, compound **14** and reference drug with the target (RSK4). The RMSF values, obtained via CABS-FLEX-3.0, were used to assess dynamic behavior and were calculated for each amino acid residue to evaluate residue levels and conformational stability throughout the simulation. Variations in RMSF values highlight the influence of ligand-induced conformational stabilization and flexibility modulation within the protein active pocket and adjacent structural domains.

**Figure 16:**
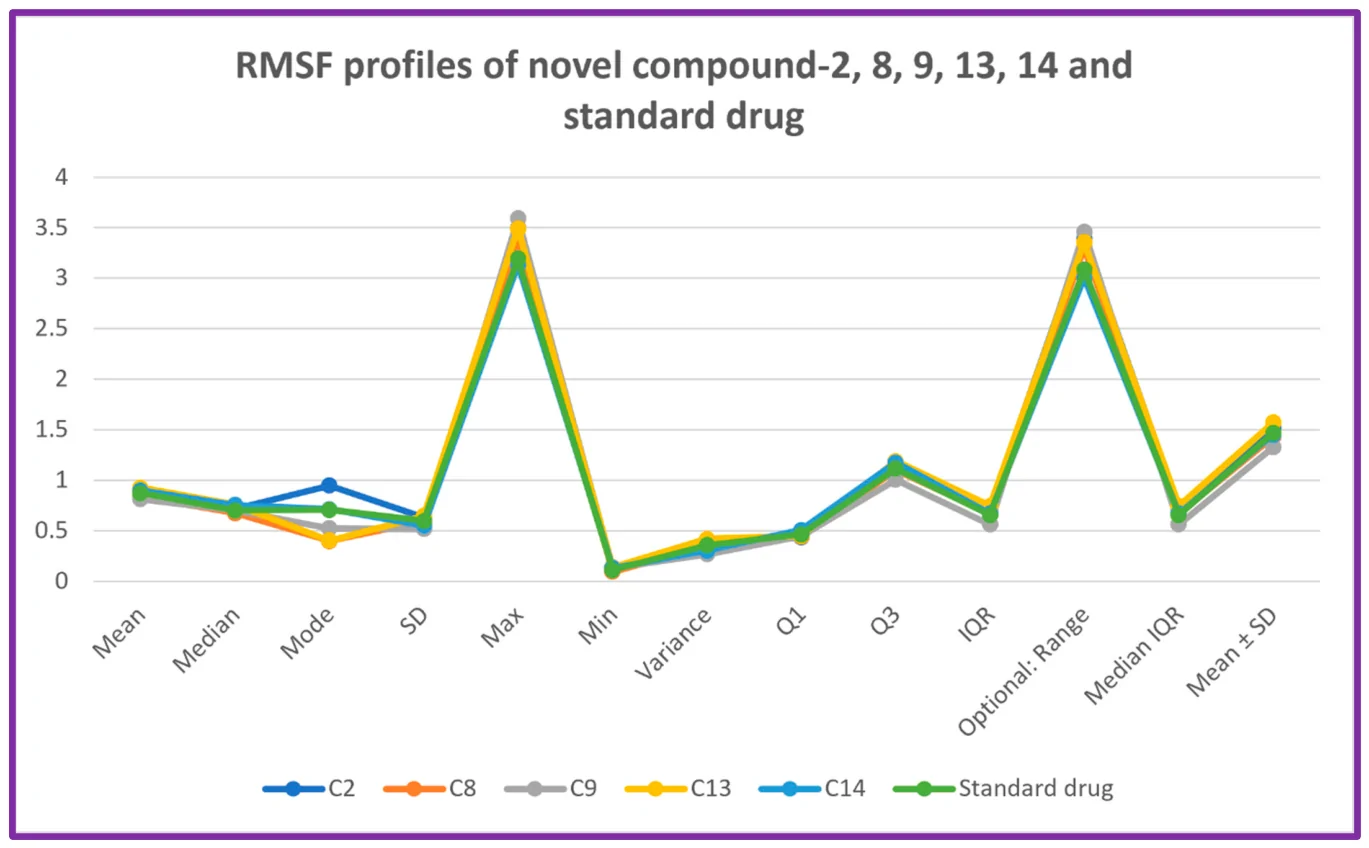
Root-mean-square fluctuation (RMSF) profiles of RSK4 complexed with compounds **2**, **8**, **9**, **13**, and **14** and the standard inhibitor during the molecular dynamics simulations. Key amino acid residues exhibiting notable fluctuations and residues involved in ligand binding are highlighted to illustrate the effect of ligand binding on the conformational flexibility of the RSK4 active site.

MD simulations constitute an advanced computational approach to investigating the intrinsic dynamic behavior of biomolecular systems and elucidate the influence of interactions on protein conformational stability over the simulation time. In the current study, all ligand–target complexes were analyzed via the CABS-flex-3.0 server to evaluate residue-specific flexibility through root-mean-square fluctuation (RMSF) analysis. This strategy provides quantitative insights into residue mobility, molecular recognition, the structural integrity of protein–ligand complexes and conformational adaptability. Higher RMSF values are indicative of increased residue flexibility and conformational freedom, whereas lower RMSF values reflect restricted atomic displacement and enhanced structural rigidity, while the simulation trajectory is as presented in Table 8 and Figure 15 and Figure 16 (supplementary files: Figures S5–S11).

The dynamic behavior induced by the novel series was comparatively evaluated against RSK4. To achieve a comprehensive interpretation of residue-level fluctuations, RMSF datasets were statistically examined via several descriptive parameters, e.g., mean, standard deviation (SD), variance, mode and maximum/minimum values. Quartile-associated descriptors, such as the first quartile (Q1), third quartile (Q3), median, interquartile (IQR) and total fluctuation range, were incorporated to provide a more extensive representation of conformational variability. The integration of these statistical metrics into a unified analytical framework enabled a reliable and systematic comparison of dynamic stability across all simulated complexes.

The obtained results demonstrate that mean and median RMSF values act as reliable global indicators of protein flexibility and effectively describe ligand-mediated modulation of backbone dynamics. Among the synthesized scaffolds compound **8** exhibited the lowest median RMSF value (0.675), whereas compound **9** displayed the lowest median RMSF value (0.698), suggesting reduced conformational mobility and enhanced structural stabilization relative to the standard inhibitor (0.699). The evaluated RMSF values were associated with increased residue fluctuations and greater conformational flexibility. The analysis of mode RMSF described the most frequently sampled fluctuation value as 0.399, indicating the formation of a comparatively stable conformational ensemble, as shown in Table 8.

Further evidence supports the claim that these observations were obtained from dispersion-related statistical descriptors. The standard deviation and variance analysis reflected the distribution and extent of RMSF fluctuations and identified the localized flexible regions within the protein structure. The evaluation of minimum and maximum RMSF values additionally distinguished highly rigid structural domains from flexible loops or terminal regions. Several synthesized scaffolds effectively minimized excessive residue fluctuations in comparison with the reference inhibitor. Compound **9** exhibited particularly favorable minimum (0.133) and maximum (3.592) RMSF values, highlighting its ability to preserve an optimal balance between conformational stability and structural flexibility.

The quartile-based statistical analysis provided additional insights into the central distribution of RMSF values while decreasing the influence of extreme fluctuations. The complexes characterized by narrower interquartile ranges generally exhibited more homogeneous residue flexibility patterns, e.g., improved dynamic consistency upon ligand interaction. The IQR and median values consistently supported the presence of a stabilized fluctuation profile among the most potent complexes. The RMSF range (maximum/minimum) served as an effective indicator of overall structural dispersion where lower values corresponded to tighter dynamic regulation, as was prominently observed for compound **9** (3.459), as summarized in Table 8 (supplementary file: Table S4).

Collectively, the comparative statistical evaluation of CABS-flex-derived RMSF profiles demonstrated distinct ligand-dependent modulation of protein dynamics and highlighted the newly synthesized promising derivatives in stabilizing favorable conformational states. These results further underscore the significance of RMSF-associated statistical descriptors as robust indicators of structural stability and interaction efficiency in protein–ligand systems. The combined interpretation of biological lead candidate identification, molecular dynamics simulations and molecular docking analysis identified compound **9** and compound **14** as the most potent inhibitory candidates. These derivatives remained strong throughout the simulation period, supporting their potential for further structural optimization and future therapeutic development.

A statistical analysis of the RMSF values, summarized in Table 8, revealed that the median RMSF values ranged from 0.675 to 0.7585, suggesting that all investigated scaffolds maintained relatively low conformational fluctuations throughout the MD simulation. The novel compound **8** exhibited the lowest median RMSF values (0.675), indicating the highest overall structural stability of this complex. Compound **13** displayed the highest median value of 0.7585, with slightly greater conformational flexibility, but others were within the acceptable range for stable complexes. The reference inhibitor produced a median RMSF of 0.699, and several novel candidates achieved protein stabilization that was comparable to or better than the standard inhibitor.

An evaluation of the interquartile range (median IQR) further demonstrated the consistency of the residues under simulation. The novel compound **9** showed the lowest median IQR value of 0.56125, which indicates highly uniform structural fluctuations with minimal deviation throughout the simulation trajectory. Compound **13** exhibited the highest median IQR of 0.74325 and showed a broader distribution of residue mobility, while the fluctuations remained within an acceptable range for stable complexes. The reference inhibitor showed an intermediate median IQR value (0.6555), demonstrating moderate conformational variability.

The mean RMSF values show a similar trend, and the novel compound **9** exhibited the lowest average residue fluctuation at 0.810428 ± 0.516153, whereas the compound **13** complex displayed the highest mean RMSF of 0.922878 ± 0.645941. Compound **2** at 0.88755 ± 0.629055, compound **14** (0.899287 ± 0.549692) and the reference inhibitor (0.871350515 ± 0.595577228) demonstrated comparable average flexibility, which suggests that ligand binding did not induce excessive structural perturbation within the receptor. The relatively small standard deviation values indicate stable residue fluctuations throughout the simulation period and support the reliability of the generated conformational ensembles (Table 8).

The CABS-flex 3.0 simulations indicate that all synthesized candidates formed structurally stable complexes. Several of the novel compounds, especially compound **8** and **9**, produced residue-fluctuation characteristics that were equal to or lower than those of standard inhibitors, and these profiles suggest improved stabilization of the receptor–ligand interaction.

The trajectories support the relative conformational stability of the investigated complexes under the simulated conditions. The findings should be interpreted as coarse-grained assessments of conformational behavior rather than definitive evidence of atomistic complex stability. This approach especially may not fully capture detailed side-chain re-arrangements or ligand-specific energetic changes.

The current in silico investigation provides a comprehensive evaluation of the novel synthesized candidates as potential RSK4 inhibitors via integrated complementary computational strategies. Physicochemical and drug-likeness evaluations indicated that the majority of the synthesized derivatives satisfy commonly accepted criteria for the drug-like candidates, and their suitability supports the call for further pharmaceutical advancement. ADMET assessment predicted favorable pharmacokinetic profiles and acceptable toxicity for several drugs. These evaluations represent computational predictions for the experimental confirmation requirement.

Differential immunotoxicity predictions further illustrate the importance of safety-oriented lead optimization, with compounds **2**, **13** and **14** indicating comprehensively favorable computational characteristics, and these predictions require experimental immunotoxicity validation before definitive safety conclusions can be drawn. The promising QED of compound **2** therefore does not negate its predicted mutagenicity liability and predicts the need to incorporate toxicity-aware SAR and the literature on safety screening alongside potency optimization.

Molecular docking demonstrated that the selected scaffolds exhibited favorable predicted affinities toward RSK4 and formed stable interactions. These interaction patterns suggest that the synthesized compounds may interfere with RSK4 activity through complex formation. CABS-flex simulations (RMSF) indicate limited fluctuations in the complexes throughout the simulation. The complementary computational results consistently identify the candidates **8**, **9**, **13** and **14** as the most potent candidates for further biological evaluation. The favorable predicted physicochemical profiles, in silico characteristics, stability of protein interaction patterns and dynamic behaviors collectively support their potential as putative RSK4 inhibitors. These integrated computational analyses consistently support the potential of the synthesized compounds as candidate RSK4 inhibitors, and they should be considered in cellular, biochemical and in vivo studies.

It should be noted that the present study does not experimentally establish selectivity toward RSK4 over other RSK isoforms or protein kinases. The molecular docking results provide preliminary insights into the possible binding mode and interactions of the synthesized compounds with RSK4. Therefore, the identified compounds are referred to as promising RSK4-targeting candidates rather than selective RSK4 inhibitors. Further experimental kinase-panel studies and comparative evaluation against other RSK isoforms will be required to establish their selectivity profile.

These target-level observations should not be interpreted as evidence for cellular anticancer efficacy, so validation in ESCC and non-malignant cell models together with an assessment of proliferation and RSK4 pathway modulation will be required to establish the translational relevance of these leads.

## 3. Materials and Methods

### 3.1. General Information

All solvents and chemical reagents employed in this study were commercially procured and utilized without additional purification unless otherwise specified. Purification of the synthesized compounds was accomplished by silica gel column chromatography (300–400 mesh). The reaction progress was routinely monitored by thin-layer chromatography (TLC) on silica gel F254 plates under UV irradiation. Nuclear magnetic resonance (^1^H and ^13^C-NMR) spectra were recorded on Bruker AVANCE spectrometers operating at 600 and 150 MHz, respectively, using DMSO-*d6* as the solvent. Chemical shifts (δ) were referenced relative to tetramethylsilane (TMS) or residual solvent signals. High-resolution mass spectra (HRMS) were acquired on a Bruker micrOTOF-Q mass spectrometer equipped with an electrospray ionization (ESI) source.

### 3.2. General Procedure for Synthesis of Thiazole-Clubbed Pyrimidine-Linked Hydrazone Derivatives

#### 3.2.1. General Synthetic Procedure for Synthesis of Hydrazide Precursor (**II**)

Methyl 4-aminothiazole-5-carboxylate (**I**) (22 mmol, 4.85 g) was dissolved in excess hydrazine monohydrate (16 mL) in a round-bottom flask fitted with a reflux condenser. The reaction mixture was refluxed for 3–4 h. In this reaction, the conversion of the ester precursor was monitored by thin-layer chromatography (TLC) using n-Hexane/ ethylacetate (7:3, 8:2 *v*/*v*) as the eluent system. After complete disappearance of the starting material, the reaction mixture was allowed to cool to ambient temperature. The resulting precipitate product was isolated by vacuum filtration. The ppt was washed successively with petroleum ether and dried under reduced pressure. The obtained intermediate, a 4-aminothiazole-5-carbohydrazide (**II**) (4.12 g; % yield, 85) precursor, was used in the subsequent synthetic step without additional purification.

#### 3.2.2. General Synthetic Procedure for Synthesis of Thiazole-Clubbed Pyrimidine-Linked Hydrazone Derivatives (**III**)

A mixture of 4-aminothiazole-5-carbohydrazide (**II**) (3 mmol, 1 g for each reaction) and the corresponding substituted aromatic aldehyde (5 mmol, 2.0 equivalent) was prepared in absolute ethanol (30 mL). Catalytic citric acid (15 mol%) in ethanol was subsequently added to facilitate Schiff base condensation. The reaction mixture was heated under reflux for 5 to 6 h. The progress of reaction was periodically monitored through TLC, employing n-Hexane/ethyl acetate (7:3, 8:2 *v*/*v*) as the mobile phase. Upon completion of the reaction, the mixture was cooled to room temperature to induce crystallization of the target products. The precipitated solids were collected by vacuum filtration. The solid precipitate was purified by recrystallization from an ethanol and acetone mixture. Finally, the pure thiazole-clubbed pyrimidine-linked hydrazone product was synthesized at good yields (83–93%).

*(E)-6-((3-chloro-4-hydroxybenzylidene)amino)-5-(3-chloro-4-hydroxyphenyl)-5,6-dihydrothiazolo[4,5-d]pyrimidin-7(4H)-one* (**1**)

Yield: 85%; 2.29 g; color: off-white solid; melting point: 196–197 °C; FTIR (cm^−1^): 3303 (NH), 3380 (CH_aromatic_), 2945 (CH_aliphatic_), 1692 (C=O_amide_), 1611 (C=N_imine_), 1475 (C=C), 1103 (C-S), and 875 (C-Cl); ^1^HNMR (*d6*-DMSO, 600 MHz, ppm) *δ*: 10.05 (s, 2H, -OH), 9.18 (s, 1H, CH_thiazole_), 8.30 (s, 1H, CH_imine_), 7.97 (s, 1H, CH_AR(**A**)_), 7.74 (s, 1H, NH), 7.69 (d, 1H, *J* = 7.2 Hz, H_AR(**A**)_), 7.39 (s, 1H, CH_AR(**B**)_), 7.32 (d, 1H, *J* = 7.6 Hz, H_AR(**B**)_), 7.19 (d, 1H, *J* = 7.8 Hz, H_AR(**A**)_), 7.10 (d, 1H, *J* = 7.5 Hz, H_AR(**B**)_), and 6.04 (s, 1H, CH_pyrimidin_); ^13^C-NMR (*d6*-DMSO, 150 MHz, ppm) *δ*: 162.5, 156.6, 152.4, 149.8, 145.3, 143.6, 140.8, 136.2, 134.7, 132.8, 130.7, 129.6, 126.7, 125.6, 122.8, 120.3, 119.4, 116.3, and 83.9; HRMS *m*/*z* calculated for C_18_H_12_Cl_2_N_4_O_3_S_3_ [M + H]^+^ 435.4532, Found 435.4513.

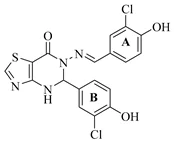

*(E)-6-((4-fluorobenzylidene)amino)-5-(4-fluorophenyl)-5,6-dihydrothiazolo[4,5-d]pyrimidin-7(4H)-one* (**2**)

Yield: 90%; 1.98 g; color: light-green solid; melting point: 196–197 °C; FTIR (cm^−1^): 3321 (NH), 3347 (CH_aromatic_), 3054 (CH_aliphatic_), 1718 (C=O_amide_), 1634 (C=N_imine_), 1456 (C=C), 1127 (C-S), and 1157 (C-F_Ar_); ^1^HNMR (*d6*-DMSO, 600 MHz, ppm) *δ*: 9.61 (s, 1H, CH_thiazole_), 8.79 (s, 1H, CH_imine_), 8.54 (d, 2H, *J* = 8.8 Hz, H_AR(**A**)_), 7.97 (s, 1H, NH), 7.62 (d, 2H, *J* = 8.4 Hz, H_AR(**B**)_), 6.92 (d, 2H, *J* = 7.9 Hz, H_AR(**A**)_), 6.65 (d, 2H, *J* = 7.9 Hz, H_AR(**B**)_), and 6.04 (s, 1H, CH_pyrimidin_); ^13^C-NMR (*d6*-DMSO, 150 MHz, ppm) *δ*: 164.1, 162.4, 155.9, 150.0, 144.2, 142.1, 132.5, 132.1, 128.8, 120.2, 112.8, 111.0, 104.5, 103.5, 100.6, and 84.8; HRMS *m*/*z* calculated for C_18_H_12_F_2_N_4_OS [M + H]^+^ 370.3881, Found 370.3863.

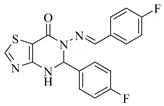

*(E)-6-((4-chlorobenzylidene)amino)-5-(4-chlorophenyl)-5,6-dihydrothiazolo[4,5-d]pyrimidin-7(4H)-one* (**3**)

Yield: 89%; 1.85 g; color: deep-yellow solid; melting point: 205–206 °C; FTIR (cm^−1^): 3361 (NH), 3315 (CH_aromatic_), 2969 (CH_aliphatic_), 1693 (C=O_amide_), 1625 (C=N_imine_), 1454 (C=C), 1124 (C-S), and 846 (C-Cl); ^1^HNMR (*d6*-DMSO, 600 MHz, ppm) *δ*: 9.39 (s, 1H, CH_imine_), 8.88 (s, 1H, CH_thiazole_), 7.99 (d, 2H, *J* = 8.2 Hz, H_AR(**A**)_), 7.59 (s, 1H, NCH_imine_), 7.34 (d, 2H, *J* = 7.8 Hz, H_AR(**B**)_), 7.35 (s, 1H, NH_pyrimidin_), 6.98 (d, 2H, *J* = 7.5 Hz, H_AR(**A**)_), 6.58 (d, 2H, *J* = 8.5 Hz, H_AR(**B**)_), and 5.93 (s, 1H, CH_pyrimidin_); ^13^C-NMR (*d6*-DMSO, 150 MHz, ppm) *δ*: 169.8, 162.1, 159.2, 157.9, 157.6, 146.3, 141.1, 139.6, 133.6, 129.2, 129.1, 127.3, 123.6, 118.7, 110.3, and 84.4; HRMS *m*/*z* calculated for C_18_H_12_Cl_2_N_4_OS [M + H]^+^ 403.4528, Found 403.4511.

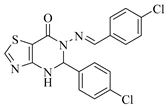

*(E)-6-((4-hydroxy-3,5-dimethoxybenzylidene)amino)-5-(4-hydroxy-3,5-dimethoxyphenyl)-5,6-dihydrothiazolo[4,5-d]pyrimidin-7(4H)-one* (**4**)

Yield: 92%; 2.76 g; color: off-white solid; melting point: 210–211 °C; FTIR (cm^−1^): 3371 (NH), 3418 (CH_aromatic_), 2949 (CH_aliphatic_), 1684 (C=O_amide_), 1624 (C=N_imine_), 1454 (C=C), and 1171 (C-S); ^1^HNMR (*d6*-DMSO, 600 MHz, ppm) *δ*: 10.18 (s, 2H, -OH), 9.27 (s, 1H, CH_thiazole_), 9.04 (s, 1H, CH_imine_), 7.93 (s, 2H, CH_AR(**A**)_), 7.62 (s, 1H, NH), 7.52 (s, 2H, CH_AR(**B**)_), 6.04 (s, 1H, CH_pyrimidin_), and 3.82 (s, 12H, -OCH_3_); ^13^C-NMR (*d6*-DMSO, 150 MHz, ppm) *δ*: 161.9, 157.4, 154.6, 150.3, 147.9, 144.8, 139.7, 136.7, 133.5, 130.3, 130.2, 127.9, 122.6, 116.9, 113.7, 86, and 55.7; HRMS *m*/*z* calculated for C_22_H_22_N_4_O_7_S [M + H]^+^ 486.5239, Found 486.5224.

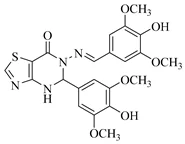

*(E)-6-((4-(dimethylamino)benzylidene)amino)-5-(4-(dimethylamino)phenyl)-5,6-dihydrothiazolo[4,5-d]pyrimidin-7(4H)-one* (**5**)

Yield: 86%; 2.17 g; color: orange solid; melting point: 199–200 °C; FTIR (cm^−1^): 3329 (NH), 3446 (CH_aromatic_), 2974 (CH_aliphatic_), 1719 (C=O_amide_), 1611 (C=N_imine_), 1465 (C=C), and 1121 (C-S); ^1^HNMR (*d6*-DMSO, 600 MHz, ppm) *δ*: 9.26 (s, 1H, CH_thiazole_), 8.96 (s, 1H, CH_imine_), 8.05 (d, 2H, *J* = 8.3 Hz, H_AR(**A**)_), 7.81 (s, 1H, NH), 7.57 (d, 2H, *J* = 7.0 Hz, H_AR(**B**)_), 7.31 (d, 2H, *J* = 7.9 Hz, H_AR(**A**)_), 7.19 (d, 2H, *J* = 8.5 Hz, H_AR(**B**)_), 6.13 (s, 1H, CH_pyrimidin_), and 3.03 (s, 6H, -N(CH_3_)_2_); ^13^C-NMR (*d6*-DMSO, 150 MHz, ppm) *δ*: 164.9, 155.3, 154.1, 152.4, 148.8, 145.4, 142.4, 142.3, 137.9, 133.8, 131.1, 127.9, 124.9, 119.4, 119.2, 113.4, 80.4, and 45.2; HRMS *m*/*z* calculated for C_22_H_24_N_6_OS [M + H]^+^ 420.1276, Found 420.1252.

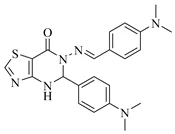

*(E)-6-((4-nitrobenzylidene)amino)-5-(4-nitrophenyl)-5,6-dihydrothiazolo[4,5-d]pyrimidin-7(4H)-one* (**6**)

Yield: 93%; 2.32 g; color: deep-yellow solid; melting point: 210–211 °C; FTIR (cm^−1^): 3353 (NH), 3364 (CH_aromatic_), 2951 (CH_aliphatic_), 1718 (C=O_amide_), 1617 (C=N_imine_), 1441 (C=C), and 1139 (C-S); ^1^HNMR (*d6*-DMSO, 600 MHz, ppm) *δ*: 9.51 (s, 1H, CH_thiazole_), 9.07 (s, 1H, CH_imine_), 8.61 (d, 2H, *J* = 8.2 Hz, H_AR(**A**)_), 7.77 (d, 2H, *J* = 7.2 Hz, H_AR(**B**)_), 7.49 (d, 2H, *J* = 7.4 Hz, H_AR(**A**)_), 7.41 (s, 1H, NH), 6.57 (d, 2H, *J* = 8.4 Hz, H_AR(**B**)_), and 5.97 (s, 1H, CH_pyrimidin_); ^13^C-NMR (*d6*-DMSO, 150 MHz, ppm) *δ*: 165.2, 163.1, 154.7, 153.5, 152.7, 144.3, 143.6, 134.5, 125.1, 123.6, 123.6, 113.5, 110.9, 103.7, and 83.7; HRMS *m*/*z* calculated for C_18_H_12_N_6_O_5_S [M + H]^+^ 424.7467, Found 424.7451.

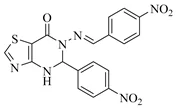

*(E)-6-((3-bromo-4-hydroxybenzylidene)amino)-5-(3-bromo-4-hydroxyphenyl)-5,6-dihydrothiazolo[4,5-d]pyrimidin-7(4H)-one* (**7**)

Yield: 89%; 2.71 g; color: dark-brown solid; melting point: 208–209 °C; FTIR (cm^−1^): 3313 (NH), 3330 (CH_aromatic_), 2920 (CH_aliphatic_), 1673 (C=O_amide_), 1608 (C=N_imine_), 1469 (C=C), and 1113 (C-S); ^1^HNMR (*d6*-DMSO, 600 MHz, ppm) *δ*: 10.13 (s, 2H, -OH), 9.38 (s, 1H, CH_thiazole_), 8.74 (s, 1H, CH_imine_), 8.03 (s, 1H, CH_AR(**A**)_), 7.92 (s, 1H, NH), 7.69 (d, 1H, *J* = 8.2 Hz, H_AR(**A**)_), 7.54 (s, 1H, CH_AR(**B**)_), 7.42 (d, 1H, *J* = 8.3 Hz, H_AR(**B**)_), 7.35 (d, 1H, *J* = 7.4 Hz, H_AR(**A**)_), 7.21 (d, 1H, *J* = 7.5 Hz, H_AR(**B**)_), and 6.12 (s, 1H, CH_pyrimidin_); ^13^C-NMR (*d6*-DMSO, 150 MHz, ppm) *δ*: 164.2, 158.3, 153.5, 148.3, 144.3, 142.2, 138.4, 135.2, 132.5, 130.3, 128.8, 123.0, 120.8, 119.4, 119.2, 115.8, and 84; HRMS *m*/*z* calculated for C_18_H_12_Br_2_N_4_O_3_S [M + H]^+^ 524.8719, Found 524.8703.

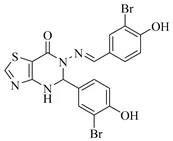

*(E)-6-((2,5-difluoro-4-methoxybenzylidene)amino)-5-(2,5-difluoro-4-methoxyphenyl)-5,6-dihydrothiazolo[4,5-d]pyrimidin-7(4H)-one* (**8**)

Yield: 92%; 2.41 g; color: off-white solid; melting point: 198–199 °C; FTIR (cm^−1^): 3350 (NH), 3347 (CH_aromatic_), 2961 (CH_aliphatic_), 1692 (C=O_amide_), 1610 (C=N_imine_), 1423 (C=C), and 1153 (C-S); ^1^HNMR (*d6*-DMSO, 600 MHz, ppm) *δ*: 9.58 (s, 1H, CH_thiazole_), 8.79 (s, 1H, CH_imine_), 8.03 (s, 1H, CH_AR(**A**)_), 7.63 (s, 1H, CH_AR(**B**)_), 7.40 (s, 1H, NH), 7.32 (s, 1H, CH_AR(**A**)_), 7.26 (s, 1H, CH_AR(**B**)_), 6.08 (s, 1H, CH_pyrimidin_), and 3.75 (s, 6H, -OCH_3_); ^13^C-NMR (*d6*-DMSO, 150 MHz, ppm) *δ*: 163.0, 156.8, 155.5, 152.0, 149.4, 146.8, 141.8, 139.2, 134.9, 132.8, 130.9, 125.1, 123.0, 118.4, 115.6, 84, and 54.9; HRMS *m*/*z* calculated for C_20_H_14_F_4_N_4_O_3_S [M + H]^+^ 466.7129, Found 466.7105.

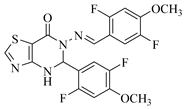

*(E)-6-((3,5-difluoro-4-hydroxybenzylidene)amino)-5-(3,5-difluoro-4-hydroxyphenyl)-5,6-dihydrothiazolo[4,5-d]pyrimidin-7(4H)-one* (**9**)

Yield: 92%; 2.39 g; color: light-green solid; melting point: 209–210 °C; FTIR (cm^−1^): 3407 (NH), 3340 (CH_aromatic_), 2982 (CH_aliphatic_), 1693 (C=O_amide_), 1608 (C=N_imine_), 1430 (C=C), and 1154 (C-S); ^1^HNMR (*d6*-DMSO, 600 MHz, ppm) *δ*: 10.05 (s, 2H, -OH), 9.37 (s, 1H, CH_thiazole_), 8.65 (s, 1H, CH_imine_), 7.99 (s, 2H, CH_AR(**A**)_), 7.48 (s, 1H, NH), 7.32 (s, 2H, CH_AR(**B**)_), and 6.13 (s, 1H, CH_pyrimidin_); ^13^C-NMR (*d6*-DMSO, 150 MHz, ppm) *δ*: 162.4, 155.4, 155.3, 151.9, 146.2, 145.9, 141.2, 140.2, 135.1, 131.9, 130.3, 124.2, 124.1, 120.3, 118.2, and 84; HRMS *m*/*z* calculated for C_18_H_10_F_4_N_4_O_3_S [M + H]^+^ 438.2294, Found 438.2278.

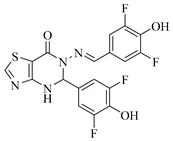

*(E)-6-((3,4,5-trimethoxybenzylidene)amino)-5-(3,4,5-trimethoxyphenyl)-5,6-dihydrothiazolo[4,5-d]pyrimidin-7(4H)-one* (**10**)

Yield: 85%; 1.97 g; color: off-white solid; melting point: 217–218 °C; FTIR (cm^−1^): 3420 (NH), 3293 (CH_aromatic_), 2974 (CH_aliphatic_), 1681 (C=O_amide_), 1627 (C=N_imine_), 1432 (C=C), and 1138 (C-S); ^1^HNMR (*d6*-DMSO, 600 MHz, ppm) *δ*: 9.45 (s, 1H, CH_thiazole_), 8.91 (s, 1H, CH_imine_), 7.92 (s, 2H, CH_AR(**A**)_), 7.59 (s, 2H, CH_AR(**B**)_), 7.29 (s, 1H, NH), 6.06 (s, 1H, CH_pyrimidin_), 3.80 (s, 9H, -OCH_3_), and 3.57 (s, 9H, -OCH_3_); ^13^C-NMR (*d6*-DMSO, 150 MHz, ppm) *δ*: 165.2, 163.4, 162.8, 162.4, 152.4, 145.1, 143.3, 142.8, 139.6, 134.6, 133.2, 131.3, 129.3, 110.2, 106.6, 84.8, 55.6, and 53.9; HRMS *m*/*z* calculated for C_24_H_26_N_4_O_7_S [M + H]^+^ 514.2981, Found 514.2958.

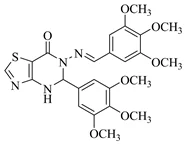

*(E)-6-((4-methylbenzylidene)amino)-5-(p-tolyl)-5,6-dihydrothiazolo[4,5-d]pyrimidin-7(4H)-one* (**11**)

Yield: 83%; 1.36 g; color: off-white solid; melting point: 191–192 °C; FTIR (cm^−1^): 3327 (NH), 3220 (CH_aromatic_), 2943 (CH_aliphatic_), 1681 (C=O_amide_), 1627 (C=N_imine_), 1437 (C=C), and 1121 (C-S); ^1^HNMR (*d6*-DMSO, 600 MHz, ppm) *δ*: 9.28 (s, 1H, CH_thiazole_), 8.75 (s, 1H, CH_imine_), 8.03 (d, 2H, *J* = 8.4 Hz, H_AR(**A**)_), 7.96 (d, 2H, *J* = 7.9 Hz, H_AR(**B**)_), 7.66 (d, 2H, *J* = 7.8 Hz, H_AR(**A**)_), 7.45 (s, 1H, NH), 7.28 (d, 2H, *J* = 8.0 Hz, H_AR(**B**)_), 6.11 (s, 1H, CH_pyrimidin_), and 2.25 (s, 6H, -CH_3_); ^13^C-NMR (*d6*-DMSO, 150 MHz, ppm) *δ*: 162.9, 155.1, 153.3, 153.2, 150.2, 148.1, 140.3, 139.1, 137.4, 134.6, 134.4, 130.2, 126.8, 125.2, 122.9, 118.1, 83.7, and 32.9; HRMS *m*/*z* calculated for C_20_H_18_N_4_OS [M + H]^+^ 362.9452, Found 362.9431.

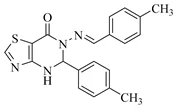

*(E)-6-((4-bromobenzylidene)amino)-5-(4-bromophenyl)-5,6-dihydrothiazolo[4,5-d]pyrimidin-7(4H)-one* (**12**)

Yield: 91%; 2.12 g; color: brown solid; melting point: 221–222 °C; FTIR (cm^−1^): 3326 (NH), 3249 (CH_aromatic_), 2964 (CH_aliphatic_), 1679 (C=O_amide_), 1630 (C=N_imine_), 1430 (C=C), 1128 (C-S), and 682 (C-Br); ^1^HNMR (*d6*-DMSO, 600 MHz, ppm) *δ*: 9.78 (s, 1H, CH_thiazole_), 9.32 (s, 1H, CH_imine_), 8.88 (d, 2H, *J* = 8.0 Hz, H_AR(**A**)_), 8.57 (d, 2H, *J* = 7.4 Hz, H_AR(**B**)_), 7.59 (d, 2H, *J* = 7.6 Hz, H_AR(**A**)_), 7.21 (s, 1H, NH), 6.78 (d, 2H, *J* = 8.3 Hz, H_AR(**B**)_), and 6.01 (s, 1H, CH_pyrimidin_); ^13^C-NMR (*d6*-DMSO, 150 MHz, ppm) *δ*: 164.2, 163.9, 150.1, 149.5, 140.5, 137.2, 135.3, 134.9, 134.7, 131.5, 128.7, 125.7, 112.7, and 82.5; HRMS *m*/*z* calculated for C_18_H_12_Br_2_N_4_OS [M + H]^+^ 489.9150, Found 489.9132.

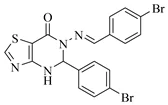

*(E)-6-((4-(trifluoromethoxy)benzylidene)amino)-5-(4-(trifluoromethoxy)phenyl)-5,6-dihydrothiazolo[4,5-d]pyrimidin-7(4H)-one* (**13**)

Yield: 90%; 1.98g; color: off-white solid; melting point: 195–196 °C; FTIR (cm^−1^): 3378 (NH), 3248 (CH_aromatic_), 2940 (CH_aliphatic_), 1677 (C=O_amide_), 1621 (C=N_imine_), 1499 (C=C), 1140 (C-S), and 1264 (Ar-O-CF_3_); ^1^HNMR (*d6*-DMSO, 600 MHz, ppm) *δ*: 9.47 (s, 1H, CH_thiazole_), 8.98 (s, 1H, CH_imine_), 8.11 (d, 2H, *J* = 8.0 Hz, H_AR(**A**)_), 7.90 (d, 2H, *J* = 7.0 Hz, H_AR(**B**)_), 7.86 (d, 2H, *J* = 7.5 Hz, H_AR(**A**)_), 7.36 (s, 1H, NH), 7.17 (d, 2H, *J* = 8.0 Hz, H_AR(**B**)_), and 5.90 (s, 1H, CH_pyrimidin_); ^13^C-NMR (*d6*-DMSO, 150 MHz, ppm) *δ*: 161.3, 154.9, 153.2, 153.1, 149.3, 147.5, 144.9, 140.6, 137.2, 134.5, 131.8, 131.6, 128.2, 124.9, 124.4, 119.7, and 87.3; HRMS *m*/*z* calculated for C_20_H_12_F_6_N_4_O_3_S [M + H]^+^ 502.1987, Found 502.1943.

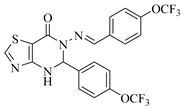

*(E)-5-(2-fluoro-6-(pyrrolidin-1-yl)pyridin-4-yl)-6-(((2-fluoro-6-(pyrrolidin-1-yl)pyridin-4-yl)methylene)amino)-5,6-dihydrothiazolo[4,5-d]pyrimidin-7(4H)-one* (**14**)

Yield: 87%; 1.93 g; color: orange solid; melting point: 223–224 °C; FTIR (cm^−1^): 3341 (NH), 3268 (CH_aromatic_), 2945 (CH_aliphatic_), 1685 (C=O_amide_), 1624 (C=N_imine_), 1430 (C=C), 1140 (C-S), and 1130 (C-F); ^1^HNMR (*d6*-DMSO, 600 MHz, ppm) *δ*: 9.42 (s, 1H, CH_thiazole_), 8.84 (s, 1H, CH_imine_), 8.17 (s, 1H, CH_AR(**A**)_), 8.03 (s, 1H, CH_AR(**B**)_), 7.42 (s, 1H, NH), 7.37 (s, 1H, CH_AR(**A**)_), 7.19 (s, 1H, CH_AR(**B**)_), 6.21 (s, 1H, CH_pyrimidin_), 3.78 (m, 4H, -CH_2 pyrrolidin_), and 2.83 (m, 4H, -CH_2 pyrrolidin_); ^13^C-NMR (*d6*-DMSO, 150 MHz, ppm) *δ*: 164.7, 157.3, 156.2, 153.7, 150.8, 149.2, 147.3, 143.7, 140.2, 137.4, 133.3, 130.2, 127.4, 121.4, 117.9, 83.7, 54.7, and 26.9; HRMS *m*/*z* calculated for C_24_H_24_F_2_N_8_OS [M + H]^+^ 510.8432, Found 510.8411.

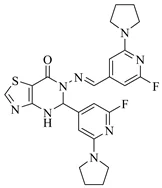



### 3.3. Biochemical RSK4 Kinase Assay Protocol

BI-1870 was employed as the standard inhibitor for all kinase inhibition studies. Prior to biological evaluation BI-D1870 and the synthesized thiazole–pyrimidine hybrid scaffolds were dissolved in di-methyl sulfoxide (DMSO) to prepare stock solutions. An anti-β-tubulin antibody was procured from Tianjin Sungene Biotech. Antibodies against RSK4 were purchased from Signaling Technology, while antibodies against RSK4 were purchased from Proteintech. All other primary and secondary antibodies used in this study were obtained using Cell Counting Kit-8 (CCK-8), which was obtained from Yeasen Biotechnology (Shanghai, China).

#### In-Vitro RSK4 Kinase Inhibition Assay

The inhibitory activity of the synthesized compounds against RSK4 kinase was evaluated via an in-vitro enzymatic assay with minor modifications to previously established protocols. Kinase reactions were carried out in a total volume of 50 µL at 30 °C for 40 min in a reaction buffer containing 40 nM of Tris-HCL (pH 7.4), 10 nM of MgCl_2_, 10 nM of ATP, 0.1 mg/mL of bovine serum albumin (BSA), 0.2 µg/mL of recombinant kinase enzyme, 1 nM of dithiothreitol (DTT) and 100 µM of lipid substrate. Test compounds were dissolved in DMSO and introduced into the reaction mixture as µL aliquots, and a final DMSO concentration of 1% (*v*/*v*) was maintained. Kinase activity was quantified via the Kinase-Glo Plus Luminescence Assay, which determines residual ATP levels after enzymatic turnover. The luminescence signal was directly proportional to the amount of unconsumed ATP and inversely related to kinase catalytic activity [45,46]. All experiments were performed independently in duplicate, and data are reported as the mean ± standard deviation (SD). Half-maximal inhibitory concentration, IC_50_ (nM), values were determined by non-linear regression analysis of concentration response curves through GraphPad Prism 10.1.1. The assay described in this section is a biochemical RSK4 kinase inhibition assay and was performed to determine the direct inhibitory activity of the novel candidates against RSK4.

### 3.4. Molecular Docking, and Ligand and Target Preparation

#### 3.4.1. Ligand Structures Preparation

A novel series was sketched via Chem-Sketch (ACD/Labs, ACD/Labs Toronto, Canada) and subsequently transformed into three-dimensional structures of the selected drugs and then optimized in Discovery Studio Visualizer 2016 (BIOVIA, San Diego, CA, USA). The prepared compounds were then converted into Protein Data Bank (PDB), ID: 6G77 (resolution: 2.50 Å), format for further computational analysis. The reference candidate was retrieved, and its chemical structure was prepared to ensure methodological uniformity throughout the molecular docking investigation. The final prepared ligand structures were saved in PDB format and utilized for molecular docking analyses, and polar hydrogen atoms were added. Native ligands were extracted from the retrieved RSK4 crystal structure and subsequently re-docked via the same molecular docking parameters applied to the synthesized candidates. The co-crystallized (native) ligands are designated as ligand **1** (L1), ligand **2** (L2), ligand **3** (L3) and ligand **4** (L4) throughout the manuscript [47].

#### 3.4.2. Protein Structure Preparation

The three-dimensional crystallographic coordinates of ribosomal S6 kinase 4 (RSK4) were obtained from the Protein Data Bank (PDB), with PDB ID: 6G77 (resolution: 2.50 Å). Structural refinement was performed via Discovery Studio Visualizer 2016 (BIOVIA, San Diego, CA, USA) prior to molecular docking analysis. The preparation protocol involved an elimination of the co-crystallized steric interface and an avoidance of non-specific interactions within the catalytic site. Water molecules and the native ligands were removed. The refined macromolecular structures were subsequently preserved in PDB format for downstream molecular docking studies [48].

#### 3.4.3. Molecular Docking Study

The experimentally reported patent inhibitors of RSK4 were validated via molecular docking protocol because of their documented biological activity and established interactions within the region of the kinase domain. Each patent inhibitor was independently re-docked into the experimentally characterized active site of RSK4. Docked conformations were subsequently compared with the claimed scaffolds to validate the molecular binding affinities. Molecular docking studies were conducted via the integrated AutoDock algorithm implemented in the PyRx (0.8 version, USA, link to download: https://sourceforge.net/projects/pyrx/, accessed on 5 August 2025) virtual platform for the identification of lead candidates. The blind molecular docking strategy was employed, and the grid box was large enough to encompass the RSK4 protein structure. This strategy enabled unrestricted exploration of all potential interacting regions rather than limitations. The macromolecules saved in PDBQT format and chemical structures underwent energy minimization to achieve geometrically stable conformations and enhance molecular docking precision. Grid dimensions were carefully adjusted to encompass all catalytically important residues throughout molecular docking process.

The exhaustiveness parameter was set to 8, and multiple poses were generated for each ligand and conformation, showing the most favorable molecular binding affinities. The top-level binding poses were selected and downloaded for post-docking evaluations.

#### 3.4.4. Post-Molecular Docking Studies

The top-ranked best poses were subjected to comprehensive interaction analysis via Discovery Studio Visualizer 2016 (https://discover.3ds.com/discovery-studio-visualizer-download), which was used to generate detailed two-dimensional (2D) interaction diagrams and three-dimensional (3D) orientation models for a comprehensive interpretation of ligand–target interaction mechanisms. A detailed examination of intermolecular interactions was performed to identify the key factors and stabilization within the RSK4 active site. Hydrogen bonding, pi–alkyl interactions and Van der Waals forces were evaluated as these interactions play critical roles in determining affinity and complex stability [49,50].

### 3.5. ADMET-Lab-3.0

ADMET-lab-3.0 (https://admetlab3.scbdd.com) represents a substantial advancement in computational methodologies for contemporary drug discovery via overcoming the previous limitations associated with endpoint coverage, integrated data analysis and uncertainty quantification. The platform integrates multitask directed messages through neuronal network architectures with molecular descriptors to improve the reliability of ADMET property prediction and robustness. This study explores newly introduced endpoints and a comprehensive framework for pharmacokinetic characteristics and toxicity evaluation. The incorporation of application interface functionality further facilitates high throughput and batch mode analysis for a virtual selection of lead-candidate workflow and computational drug-discovery development pipelines.

Uncertainty estimation module enhances prediction confidence and interpretability throughout in silico analyses and lead optimization studies. Molecular structures were designed and prepared via ChemSketch, and reference candidate compounds were retrieved from the PubChem databases. The overall workflow involved molecular-structure preparation, compound datasets in ADMETlab-3.0 and systematic analysis of the generated prediction outputs [51].

### 3.6. ProTox-3.0 Protocol

The ProTox-3.0 platform offers an intuitive and user-oriented interface for rapid in silico toxicity assessment of chemical entities and can be accessed via https://tox.charite.de. Compounds can be submitted through PubChem identifier or direct molecular structures sketch to enable the toxicity evaluation of both past and hypothetical structures prior to chemical synthesis. The generated outputs include predicted LD values, toxicity classification, structurally related candidates, comparative physicochemical property distributions and prediction confidence scores. ProTox-3.0 provides comprehensive predictions associated with organ toxicity, molecular pathways, toxicity targets, metabolic profiles and toxicological endpoints. The predicted toxicological profiles are additionally represented via toxicity radar charts and network-based visualizations and the results exportable outputs in PDF, images and CSV formats support efficient data interpretation and documentation for computational toxicological drug discovery studies [52].

### 3.7. Molecular Dynamics Simulations

CABS-flex-3.0 (CABS laboratory, Faculty of Chemistry, University of Warsaw, Warsaw, Poland) was utilized to evaluate the conformational behavior and stability of ligand–target complexes within a course-gained molecular dynamic framework. This approach (available at https://lcbio.pl/cabsflex3) is specifically designed to efficiently capture near-native protein flexibility, a balance between accuracy and computational efficiency. Three-dimensional structures of the protein–ligand complexes were submitted in PDB format and subjected to simulations under appropriate parameters, which are calibrated to reproduce backbone dynamics that were comparable to those obtained from all atom molecular dynamics simulations. The CABS-flex methodology employs Monte Carlo samples to generate an ensemble of structurally diverse conformations and represents intrinsic dynamic behavior of the system. Root-mean-square-fluctuation (RMSF) values were calculated on a per-residue basis to quantitatively characterize local flexibility, which enabled an exploration of conformational mobility as well as ligand-induced stabilization of structurally constrained protein segments. This approach enabled a rapid comparative assessment of the prioritized complexes within the current computational workflow. This method employs a coarse-grained representation, and simulations were not intended to reproduce the full atomistic details accessible via conventional all-atom molecular dynamic simulations. Trajectories were analyzed individually and subsequently averaged to minimize stochastic variations and improve confidence in the observed conformational and dynamic trends. The conformational stability and dynamic behavior of the simulated ligand–protein complexes were assessed via the mean, median, mode, standard deviation (SD), max–minimum, variance, quartile 1 (Q1), quartile 3 (Q3), IQR, range, median IQR and mean ± SD [53].

## 4. Conclusions

The present investigation highlights the thiazole–pyrimidine scaffold as a highly potent chemotype for the development of promising RSK4-directed therapeutic agents against esophageal squamous cell carcinoma (ESCC) through a rational drug-design strategy by systematic scaffold refinement and targeted substituent optimization. A structural library was synthesized and characterized through ^1^HNMR, ^13^CNMR and HRMS and subjected to comprehensive biological evaluation against RSK4. Among the tested scaffolds the candidates **2**, **8**, **9**, **13** and **14,** with IC_50_ values of 17.08 ± 1.65 nM, 18.08 ± 0.99 nM, 15.32 ± 1.35 nM, 17.89 ± 1.35 nM, and 21.45 ± 1.69 nM respectively, emerged as the most potent candidates, showing remarkable nanomolar RSK4 inhibitory activity that was comparable to that of the reference inhibitor BI-D1870 at an IC_50_ value of 33.16 ± 1.34 nM. Structure–activity relationship (SAR) analyses revealed that both the electronic nature and positional arrangement of the substituents substantially influenced kinase inhibition and target contact efficiency. Integrated computational investigations, such as molecular docking, with docking scores for scaffolds **2**, **8**, **9**, **13** and **14** that are −9.5, −10.2, −7.8, −9.0 and −10 Kcal/mol compared to the standard drug BI-D1870 at −9.8 Kcal/mol; dynamic simulations; ADMET; and ProTocx-3.0 assessment, demonstrated favorable contact stability, productive ligand–target interactions and potent drug-like pharmacokinetic characteristics, supporting further experimental evaluation for the lead candidates. Similarly, CABS-flex-3.0 simulations supported the relative confirmational stability of the complexes, and longer-time-scale MD simulations will be valuable for further validation in the future. Future studies will focus on evaluating their anticancer efficacy using ESCC cell lines, and in-vivo pharmacological and toxicological assessments to validate their merit as RSK4 inhibitors are required in further investigations. However, these results not only reinforce the therapeutic significance of RSK4 as a viable molecular target in ESCC but also provide a robust mechanistic and structural framework for the future optimization of promising lead candidates targeted at anticancer agents with enhanced selectivity, translational potential and efficacy.

## Figures and Tables

**Table 1:** Different substitution patterns of novel thiazole-clubbed pyrimidine-linked hydrazone conjugates and their in vitro RSK4 kinase analysis.

Sr. No	R Substitution Group	RSK4 Kinase IC_50_ (nM)
1	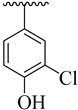	22.87 ± 1.93
2	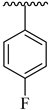	17.08 ± 1.65
3	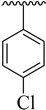	24.13 ± 1.08
4	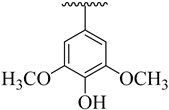	27.25 ± 1.89
5	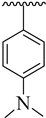	45.70 ± 3.15
6	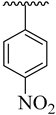	24.48 ± 1.00
7	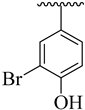	44.34 ± 1.24
8	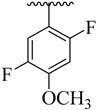	18.08 ± 0.99
9	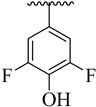	15.32 ± 1.35
10	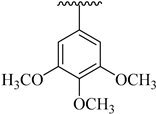	29.48 ± 1.38
11	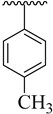	29.10 ± 1.53
12	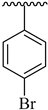	54.61 ± 2.17
13	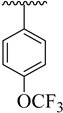	17.89 ± 1.35
14	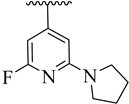	21.45 ± 1.69
**BI-D1870** **Reference Inhibitor**		**33.16 ± 1.34**

**Table 2:** Molecular docking interactions and predicted binding affinity profiles of compounds **2**, **8**, **9**, **13**, and **14** and the reference inhibitor. **L1**–**L4** represent the native ligands extracted from the retrieved RSK4 crystal and used for re-dock validation.

Candidates	Binding Energies, Kcal/mol
2	−9.5
8	−10.2
9	−7.9
13	−9
14	−10
Reference drug (BI-D1870)	−9.8
L1	−7.8
L2	−8
L3	−8.7
L4	−8.4

**Table 3:** Summary of molecular docking interactions and predicted contact profiles of compounds **2**, **8**, **9**, **13**, and **14** and reference inhibitor.

Novel Compounds	Interactions	Distances (Å)
C2	π–alkyl: Leu205, Val87, and Ala103	5.46, 4.50, and 4.53
	π–sigma: Thr215 and Leu79	3.94 and 3.82
	Halogen: Asp153 and Leu155	2.84 and 3.50
C8	Conventional hydrogen bonds: Leu155 and Thr215	2.89 and 3.23
	Carbon–hydrogen bond: Thr215	3.15
	π–alkyl: Lys200, Leu152, Val136, Ala103, and Val87	5.39, 3.46, 3.91, and 4.10
	π-sigma: Leu79 and Leu205	4.40 and 3.71
	Halogen: Gln81 and Asn203	3.24 and 3.04
C9	Conventional hydrogen bonds: Tyr339 and Asp187	3.23 and 2.98
	π–sulfur: His188	5.95
	Halogen: His132, Asp336, and Asp187	3.32, 3.47, and 3.08
	Stacked π-π and T-shaped π-π: His188 and Tyr339	3.71
C13	π –alkyl: Leu205, Val87, Leu155, Ala103, and Leu79	5.10, 4.81, 5.03, 4.40, and 4.36
C14	Conventional hydrogen bonds: Asp159	2.89
	Carbon–hydrogen bond and π–donor hydrogen bonds: Thr215, Leu155, and Glu202	4.15, 2.91, and 2.53
	π–alkyl: Ala103, Leu205, and Leu79	5.25, 5.31, and 3.77
	π–sigma: Val87	4.59
Reference inhibitor (BI-D1870)	Conventional hydrogen bonds: Lys200, Ser83, and Lys221	2.61, 2.59, and 2.93
	π–alkyl: Leu205, Leu79, Leu155, Ala103, Leu152, Lys105, Val136, and Val87	4.63, 4.67, 5.14, 4.41, and 4.53, 4.51, 3.64, and 4.11
	π–sigma: Val87	3.96
	Halogen: Asn203	3.39

**Table 4:** Summary of in silico ADMET prediction.

Drugs	Fsp3	QED	Log P	Log D	VD	BBB
2	0.056	0.709	2.614	2.902	0.217	0.004
8	0.15	0.448	2.544	2.795	0.424	0.0
9	0.056	0.428	2.352	2.564	−0.412	0.0
13	0.15	0.366	3.734	3.291	0.266	0.0
14	0.375	0.41	3.652	3.156	0.173	0.004
Reference inhibitor	0.421	0.76	4.172	3.622	0.213	0.0

**Table 5:** Computational physicochemical profiles of selected novel compounds **2**, **8**, **9**, **13**, and **14** and reference inhibitor to assess their potential safety.

Candidates	Predicted Toxicity Class	Predicted LD_50_ (mg/kg)	Mol Weight	No. of H-Bond Acceptors	No. of H-Bond Donors	No. of Rotatable-Bonds	Molecular Refractivity	TPSA
2	4	1000	370.38	5	1	3	101.82	85.83
8	4	1000	466.41	7	1	5	114.72	104.29
9	4	1000	438.36	7	3	3	105.78	126.29
13	4	1000	502.39	7	1	7	115.27	104.29
14	4	1000	510.56	9	1	5	146.7	118.09
Reference drug (BI-D1870)	4	2000	391.42	7	2	5	109.92	81.59

**Table 6:** Summary of Endpoint specific profile prediction.

Parameters	C2	C8	C9	C13	C14	Reference Inhibitor
Mutagenicity	0.52: Active	0.57: Active	0.54: Active	0.52: Active	0.51: Inactive	0.51: Inactive
Immunotoxicity	0.81: Inactive	0.59: Active	0.62: Active	0.74: Inactive	0.74: Inactive	0.72: Active

**Table 7:** Transthyretin (TTR) interaction profiles.

Parameter	C2	C8	C9	C13	C14	Reference Inhibitor
TTR Interaction	Active	Active	Active	Active	Inactive	Inactive

**Table 8:** Computational molecular dynamic simulation analysis of root-mean-square fluctuation (RMSF) for the selected novel compounds **2**, **8**, **9**, **13**, and **14** and reference inhibitor.

Parameters	C2	C8	C9	C13	C14	Reference Drug
Mean	0.88755	0.838802	0.810428	0.922878	0.899287	0.871350515
Median	0.7155	0.675	0.698	0.7585	0.754	0.699
Mode	0.947	0.399	0.522	0.401	0.712	0.705
SD	0.629055	0.590356	0.516153	0.645941	0.549692	0.595577228
Max	3.5	3.395	3.592	3.492	3.133	3.192
Min	0.111	0.093	0.133	0.137	0.13	0.108
Variance	0.39571	0.348521	0.266414	0.417239	0.302162	0.354712235
Q1	0.433	0.442	0.44475	0.4505	0.5055	0.4625
Q3	1.149	1.096	1.00575	1.189	1.17375	1.11325
IQR	0.72825	0.666	0.56125	0.74325	0.66925	0.6555
Optional: Range	3.389	3.302	3.459	3.355	3.003	3.084
Median IQR	0.72825	0.666	0.56125	0.74325	0.66925	0.6555
Mean ± SD	0.88755 ± 0.629055	0.838802 ± 0.590356	0.810428 ± 0.516153	0.922878 ± 0.645941	0.899287 ± 0.549692	0.871350515 ± 0.595577228
	1.516605	1.429159	1.326581	1.568819	1.448979	1.466927744

## Data Availability

Data is contained within the article and Supplementary Material.

## Supplementary Materials

The following supporting information can be downloaded at: https://www.mdpi.com/article/10.3390/ph19081323/s1, Figure S1: Predicted molecular docking analysis of native ligand **L1** interactions within the catalytic pocket of RSK4 (left). A two-dimensional schematic representation of ligand-protein interaction complex, principal intermolecular contacts to stabilize the complex is presented in right panel. Figure S2: Predicted molecular docking analysis of native ligand **L2** interactions within the catalytic pocket of RSK4, principal intermolecular contacts to stabilize the complex. Figure S3: Predicted molecular docking analysis of native ligand **L3** interactions within the catalytic pocket of RSK4, principal intermolecular contacts to stabilize the complex. Figure S4: Predicted molecular docking analysis of native ligand **L4** interactions within the catalytic pocket of RSK4, principal intermolecular contacts to stabilize the complex. Figure S5: RMSF trajectory profiles of novel compound-**2** with the target (RSK4). Figure S6: RMSF trajectory profiles of novel compound-**8** with the target (RSK4). Figure S7: RMSF trajectory profiles of novel compound-**9** with the target (RSK4). Figure S8: RMSF trajectory profiles of novel compound-**13** with the target (RSK4). Figure S9: RMSF trajectory profiles of novel compound-**14** with the target (RSK4). Figure S10: RMSF trajectory profiles of standard drug with the target (RSK4). Figure S11: RMSF trajectory profiles of novel compound-2, compound-8, compound-9, compound-13, compound-14 and reference drug with the target (RSK4). L2, L2, L3 and L4 represent the native-bound complex ligands. Figure S12: ^1^HNMR for compound-**2**. Figure S13: ^13^CNMR for compound-**2**. Figure S14: FTIR for compound-**2**. Figure S15: HRMS for compound-**2**. Figure S16: ^1^HNMR for compound-**3**. Figure S17: ^13^CNMR for compound-**3**. Figure S18: FTIR for compound-**3.** Figure S19: HRMS for compound-**3.** Figure S20: ^1^HNMR for compound-**6**. Figure S21: ^13^CNMR for compound-**6.** Figure S22: FTIR for compound-**6**. Figure S23: HRMS for compound-**6**. Figure S24: ^1^HNMR for compound-**10**. Figure S25: ^13^CNMR for compound-**10**. Figure S26: FTIR for compound-**10**. Figure S27: HRMS for compound-**10**. Figure S28: ^1^HNMR for compound-**12**. Figure S29: ^13^CNMR for compound-**12**. Figure S30: FTIR for compound-**12**. Figure S31: HRMS for compound-**12**. Figure S32: Bioavailability radar plots of novel compound-**14** [E] and reference drug [R] showing physicochemical profiles. The evaluated physicochemical parameters are consistent with accepted oral drug-likeness requirements and support the suitable pharmacokinetic and bioavailability related profiles. Figure S33: Computational prediction of oral toxicity profiles and toxicity class assignment for novel compound-**2** [A] and compound-**8** [B]. Toxicity assessment provides preliminary insights of the safety profile of the investigated candidates and their prospective suitability for further pharmacological applicability. Figure S34: Computational prediction of oral toxicity profiles and toxicity class assignment for novel compound-**9** [C] and compound-**13** [D]. Toxicity assessment provides preliminary insights of the safety profile of the investigated candidates and their prospective suitability for further pharmacological applicability. Figure S35: Dose-distribution profiles of novel compound-**2** [A], compound-**8** [B], compound-**9** [C], compound-**13** [D], compound-**14** [E] and reference drug [R]. Figure S36: Mol-weight-distribution profiles of novel compound-**2** [A], compound-**8** [B], compound-**9** [C], compound-**13** [D], compound-**14** [E] and reference drug [R]. Figure S37: Bioavailability radar plot for the prediction of drug-likeness and pharmacokinetic profiles of novel compound-**2** [A] and compound-**8** [B]. Figure S38: Bioavailability radar plot for the prediction of drug-likeness and pharmacokinetic profiles of novel compound-**9** [C] and compound-**13** [D]. Figure S39: Network chart-based the profile prediction of novel compound-**2** [A] and compound-**8** [B]. Figure S40: Network chart-based the profile prediction of novel compound-**9** [C] and compound-**13** [D]. Figure S41: Toxicity targets prediction of novel compound-**2** [A], compound-**8** [B] and compound-**9** [C]. Figure S42: Superimposition of the experimentally determined co-crystallized ligand (red) and its re-docked pose (purple) within the RSK4 kinase binding pocket. The close overlap between the two conformations validates the molecular docking protocol and demonstrates its reliability in accurately reproducing the experimentally observed binding orientation. Table S1: Computational Organ Toxicity Prediction of Selected Novel compounds **2**, **8**, **9**, **13**, **14** and standard drug to assess their potential safety and toxicological liabilities. Table S2: Computational Tox-21-Nuclear receptor signaling Prediction of Selected Novel compounds **2**, **8**, **9**, **13**, **14** and standard drug to assess their potential safety and toxicological liabilities. Table S3: Computational Molecular initiating Events Toxicity Prediction of Selected Novel compounds **2**, **8**, **9**, **13**, **14** and standard drug to assess their potential safety and toxicological liabilities. Table S4: Computational Molecular Dynamics Simulations Analysis of Root Mean Square Fluctuation (RMSF) for the Selected Novel compounds **2**, **8**, **9**, **13**, **14** and standard drug. Table S5: Correlate the experimental RSK4 inhibition (IC_50_) with the molecular docking binding energies. S1. Comparison of molecular docking binding energies with the experimental RSK4 inhibitory activities.
