# Supplementary material for: Rational Design of Novel Thiazole-Clubbed Pyrimidine-Linked Hydrazone Conjugates as Promising RSK4 Inhibitors for Esophageal Squamous Cell Carcinoma: Molecular Dynamics Simulations and In Vitro Evaluation
Source: Pharmaceuticals (Basel). 2026 Aug 21;19(8):1323. doi: 10.3390/ph19081323 (PMC13516364; doi:10.3390/ph19081323)
Supplement: Supplementary file 1 [file pharmaceuticals-19-01323-s001.zip › pharmaceuticals-4485916-supplementary.pdf]

**Selective RSK4 Inhibition by Thiazole-Pyrimidine Hydrazones Suppresses Esophageal Squamous Cell Carcinoma Growth**

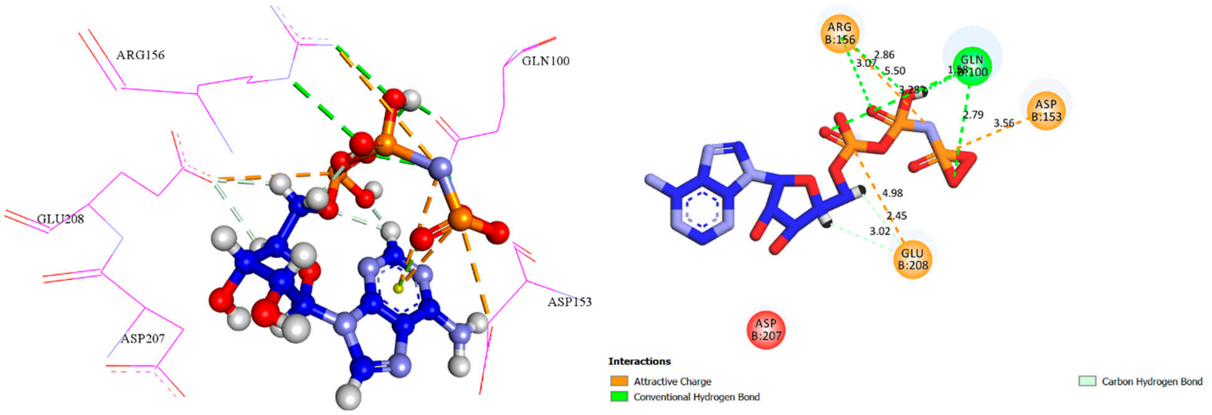


**Figure S1**: Predicted molecular docking analysis of native ligand **L1** interactions within the catalytic pocket of RSK4 (left). A two-dimensional schematic representation of ligand-protein interaction complex, principal intermolecular contacts to stabilize the complex is presented in right panel.

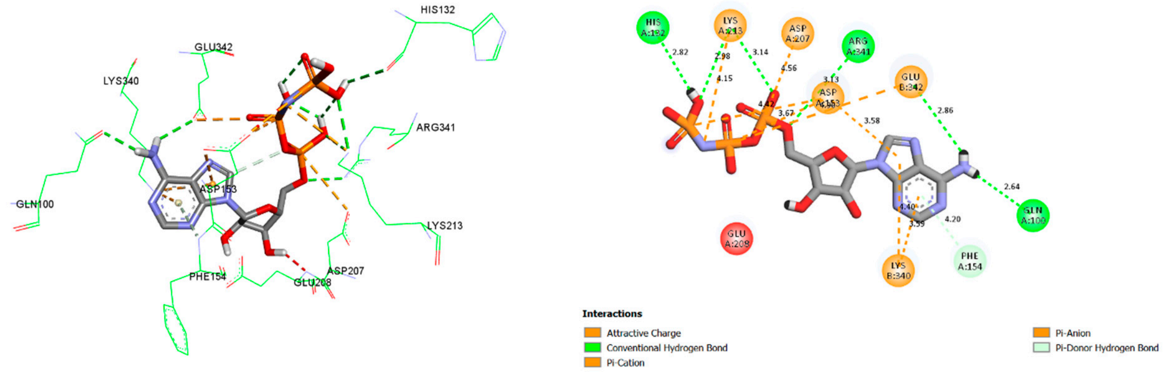


**Figure S2**: Predicted molecular docking analysis of native ligand **L2** interactions within the catalytic pocket of RSK4, principal intermolecular contacts to stabilize the complex.

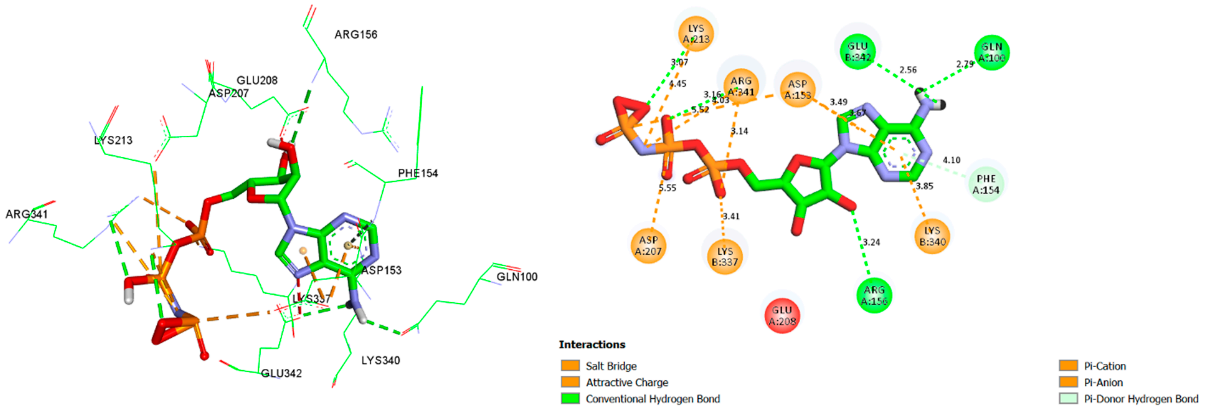


**Figure S3**: Predicted molecular docking analysis of native ligand **L3** interactions within the catalytic pocket of RSK4, principal intermolecular contacts to stabilize the complex.

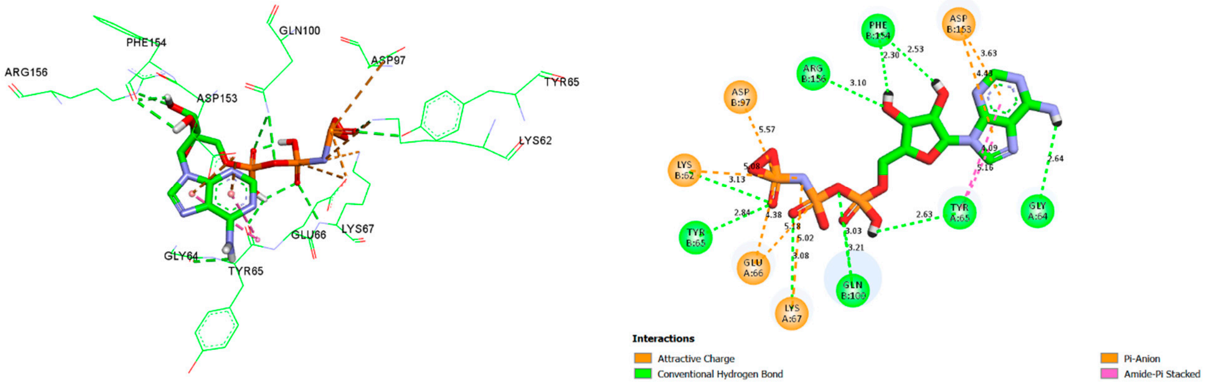


**Figure S4**: Predicted molecular docking analysis of native ligand **L4** interactions within the catalytic pocket of RSK4, principal intermolecular contacts to stabilize the complex.

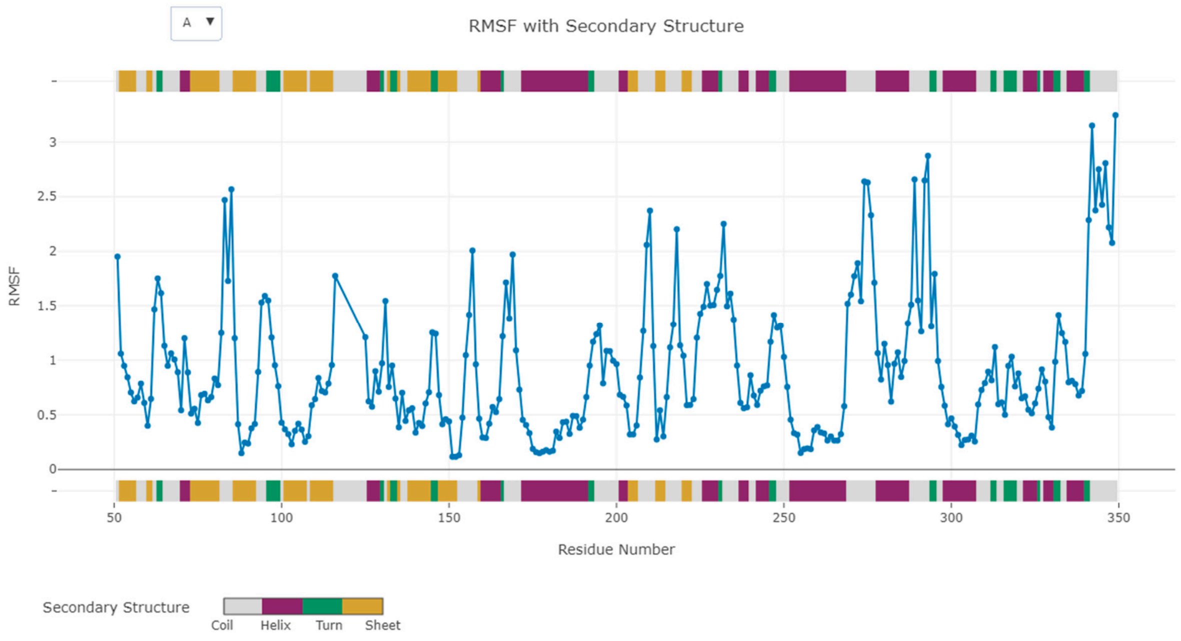


**Figure S5**: RMSF trajectory profiles of novel compound-**2** with the target (RSK4).

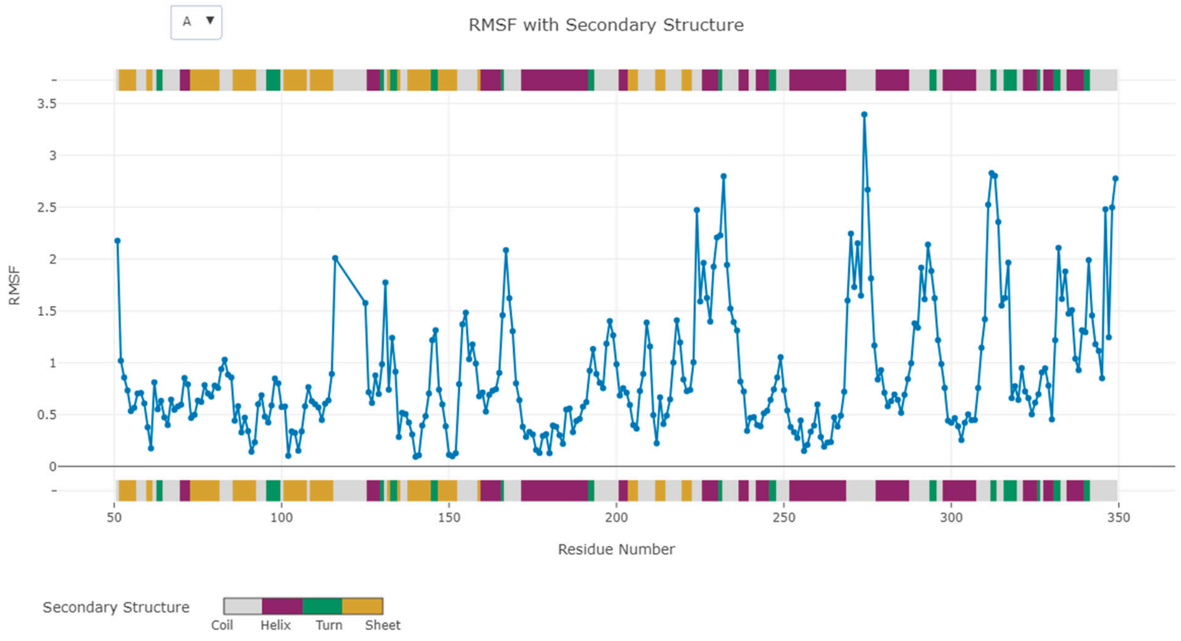


**Figure S6**: RMSF trajectory profiles of novel compound-**8** with the target (RSK4).

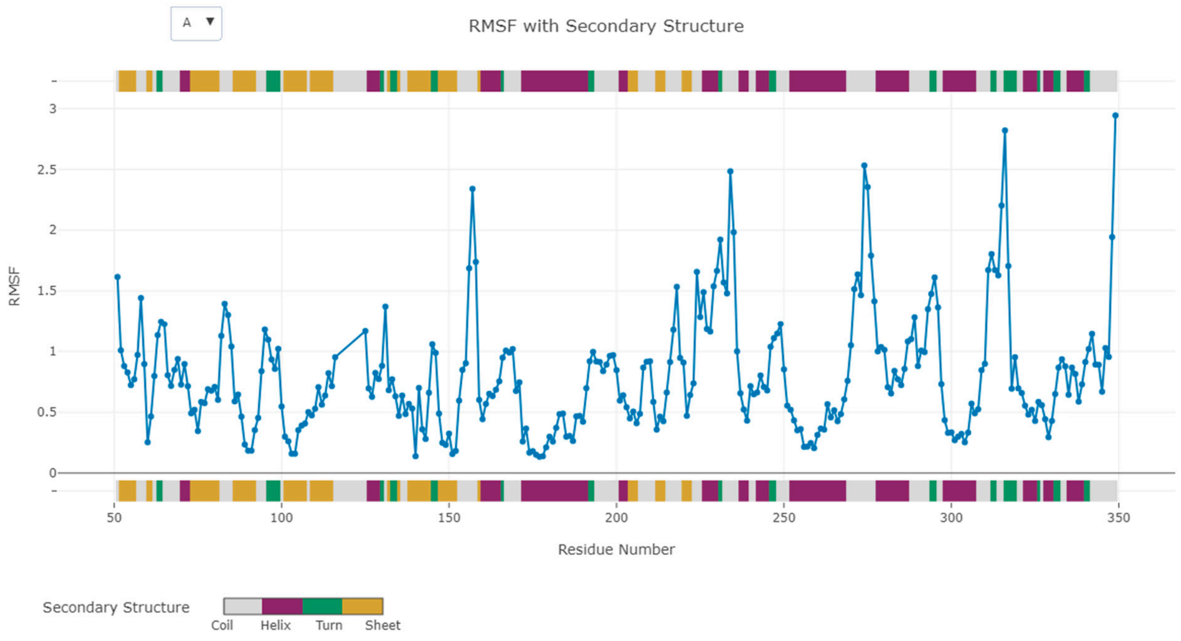


**Figure S7**: RMSF trajectory profiles of novel compound-**9** with the target (RSK4).

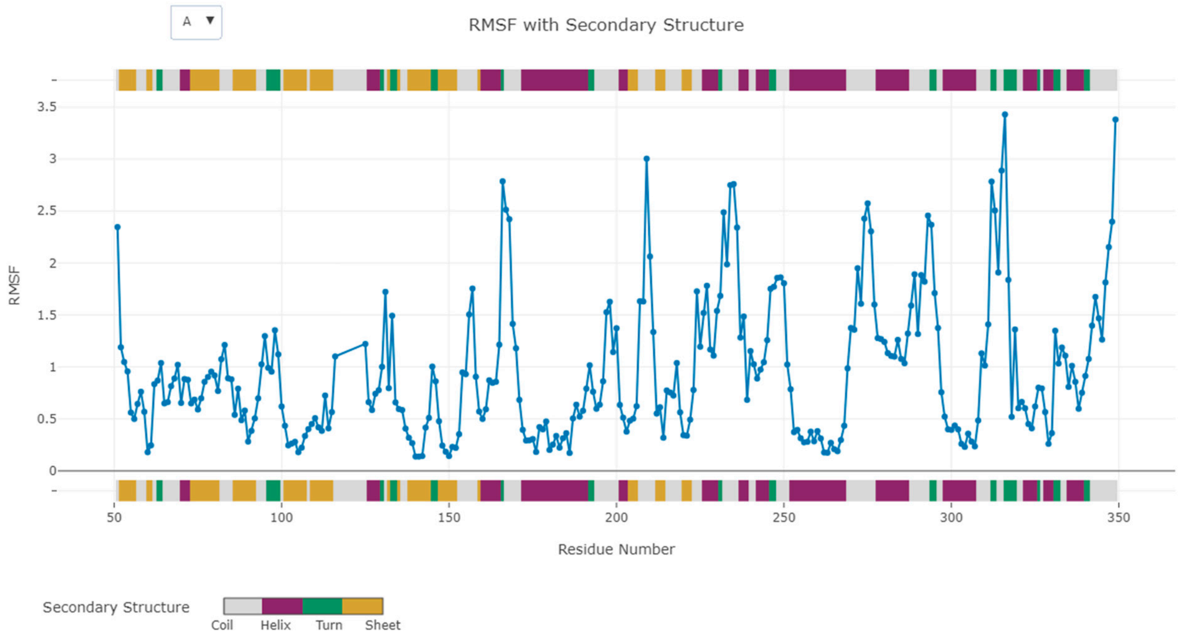


**Figure S8**: RMSF trajectory profiles of novel compound-**13** with the target (RSK4).

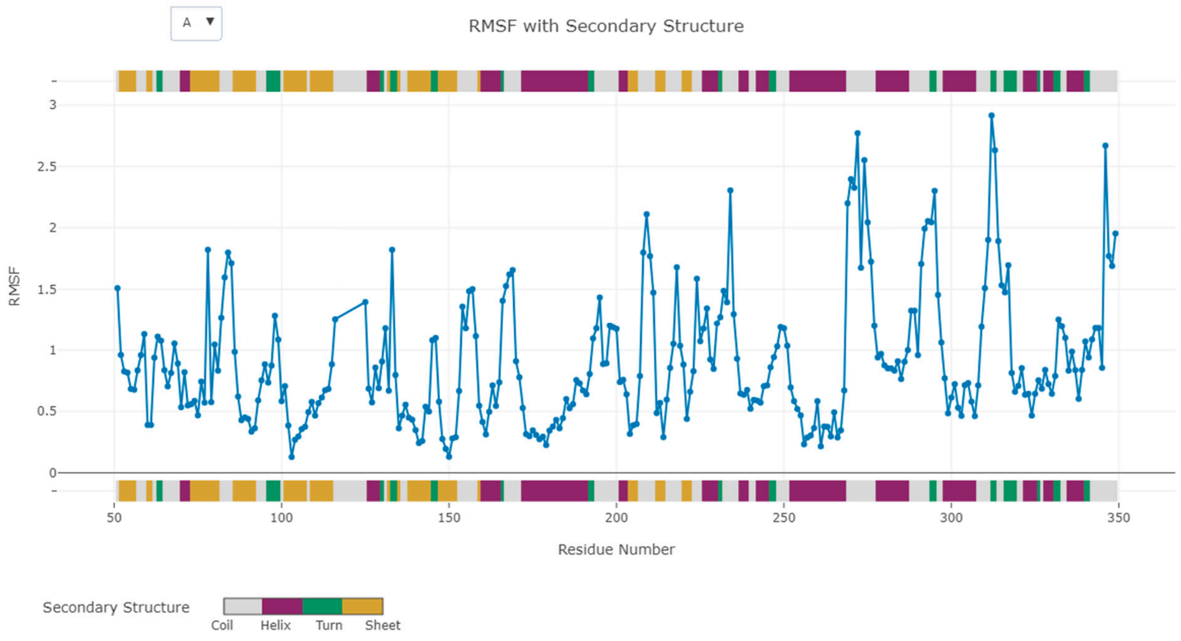


**Figure S9**: RMSF trajectory profiles of novel compound-**14** with the target (RSK4).

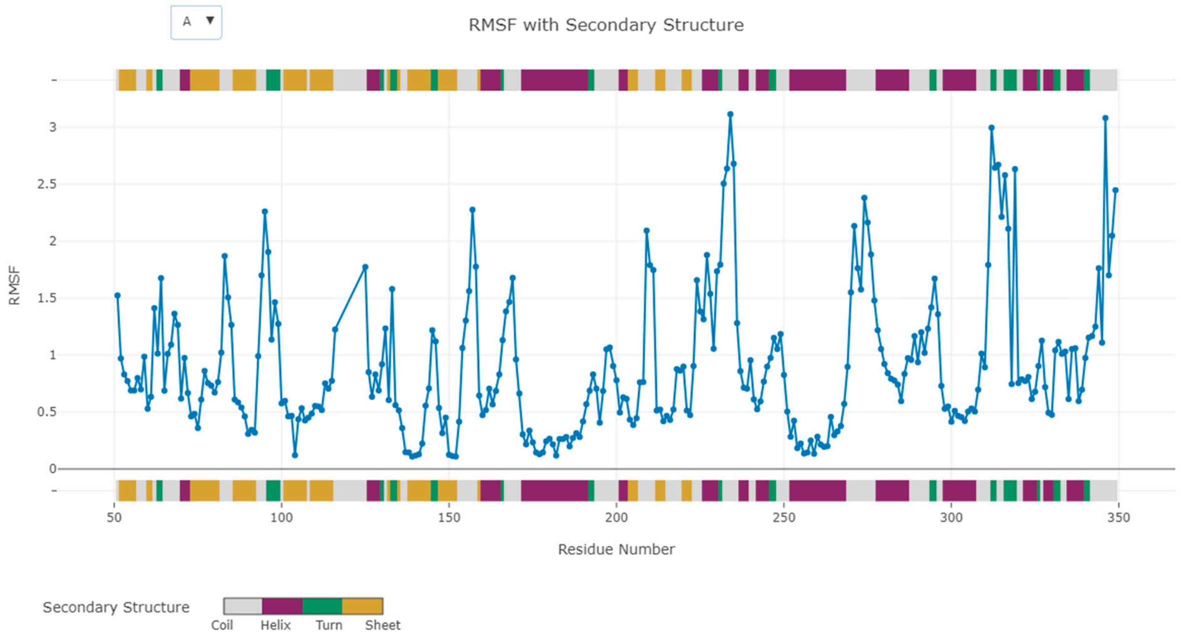


**Figure S10**: RMSF trajectory profiles of standard drug with the target (RSK4).

**Table S1.** Computational Organ Toxicity Prediction of Selected Novel compounds 2, 8, 9, 13, 14 and standard drug to assess their potential safety and toxicological liabilities.

| Candidates | Hepatotoxicity | Neurotoxicity | Nephrotoxicity | Respiratory toxicity | Cardiotoxicity | Carcinogenicity | Immunotoxicity | Mutagenicity | Cytotoxicity | BBB-barrier | Ecotoxicity | Clinical toxicity | Nutritional toxicity |
|---|---|---|---|---|---|---|---|---|---|---|---|---|---|
| | P | P | P | P | P | P | P | P | P | P | P | P | P |
| **2** | 0.61* | 0.74* | 0.65 | 0.58* | 0.88 | 0.54 | 0.81 | 0.52* | 0.80 | 0.89* | 0.60* | 0.52* | 0.70 |
| **8** | 0.60* | 0.65* | 0.62 | 0.50* | 0.79 | 0.63 | 0.59* | 0.57* | 0.79 | 0.71* | 0.55* | 0.51* | 0.65 |
| **9** | 0.63* | 0.66* | 0.53* | 0.57* | 0.82 | 0.59 | 0.62* | 0.54* | 0.76 | 0.62* | 0.59 | 0.54 | 0.68 |
| **13** | 0.63* | 0.67* | 0.61 | 0.54* | 0.77 | 0.55 | 0.74 | 0.52* | 0.78 | 0.75* | 0.59* | 0.53* | 0.52 |
| **14** | 0.60 | 0.86* | 0.74 | 0.93* | 0.87 | 0.59 | 0.74 | 0.51 | 0.67 | 0.93* | 0.60* | 0.56* | 0.72 |
| **Reference drug** | 0.57 | 0.85* | 0.67 | 0.89* | 0.86 | 0.60 | 0.72* | 0.51 | 0.60 | 0.59* | 0.54* | 0.67* | 0.71 |

*=active; P=Probability.

**Table S2.** Computational Tox-21-Nuclear receptor signaling Prediction of Selected Novel compounds 2, 8, 9, 13, 14 and standard drug to assess their potential safety and toxicological liabilities.

| Candidates | Aryl hydrocarbon receptor | Androgen receptor | Androgen receptor ligand binding domain | Aromatase | Estrogen Receptor alpha | Estrogen Receptor ligand binding domain | Peroxisome proliferator activated receptor gamma | Nuclear factor-like 2/antioxidant responsive element | Heat shock factor response element | Mitochondrial membrane potential | Phosphoprotein (p53) | ATPase family AA domain containing protein 5 |
|---|---|---|---|---|---|---|---|---|---|---|---|---|
| | P | P | P | P | P | P | P | P | P | P | P | P |
| **2** | 0.72* | 0.96 | 0.99 | 0.87 | 0.75 | 0.95 | 0.90 | 0.94 | 0.94 | 0.58 | 0.90 | 0.69 |
| **8** | 0.57* | 0.96 | 0.99 | 0.85 | 0.71 | 0.96 | 0.90 | 0.94 | 0.94 | 0.59 | 0.88 | 0.73 |
| **9** | 0.68* | 0.95 | 0.98 | 0.87 | 0.73 | 0.94 | 0.84 | 0.91 | 0.91 | 0.55 | 0.81 | 0.68 |
| **13** | 0.52* | 0.96 | 0.99 | 0.84 | 0.75 | 0.96 | 0.85 | 0.92 | 0.92 | 0.50* | 0.84 | 0.72 |
| **14** | 0.69 | 0.97 | 0.98 | 0.92 | 0.87 | 0.95 | 0.93 | 0.95 | 0.95 | 0.71 | 0.92 | 0.86 |
| **Reference drug** | 0.81 | 0.94 | 0.96 | 0.93 | 0.85 | 0.94 | 0.94 | 0.96 | 0.96 | 0.76 | 0.82 | 0.85 |

*=active; P=Probability.

**Table S3.** Computational Molecular initiating Events Toxicity Prediction of Selected Novel compounds 2, 8, 9, 13, 14 and standard drug to assess their potential safety and toxicological liabilities.

| Candidates | Thyroid hormone receptor alpha | Thyroid hormone receptor beta | Transtyretin | Ryanodine receptor | GABA-receptor | NMDA | AMPAR | Kainate receptor | AChE | CYP1A2 | CYP2C19 | CYP2C9 | CYP2D6 | CYP3A4 | CYP2E1 |
|---|---|---|---|---|---|---|---|---|---|---|---|---|---|---|---|
| | P | P | P | P | P | P | P | P | P | P | P | P | P | P | P |
| **2** | 0.94 | 0.71 | 0.55* | 0.90 | 0.71 | 0.99 | 0.96 | 0.99 | 0.91 | 0.61 | 0.60 | 0.57 | 0.72 | 0.77 | 0.99 |
| **8** | 0.88 | 0.55* | 0.52* | 0.84 | 0.78 | 0.99 | 1.0 | 0.99 | 0.9 | 0.72 | 0.80 | 0.60 | 0.81 | 0.63 | 1.0 |
| **9** | 0.83 | 0.69 | 0.54A | 0.87 | 0.79 | 0.93 | 0.88 | 0.99 | 0.94 | 0.75 | 0.75 | 0.56 | 0.76 | 0.81 | 1.0 |
| **13** | 0.91 | 0.63 | 0.53* | 0.85 | 0.80 | 0.98 | 0.97 | 0.99 | 0.91 | 0.62 | 0.76 | 0.50 | 0.82 | 0.75 | 1.0 |
| **14** | 0.94 | 0.75 | 0.86 | 0.74 | 0.69 | 0.98 | 0.97 | 0.99 | 0.76 | 0.67 | 0.63 | 0.57 | 0.55 | 0.73 | 1.0 |
| **Reference drug** | 0.86 | 0.62 | 0.81 | 0.84 | 0.63 | 0.93 | 0.98 | 1.0 | 0.75 | 0.72 | 0.73 | 0.60 | 0.70 | 0.58 | 0.99 |

*=active; P=Probability.

**Table S4**: Computational Molecular Dynamics Simulations Analysis of Root Mean Square Fluctuation (RMSF) for the Selected Novel compounds 2, 8, 9, 13, 14 and standard drug.

| **Parameters** | **C2** | **C8** | **C9** | **C13** | **C14** | **Reference drug** | **L1** | **L2** | **L3** | **L4** |
|---|---|---|---|---|---|---|---|---|---|---|
| **Mean** | 0.88755 | 0.838802 | 0.810428 | 0.922878 | 0.899287 | 0.871350515 | 0.87438 | 0.867297 | 0.770469 | 0.847148 |
| **Median** | 0.7155 | 0.675 | 0.698 | 0.7585 | 0.754 | 0.699 | 0.7055 | 0.672 | 0.632 | 0.657 |
| **Mode** | 0.947 | 0.399 | 0.522 | 0.401 | 0.712 | 0.705 | 0.537 | 0.592 | 0.727 | 0.637 |
| **SD** | 0.629055 | 0.590356 | 0.516153 | 0.645941 | 0.549692 | 0.595577228 | 0.625136 | 0.66877 | 0.541544 | 0.663615 |
| **Max** | 3.5 | 3.395 | 3.592 | 3.492 | 3.133 | 3.192 | 4.61 | 4.622 | 3.192 | 3.636 |
| **Min** | 0.111 | 0.093 | 0.133 | 0.137 | 0.13 | 0.108 | 0.132 | 0.087 | 0.112 | 0.105 |
| **Variance** | 0.39571 | 0.348521 | 0.266414 | 0.417239 | 0.302162 | 0.354712235 | 0.390795 | 0.447253 | 0.29327 | 0.440385 |

| | | | | | | | | | | |
|---|---|---|---|---|---|---|---|---|---|---|
| **Q1** | 0.433 | 0.442 | 0.44475 | 0.4505 | 0.5055 | 0.4625 | 0.453 | 0.42325 | 0.39925 | 0.39725 |
| **Q3** | 1.149 | 1.096 | 1.00575 | 1.189 | 1.17375 | 1.11325 | 1.11875 | 1.0875 | 1.017 | 1.0375 |
| **IQR** | 0.72825 | 0.666 | 0.56125 | 0.74325 | 0.66925 | 0.6555 | 0.66825 | 0.66675 | 0.61975 | 0.6415 |
| **Optional: Range** | 3.389 | 3.302 | 3.459 | 3.355 | 3.003 | 3.084 | 4.478 | 4.535 | 3.08 | 3.531 |
| **Median IQR** | 0.72825 | 0.666 | 0.56125 | 0.74325 | 0.66925 | 0.6555 | 0.66825 | 0.66675 | 0.61975 | 0.6415 |
| **Mean ± SD** | 0.88755 ± 0.629055 | 0.838802 ± 0.590356 | 0.810428 ± 0.516153 | 0.922878 ± 0.645941 | 0.899287 ± 0.549692 | 0.871350515 ± 0.595577228 | 0.87438 ± 0.625136 | 0.867297 ± 0.66877 | 0.770469 ± 0.541544 | 0.847148 ± 0.663615 |
| | 1.516605 | 1.429159 | 1.326581 | 1.568819 | 1.448979 | 1.466927744 | 1.499515 | 1.536067 | 1.312013 | 1.510763 |

L2, L2, L3 and L4: native ligands

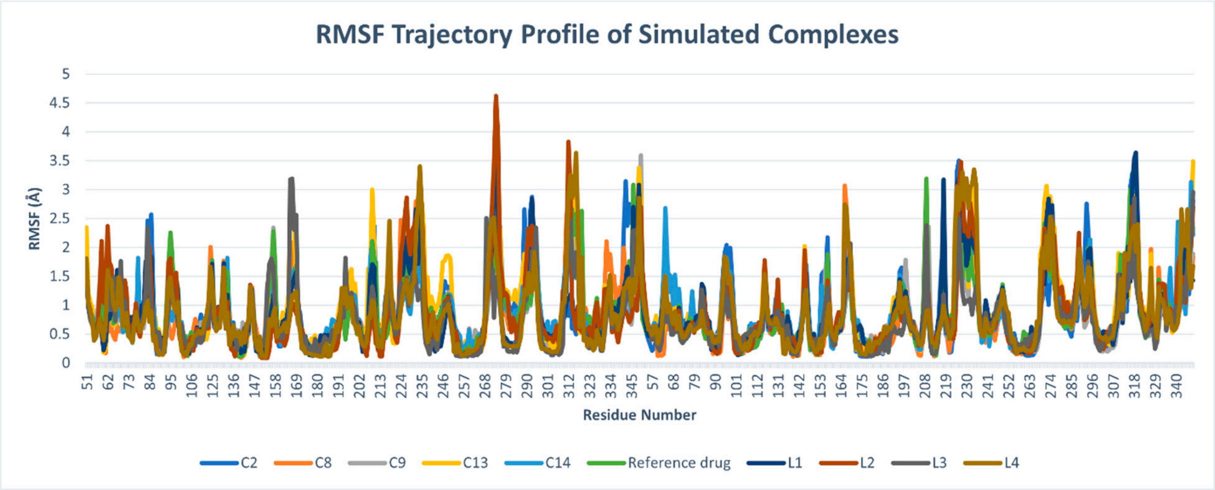


**Figure S11**: RMSF trajectory profiles of novel compound-2, compound-8, compound-9, compound-13, compound-14 and reference drug with the target (RSK4). L2, L2, L3 and L4 represent the native-bound complex ligands.

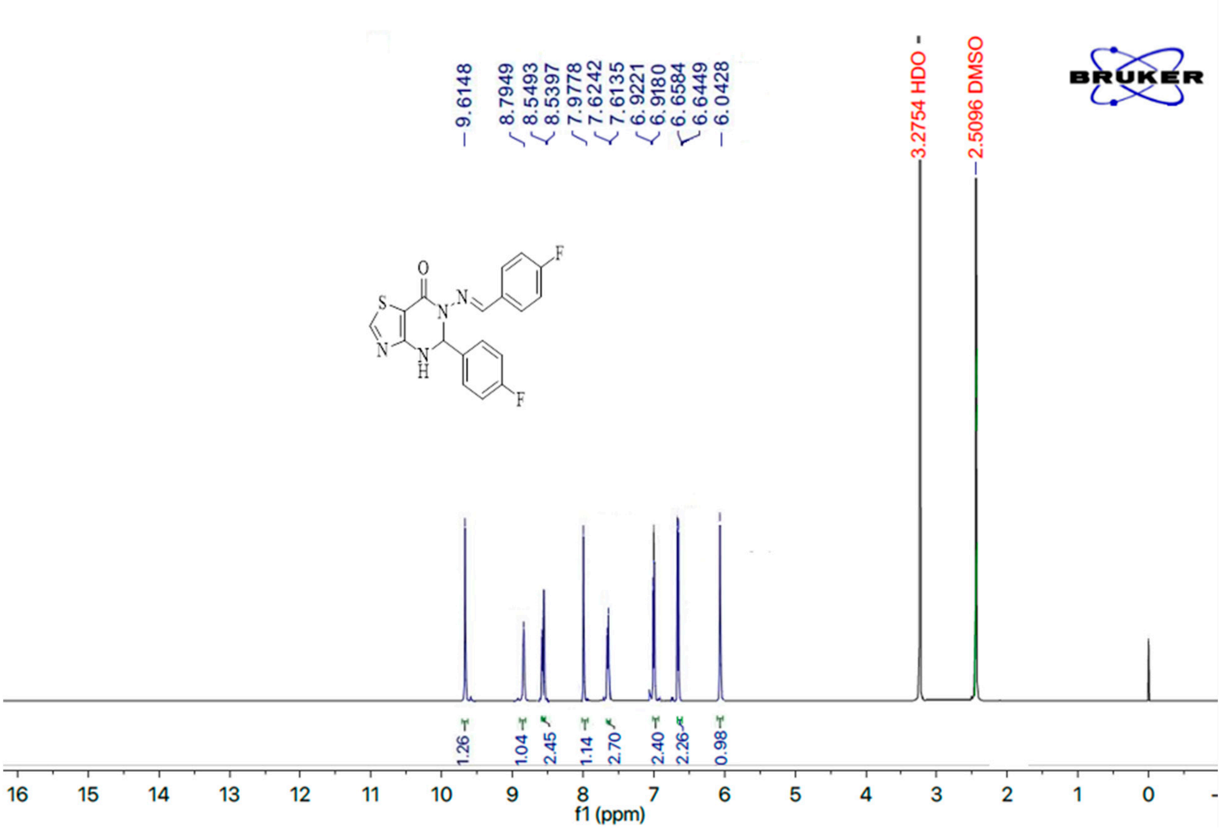


**Figure S12:** $^{1}$HNMR for compound-**2**

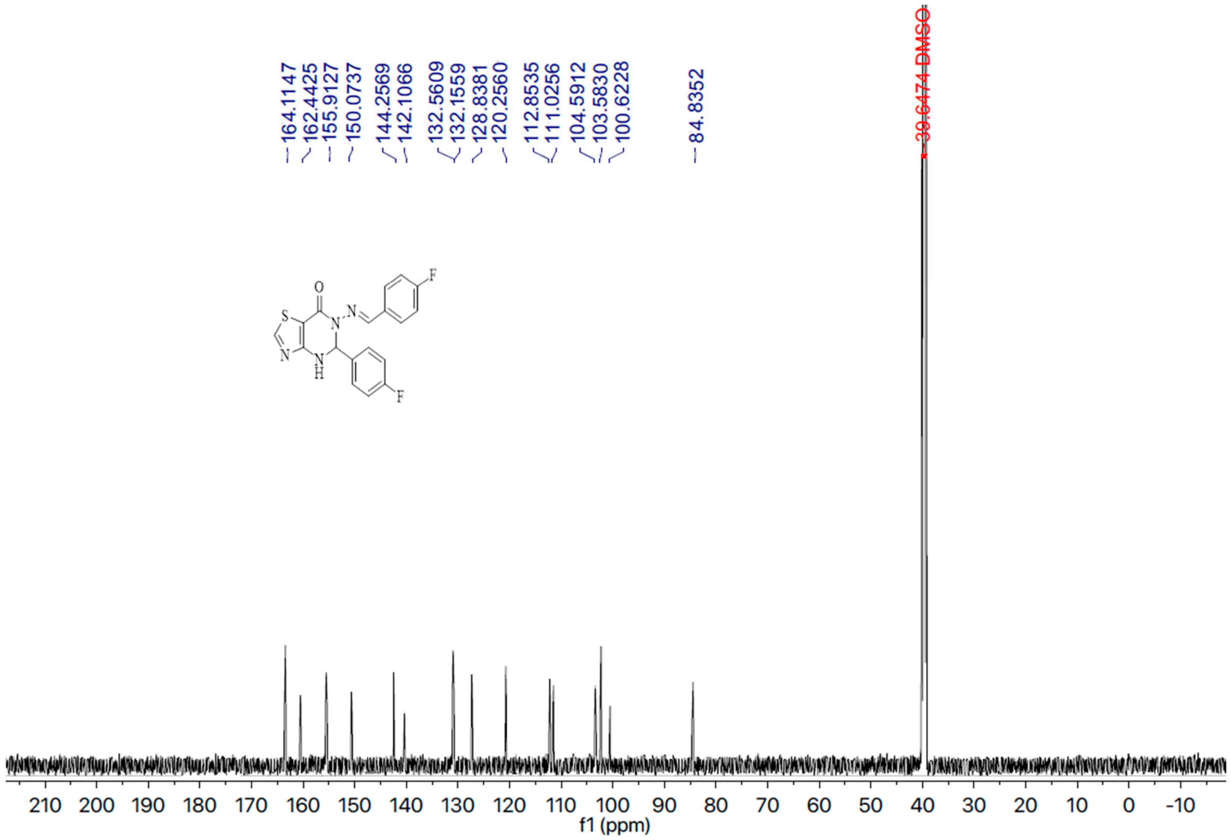


**Figure S13:** $^{13}$CNMR for compound-**2**

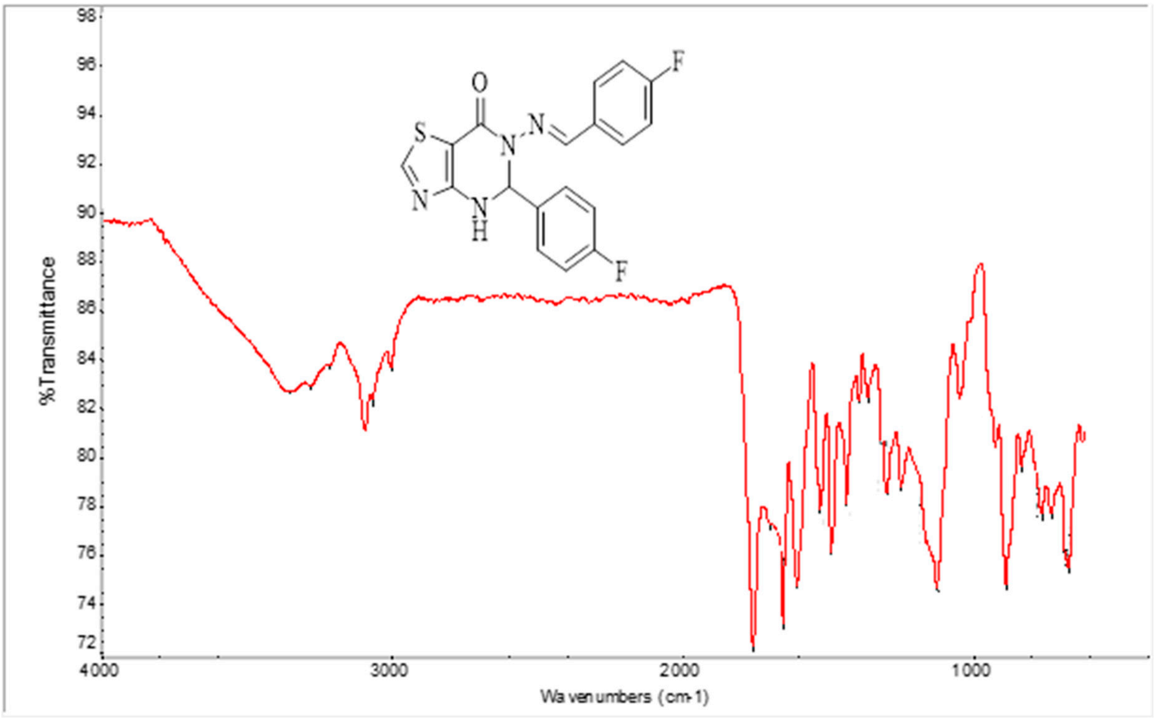


**Figure S14:** FTIR for compound-**2**

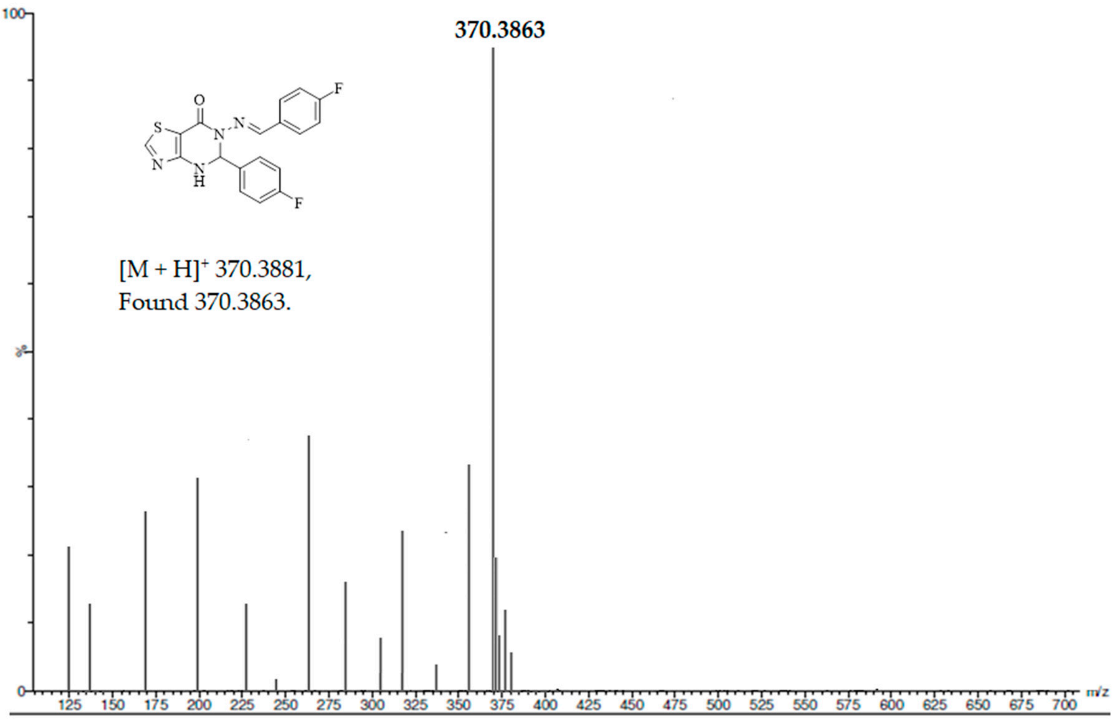


**Figure S15:** HRMS for compound-**2**

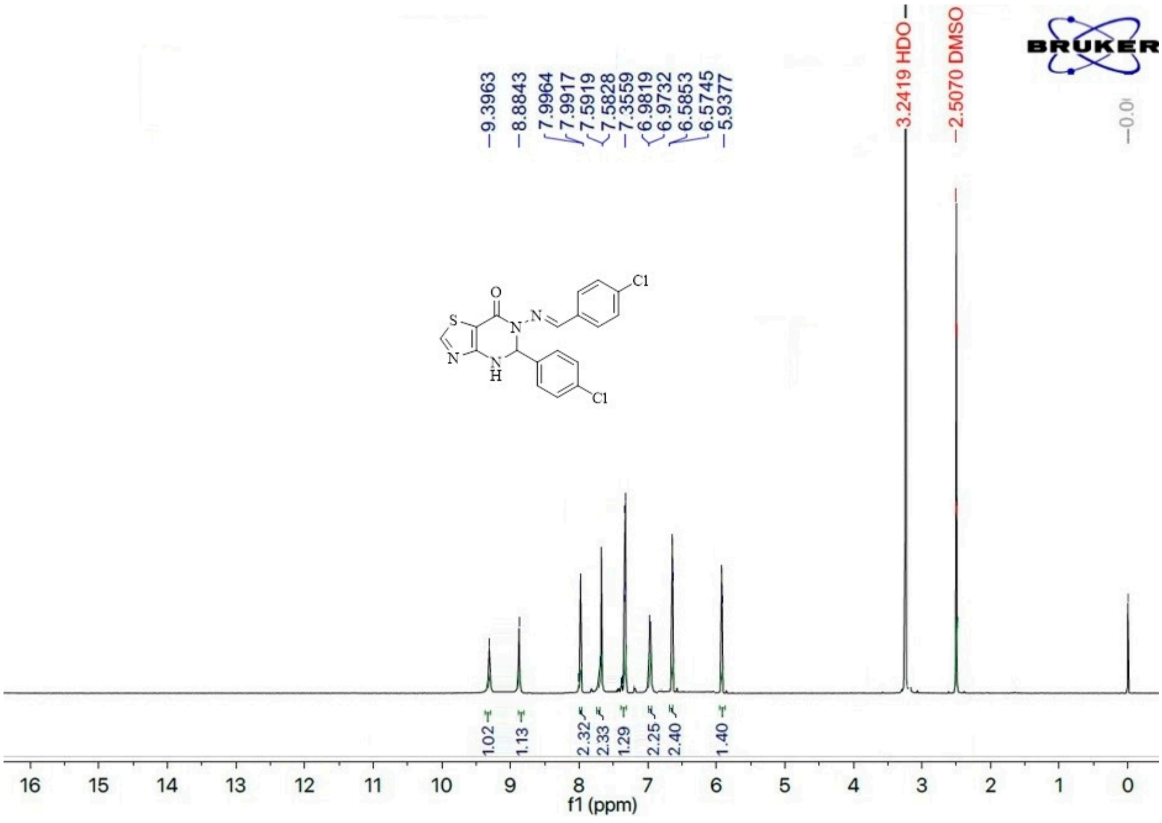


**Figure S16:** $^{1}$HNMR for compound-**3**

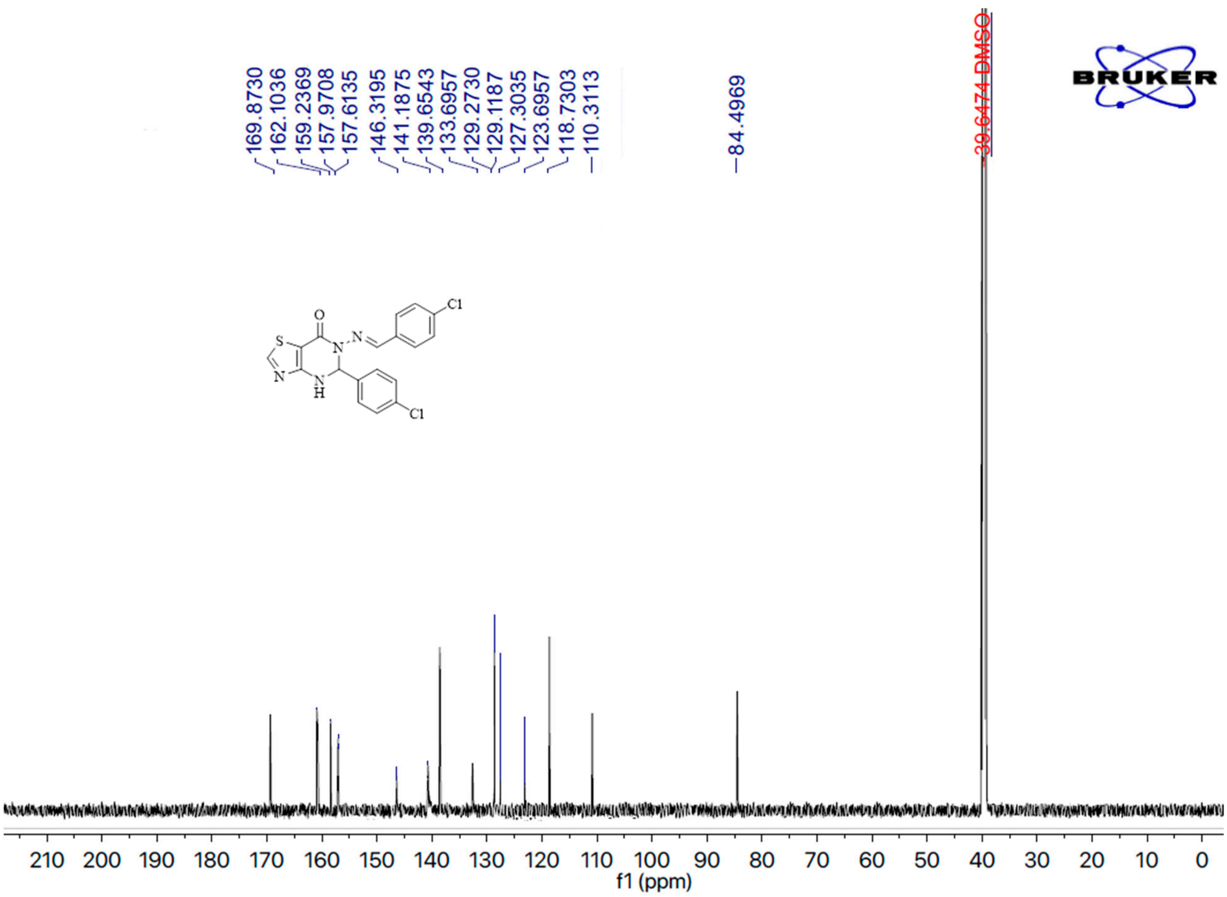


**Figure S17:** $^{13}$CNMR for compound-**3**

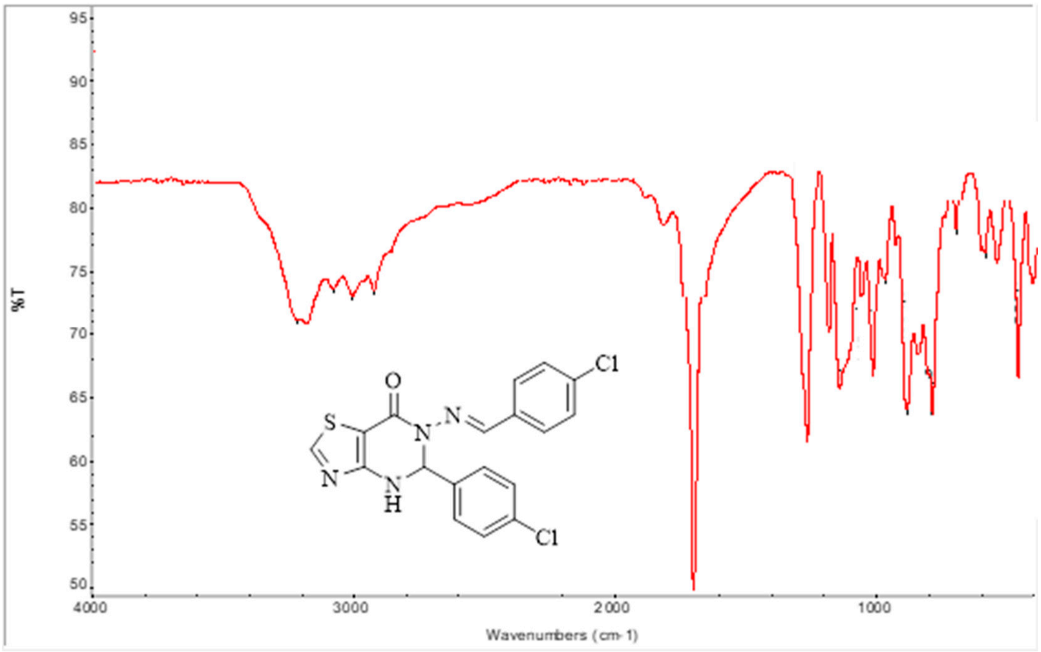


**Figure S18:** FTIR for compound-**3**

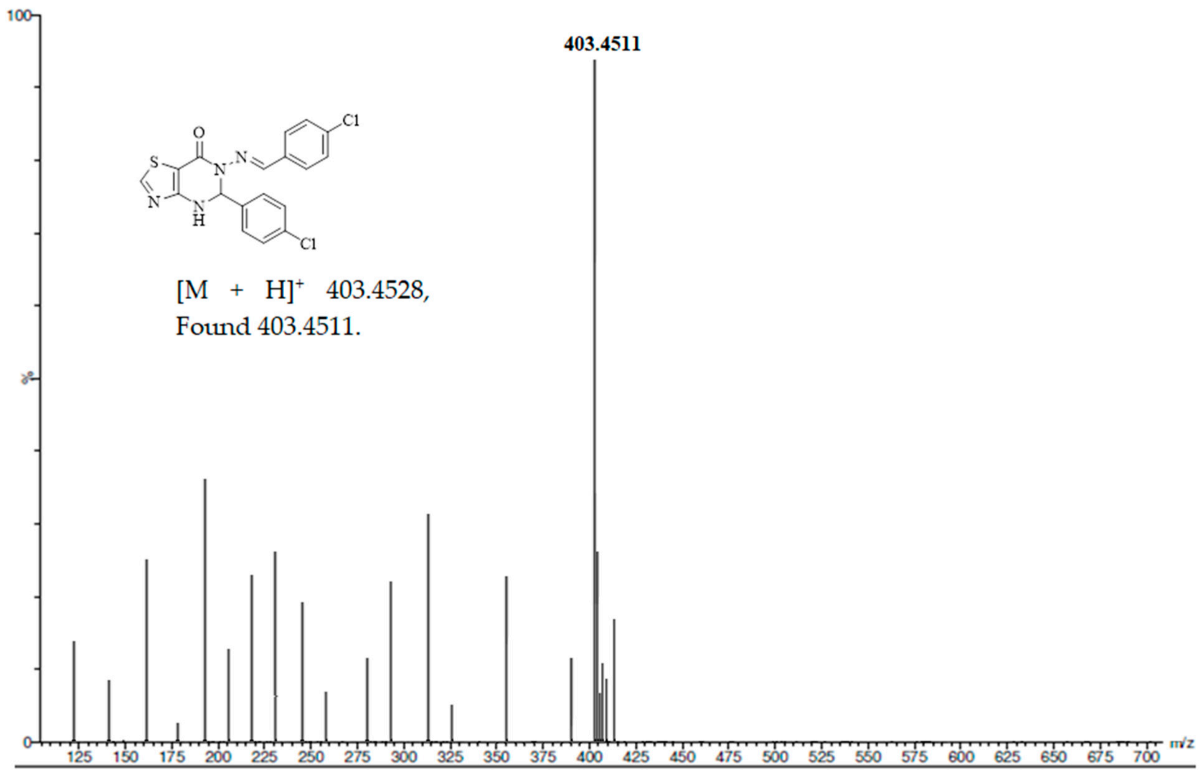


**Figure S19:** HRMS for compound-**3**

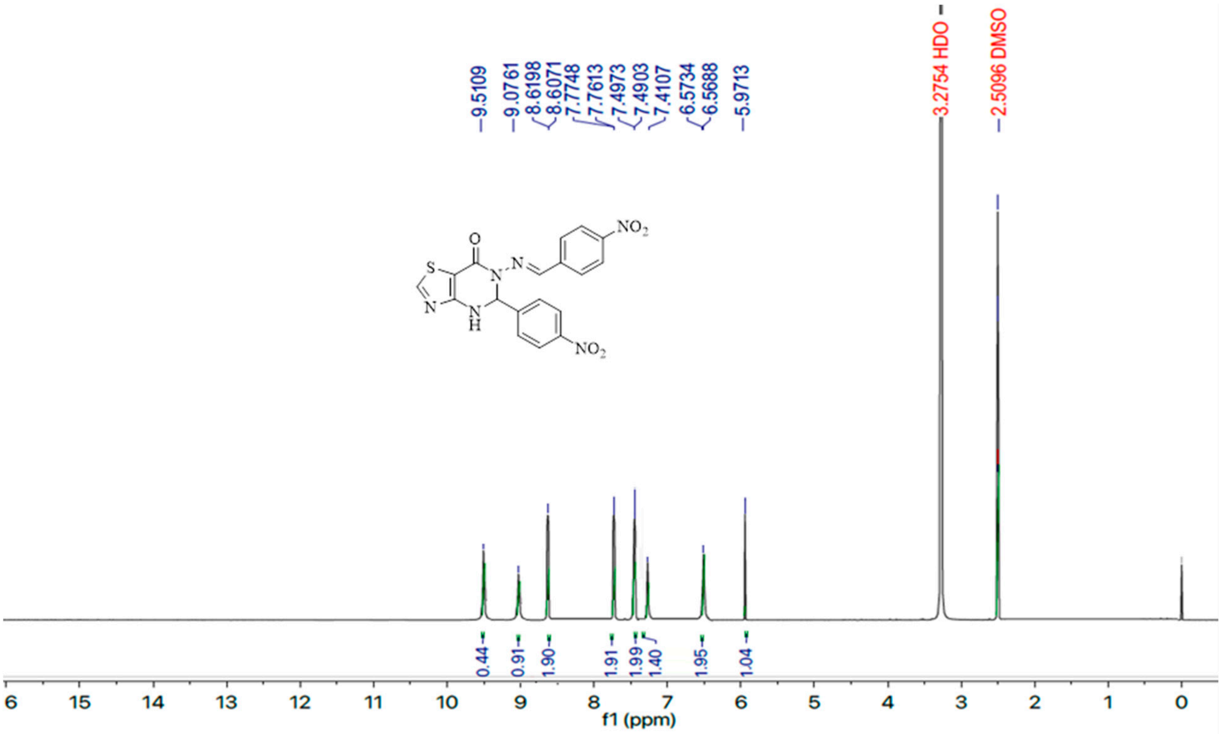


**Figure S20:** $^{1}$HNMR for compound-**6**

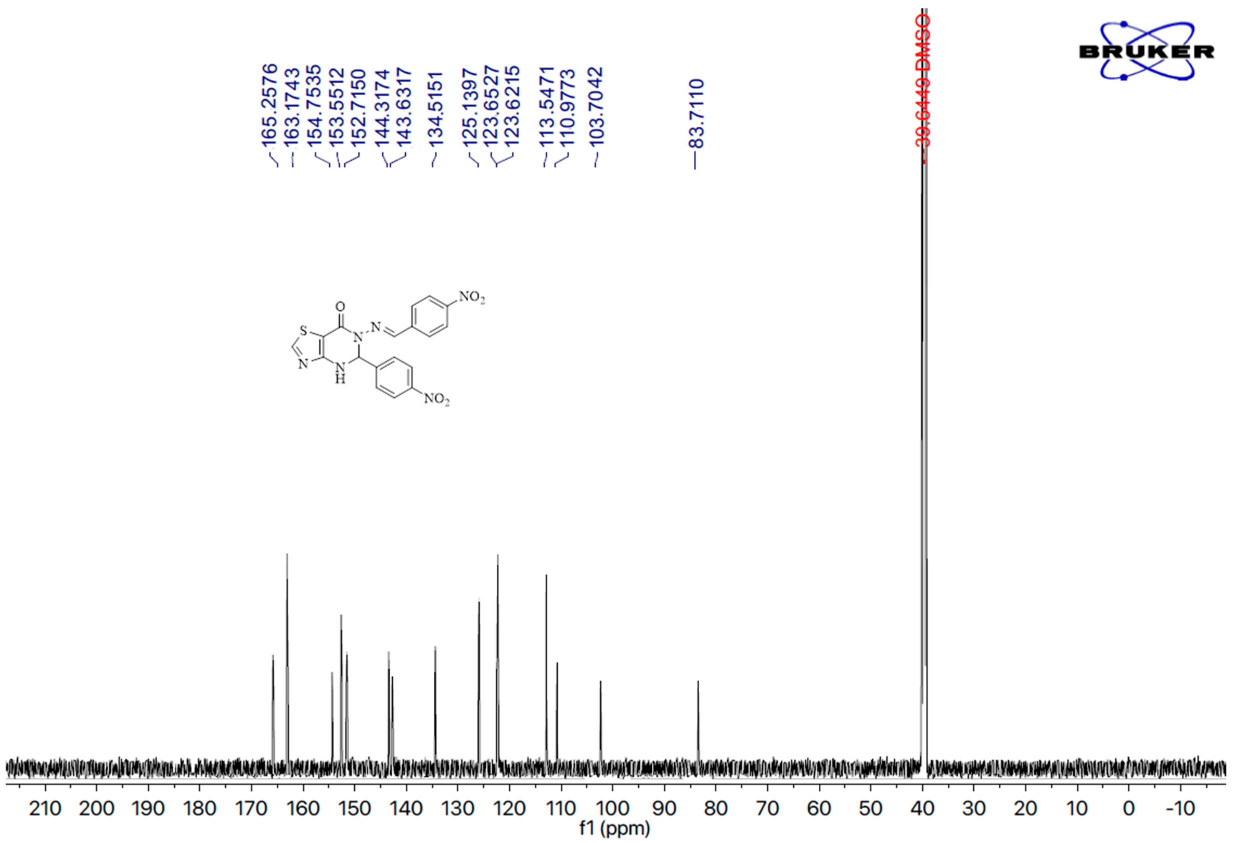


**Figure S21:** $^{13}$CNMR for compound-**6**

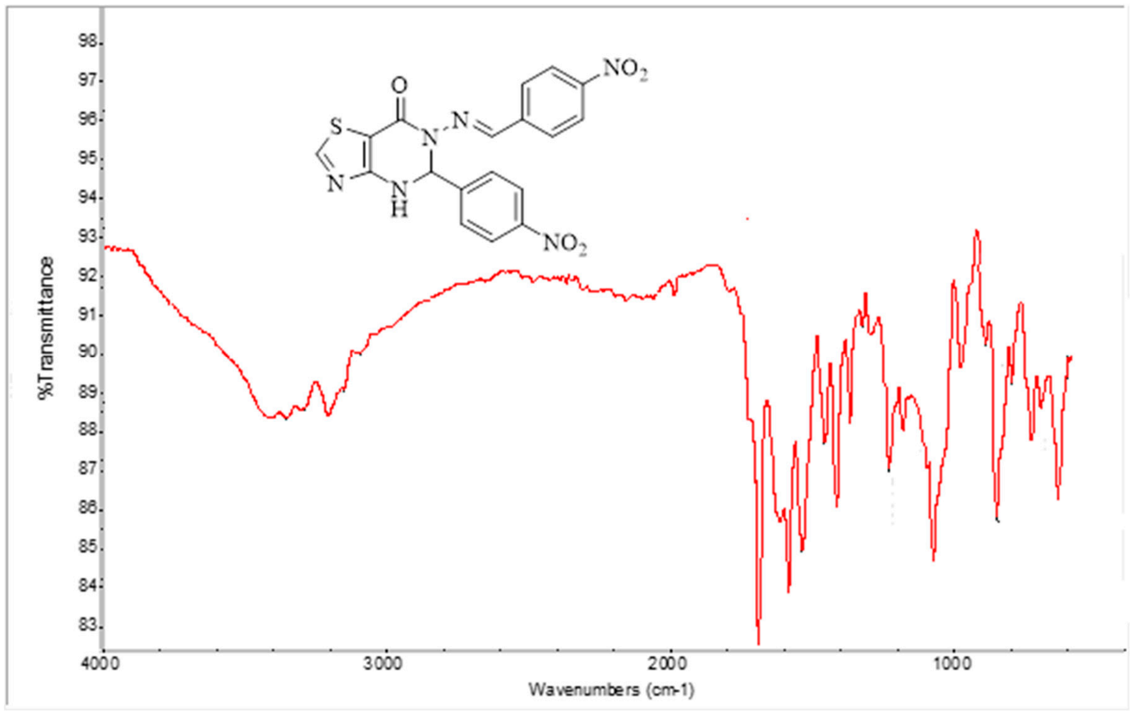


**Figure S22:** FTIR for compound-**6**

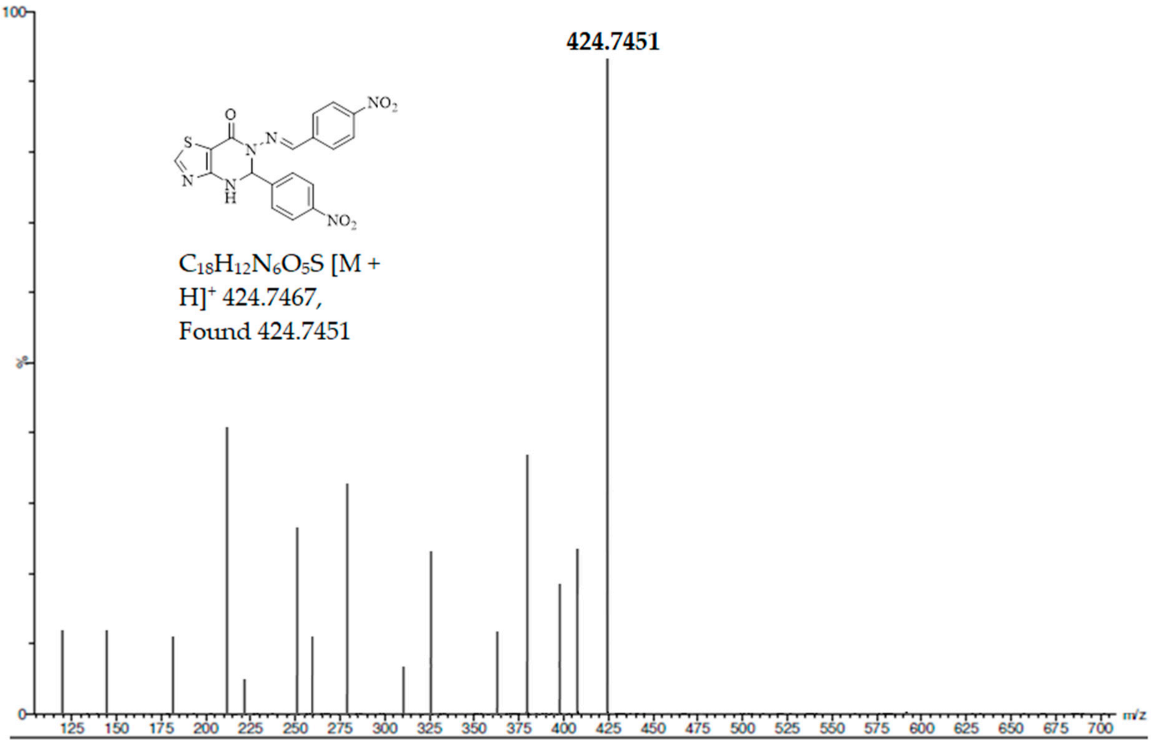


**Figure S23:** HRMS for compound-**6**

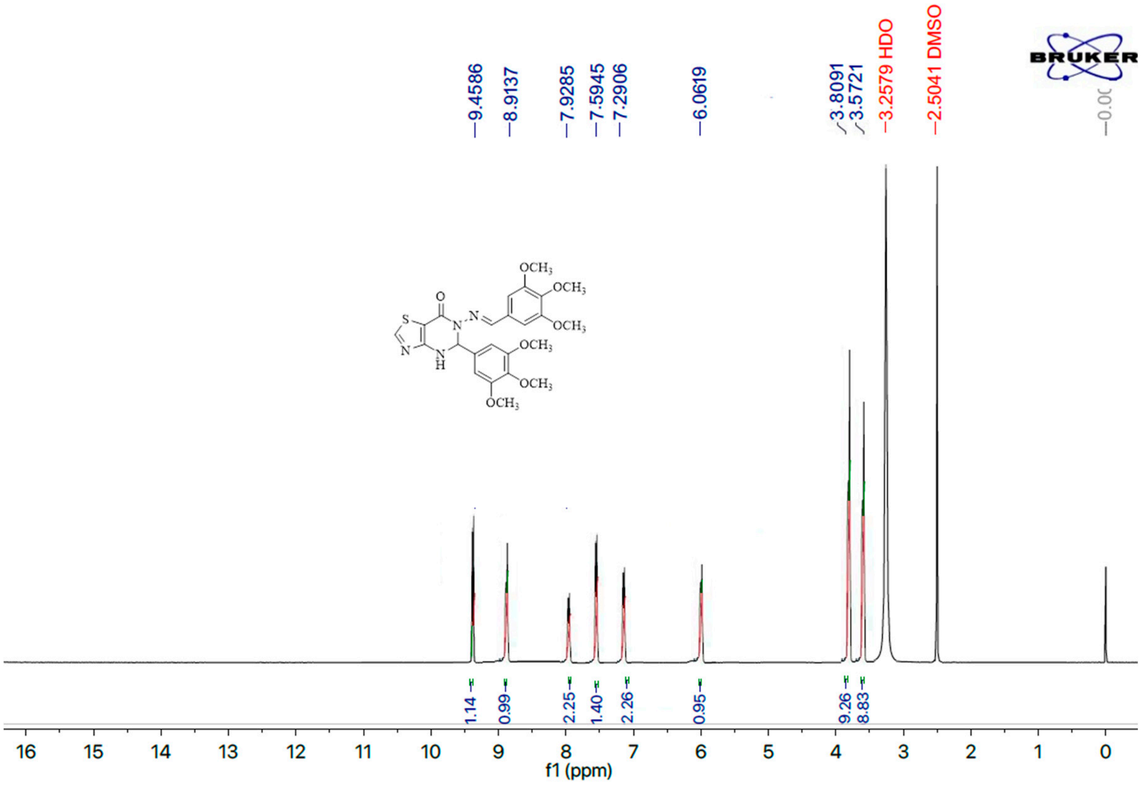


**Figure S24:** $^1$HNMR for compound-**10**

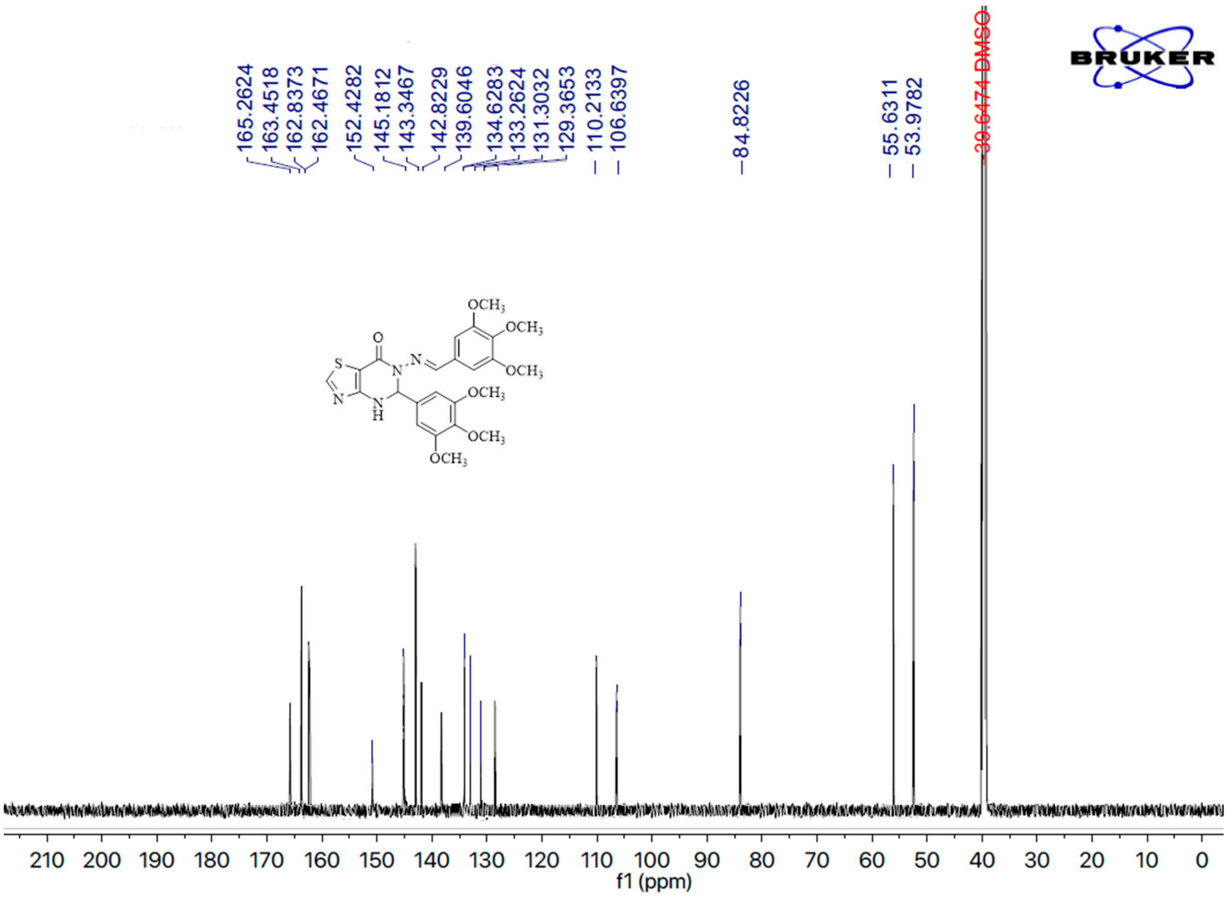


**Figure S25:** $^{13}$CNMR for compound-**10**

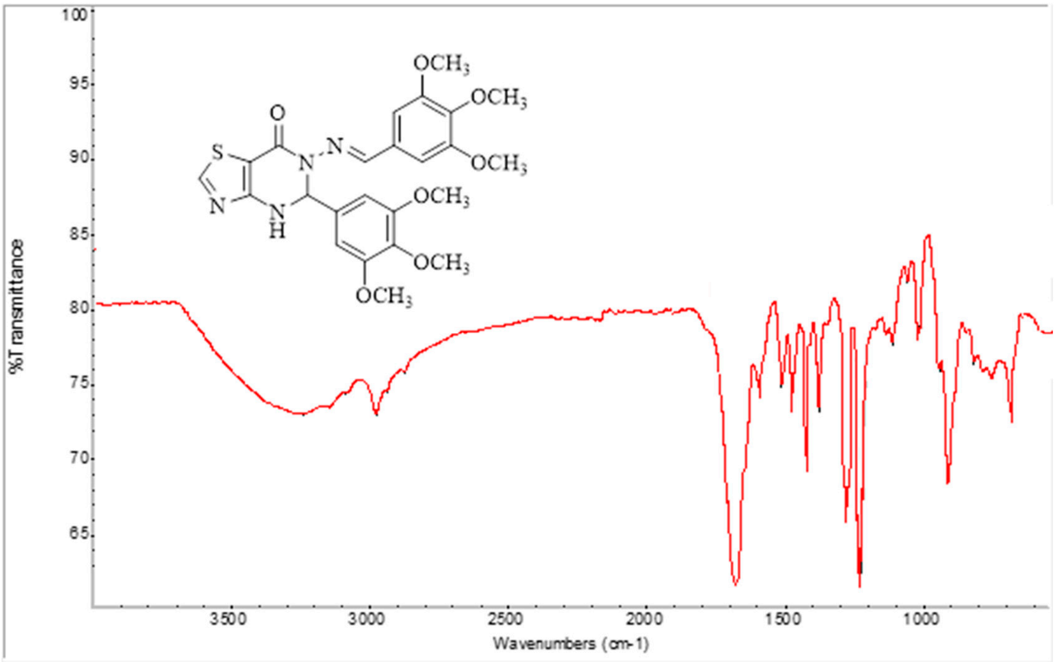


**Figure S26:** FTIR for compound-**10**

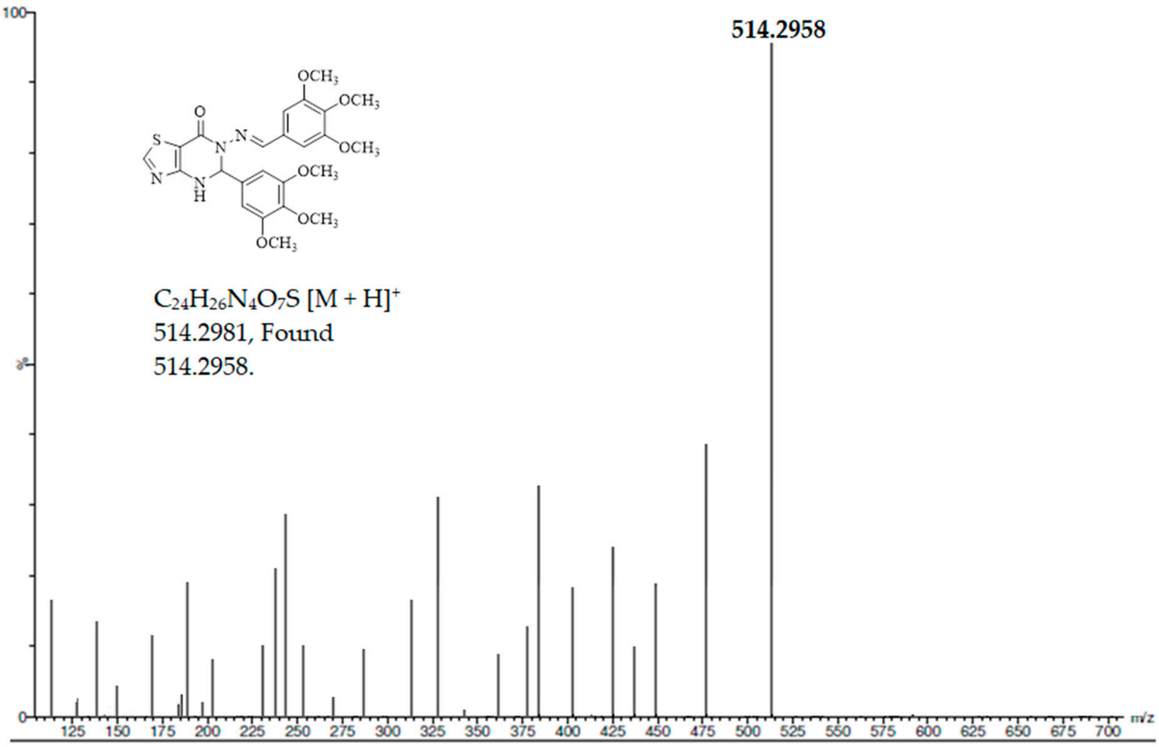


**Figure S27:** HRMS for compound-**10**

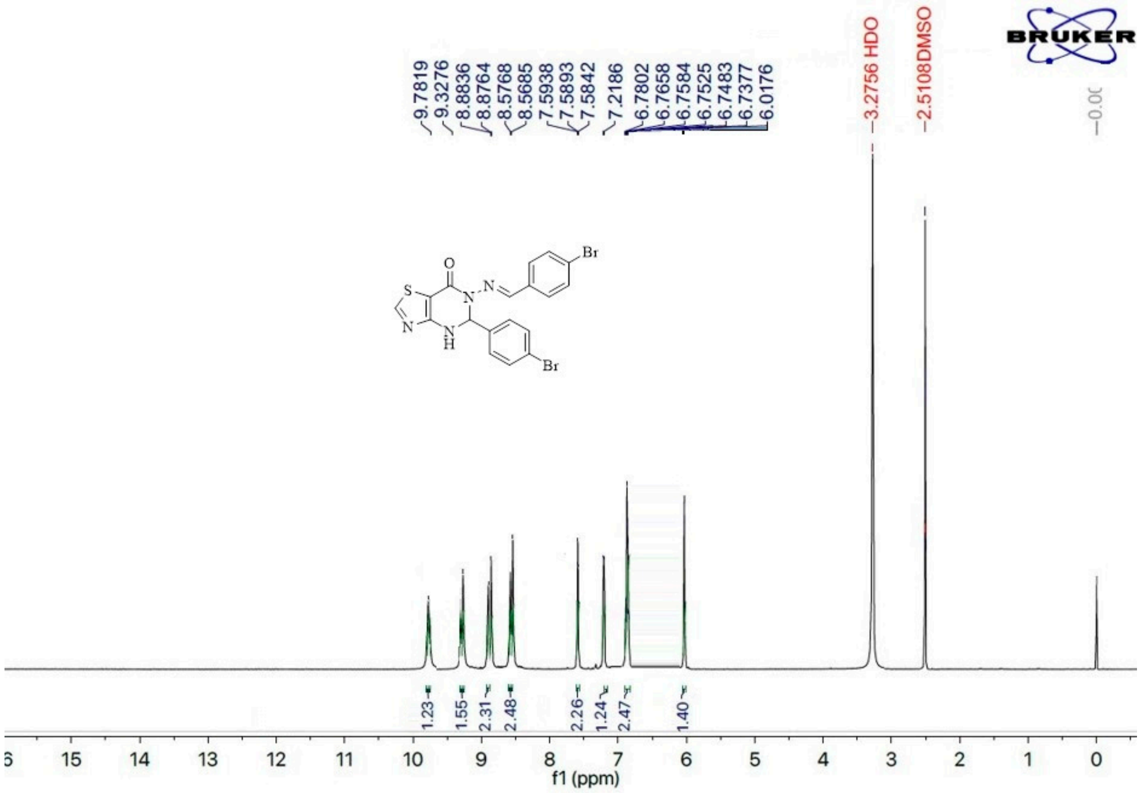


**Figure S28:** $^{1}$HNMR for compound-**12**

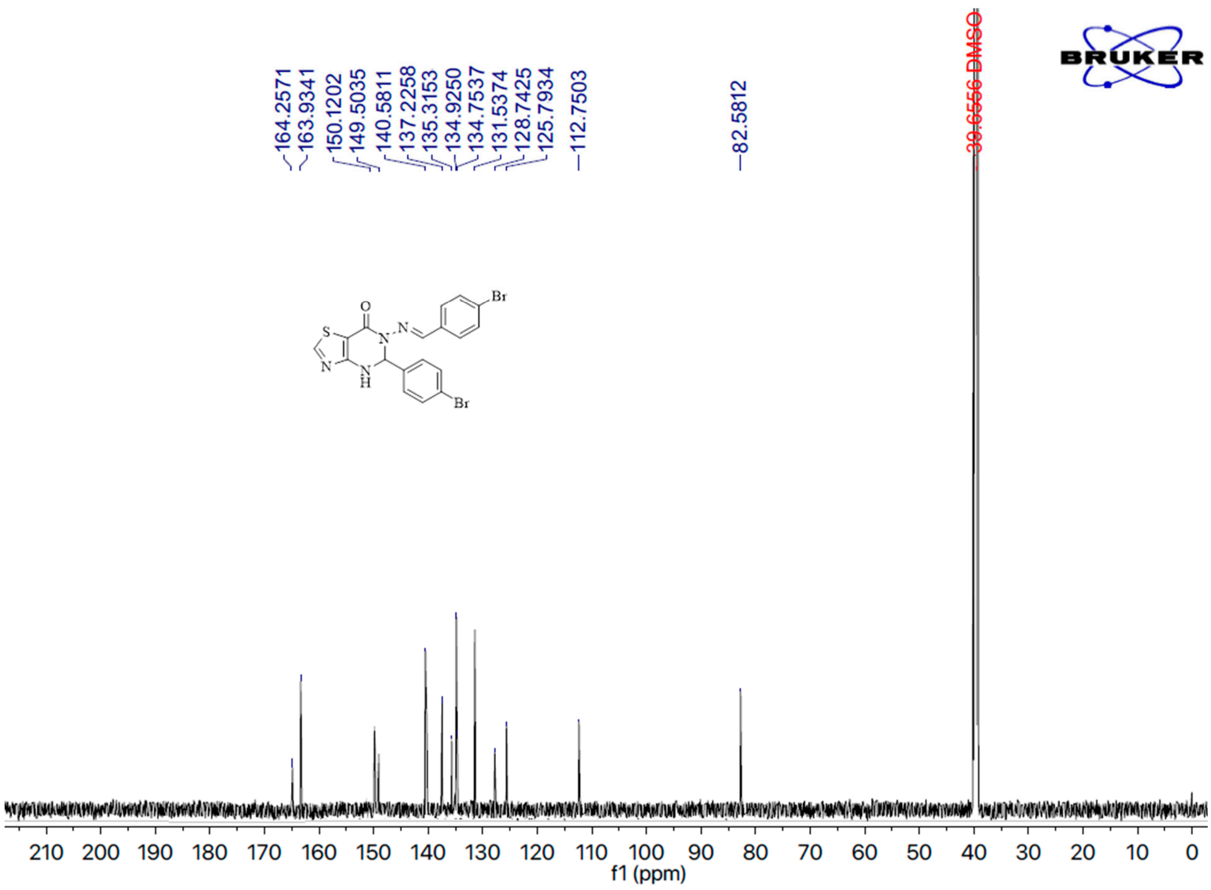


**Figure S29:** $^{13}$CNMR for compound-**12**

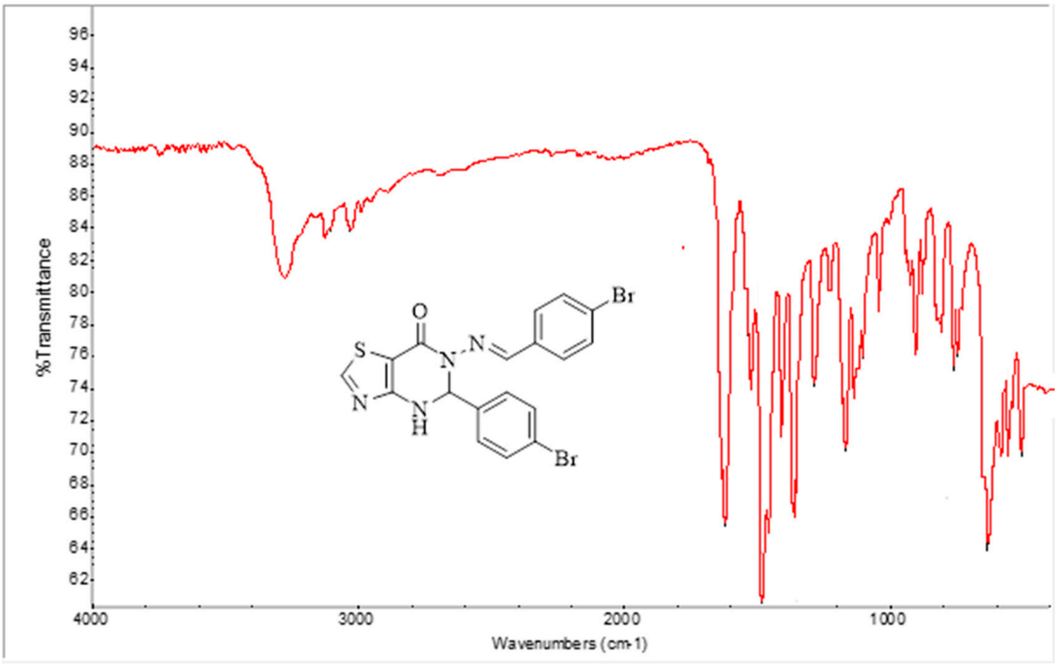


**Figure S30:** FTIR for compound-**12**

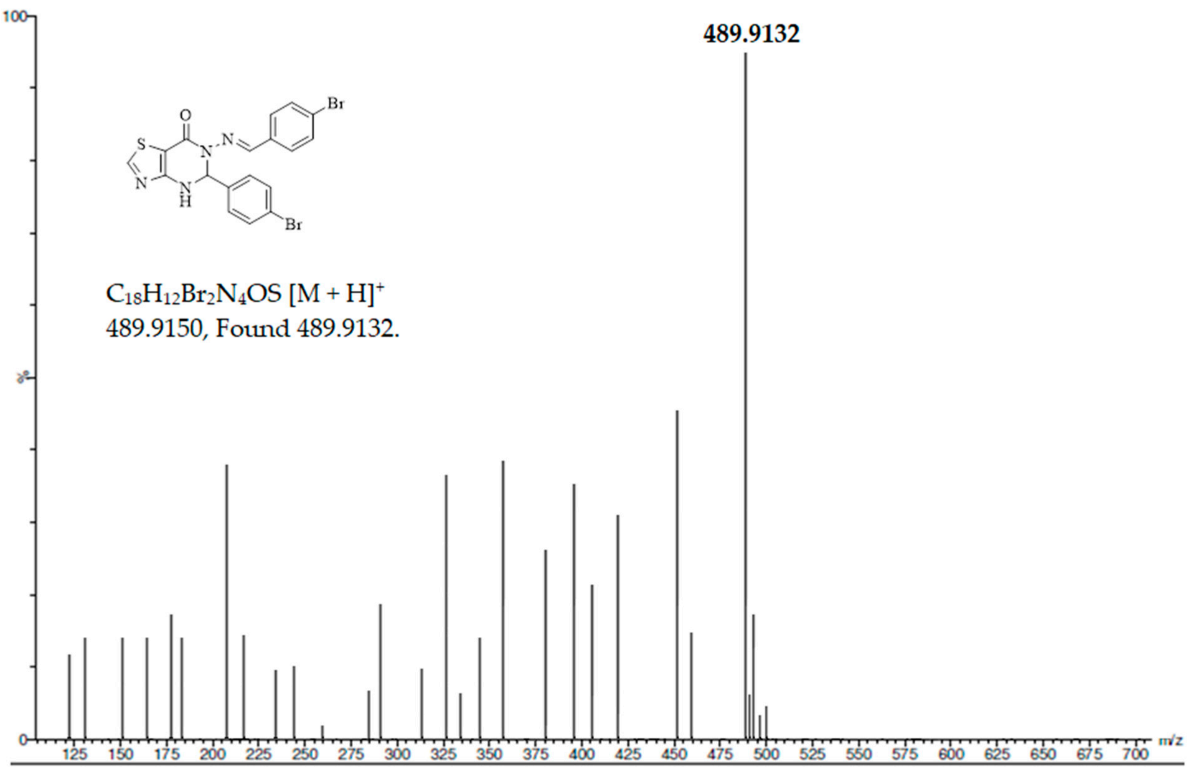


**Figure S31:** HRMS for compound-**12**

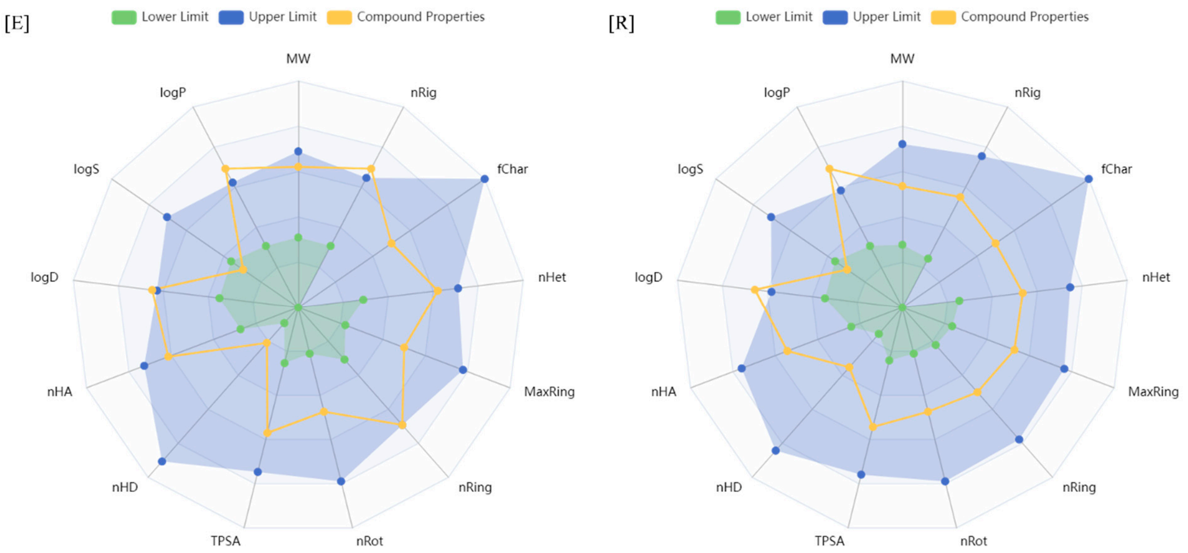


**Figure S32.** Bioavailability radar plots of novel compound-14 [E] and reference drug [R] showing physicochemical profiles. The evaluated physicochemical parameters are consistent with accepted oral drug-likeness requirements and support the suitable pharmacokinetic and bioavailability related profiles.

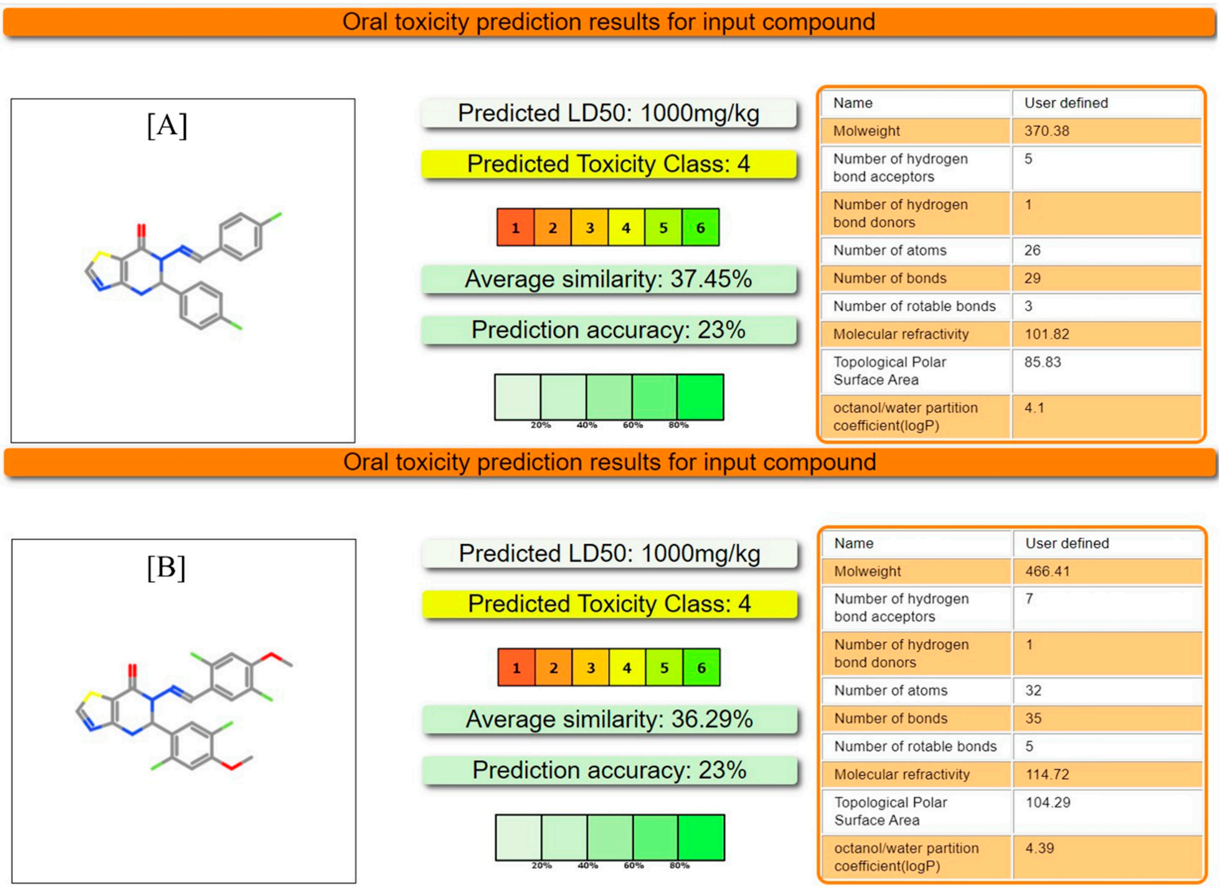


**Figure S33.** Computational prediction of oral toxicity profiles and toxicity class assignment for novel compound-2 [A] and compound-8 [B]. Toxicity assessment provides preliminary insights of the safety profile of the investigated candidates and their prospective suitability for further pharmacological applicability.

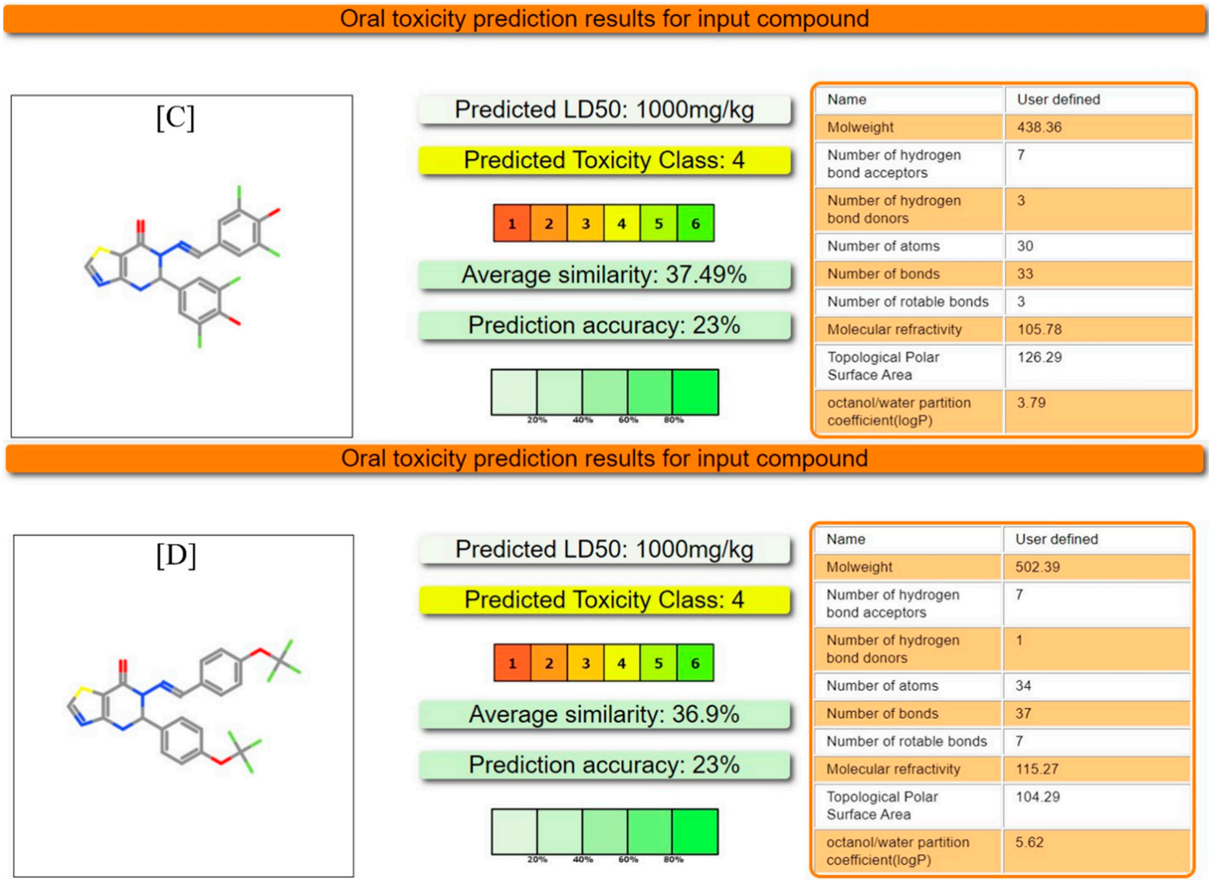


**Figure S34.** Computational prediction of oral toxicity profiles and toxicity class assignment for novel compound-9 [C] and compound-13 [D]. Toxicity assessment provides preliminary insights of the safety profile of the investigated candidates and their prospective suitability for further pharmacological applicability.

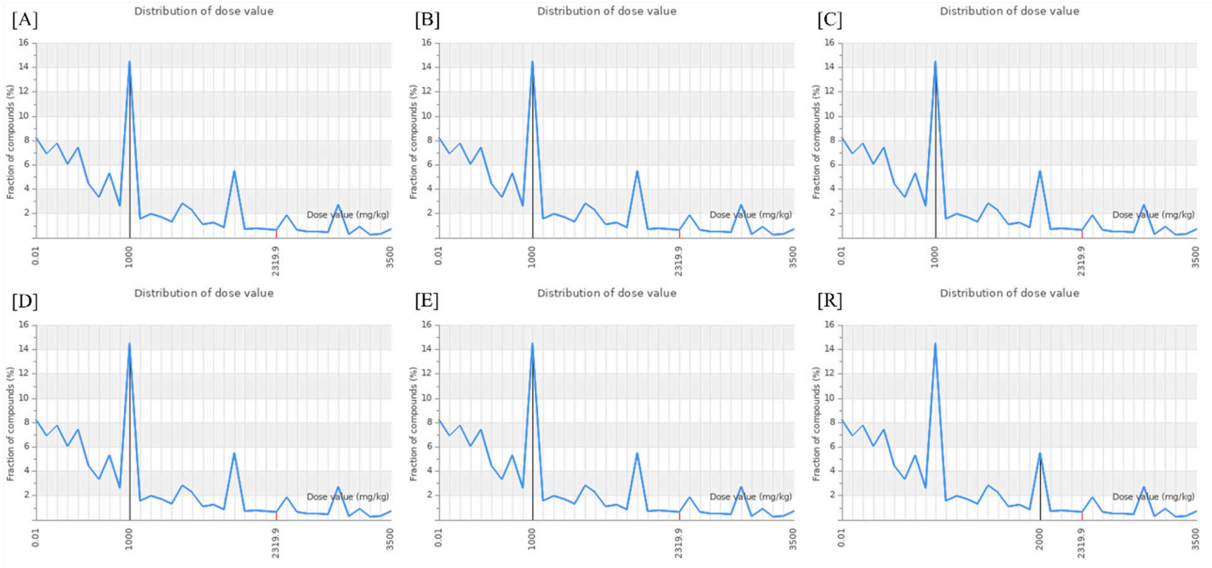


**Figure S35.** Dose-distribution profiles of novel compound-2 [A], compound-8 [B], compound-9 [C], compound-13 [D], compound-14 [E] and reference drug [R].

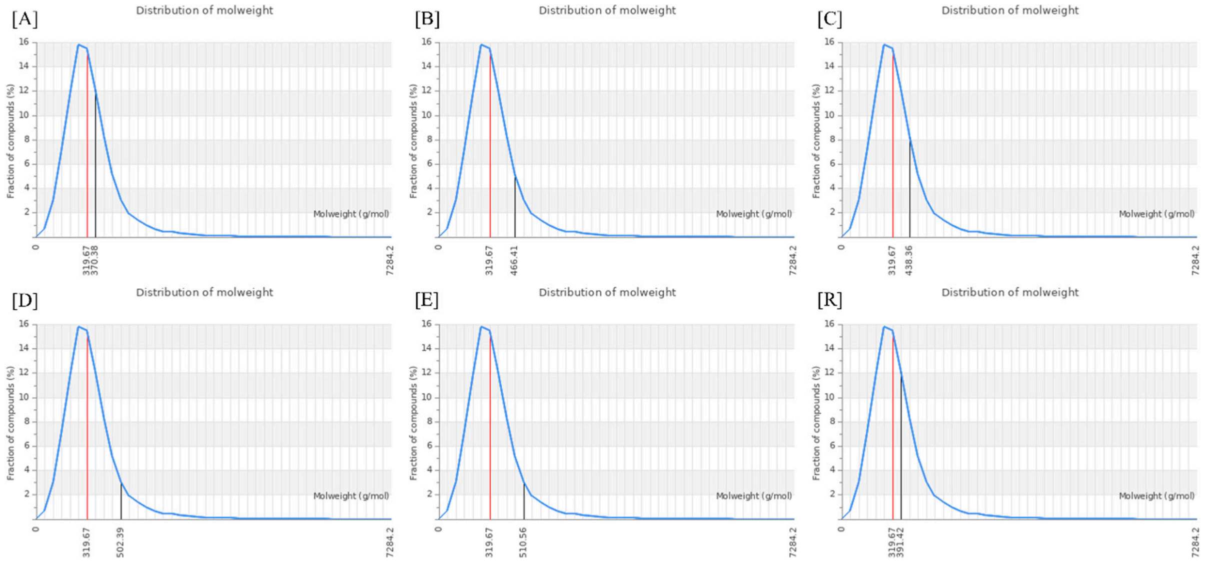


**Figure S36.** Mol-weight-distribution profiles of novel compound-2 [A], compound-8 [B], compound-9 [C], compound-13 [D], compound-14 [E] and reference drug [R].

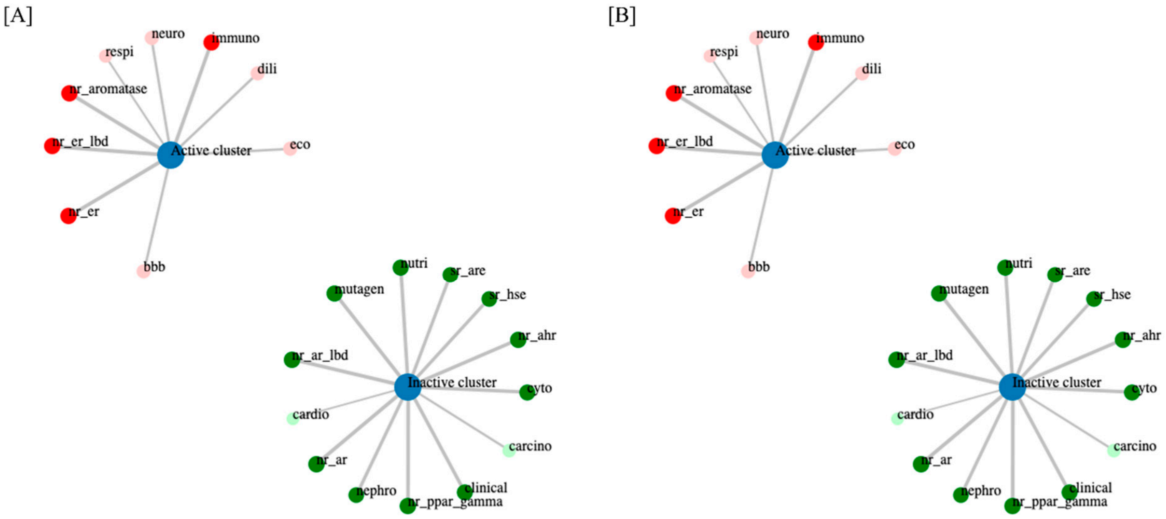


**Figure S37.** Bioavailability radar plot for the prediction of drug-likeness and pharmacokinetic profiles of novel compound-2 [A] and compound-8 [B].

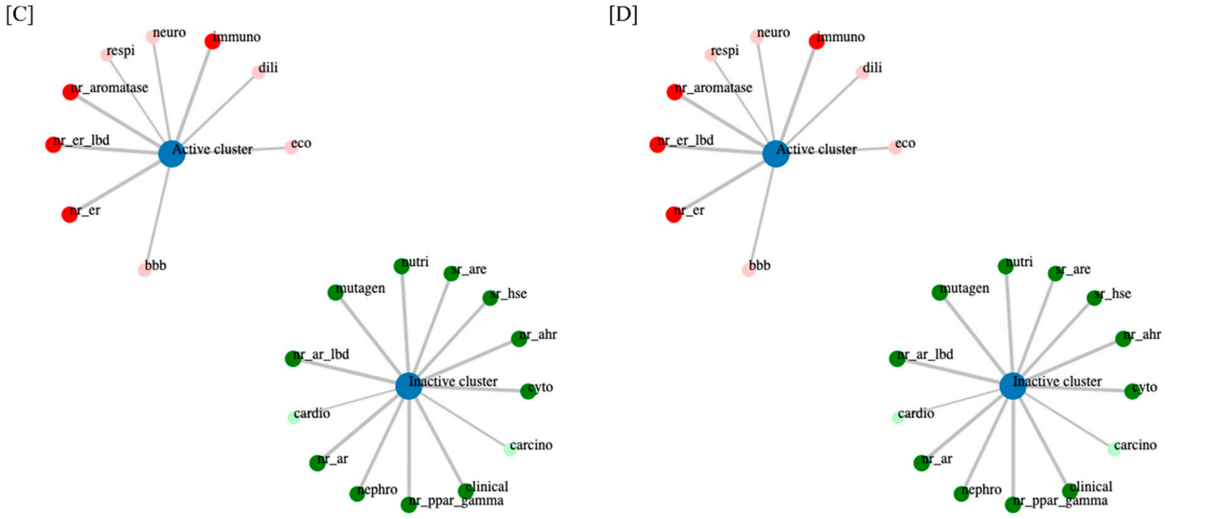


**Figure S38.** Bioavailability radar plot for the prediction of drug-likeness and pharmacokinetic profiles of novel compound-9 [C] and compound-13 [D].

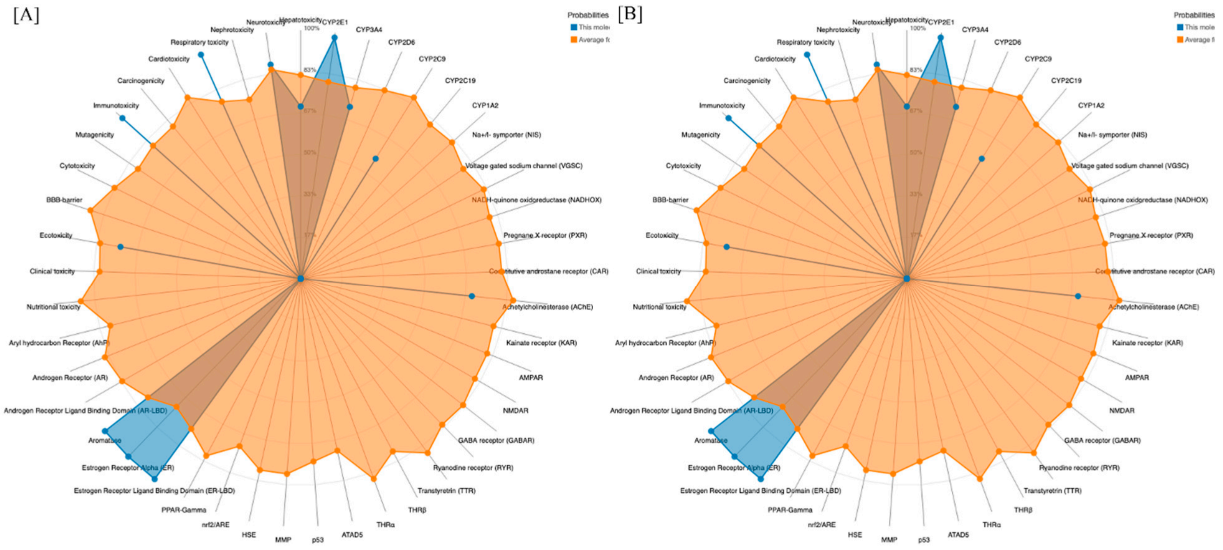


**Figure S39.** Network chart-based the profile prediction of novel compound-2 [A] and compound-8 [B].

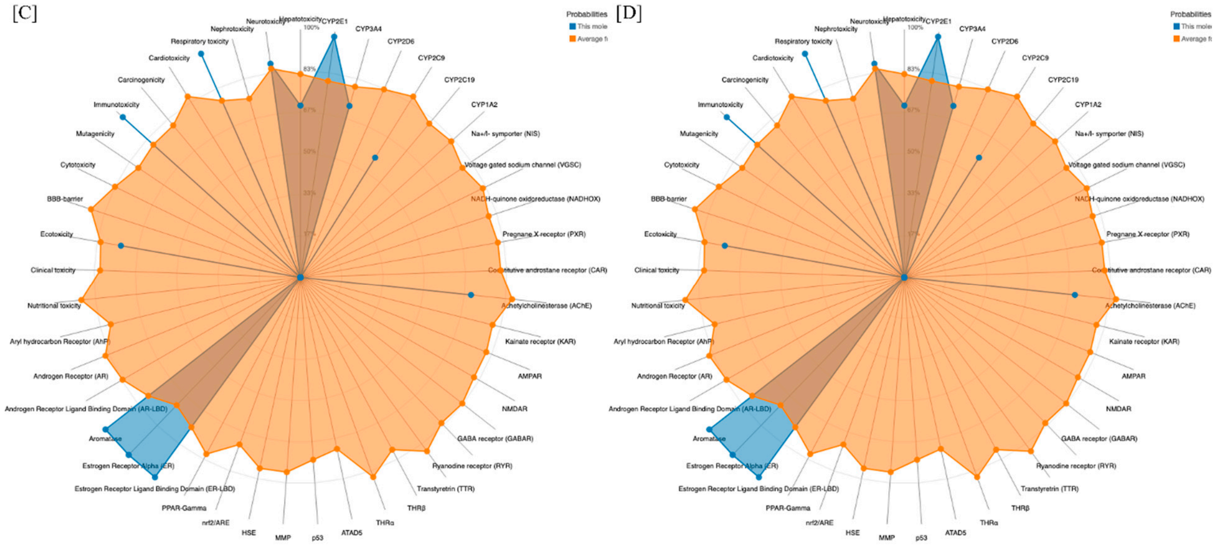


**Figure S40.** Network chart-based the profile prediction of novel compound-9 [C] and compound-13 [D].

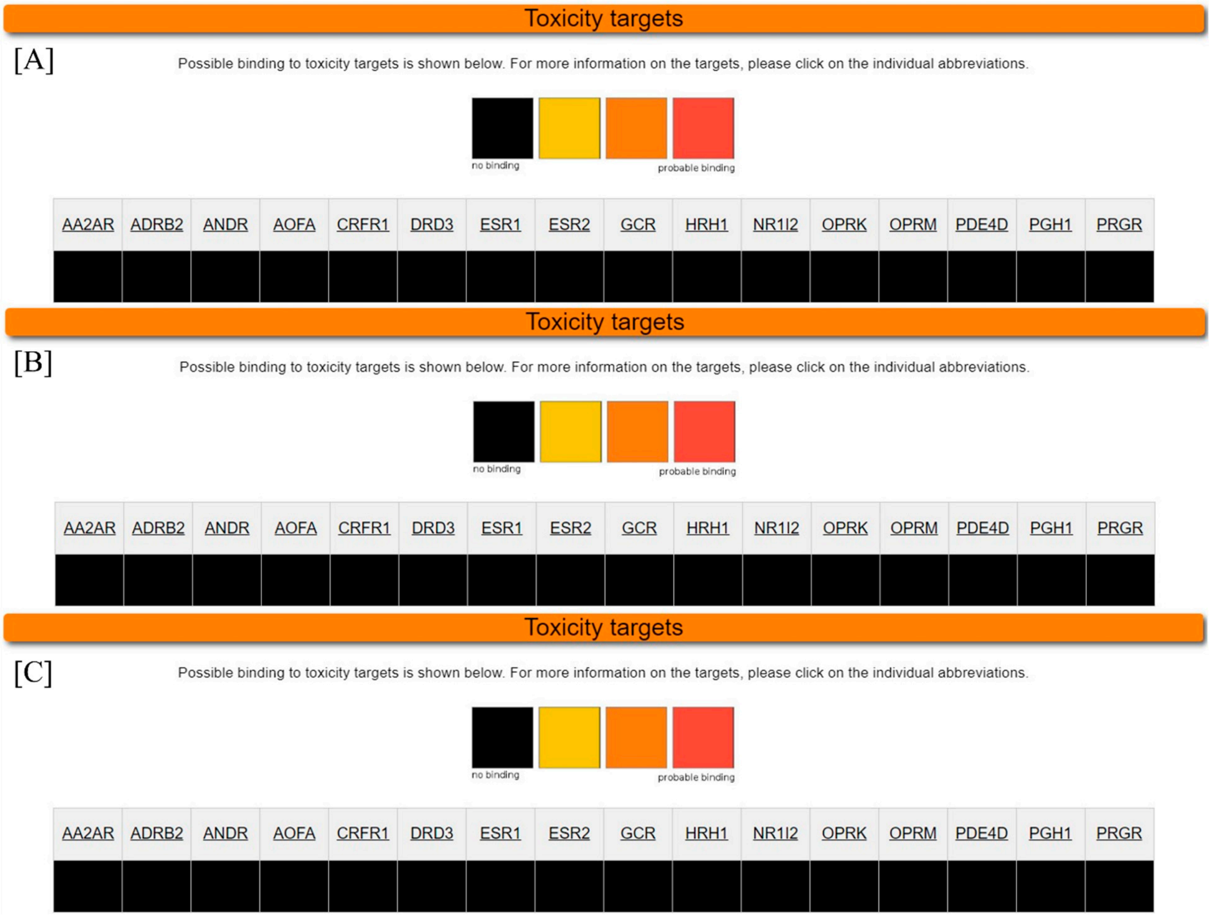


**Figure S41.** Toxicity targets prediction of novel compound-2 [A], compound-8 [B] and compound-9 [C].

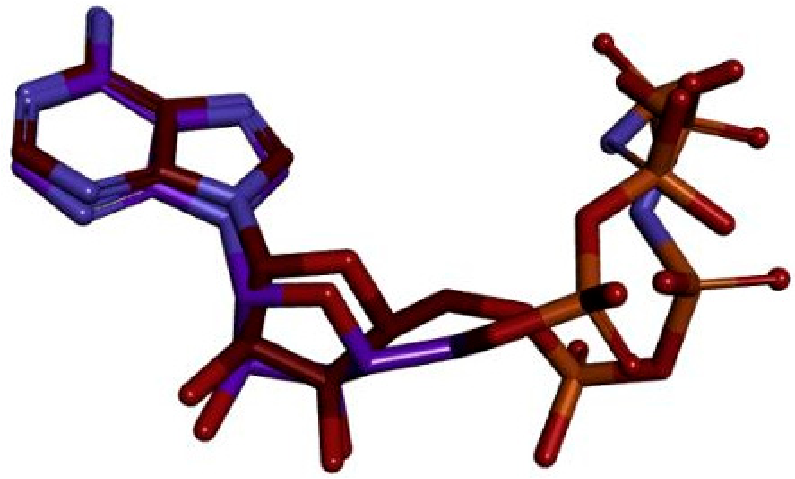

**Figure S42.**. Superimposition of the experimentally determined co-crystallized ligand (red) and its re-docked pose (purple) within the RSK4 kinase binding pocket. The close overlap between the two conformations validates the molecular docking protocol and demonstrates its reliability in accurately reproducing the experimentally observed binding orientation

## S1. Comparison of molecular docking binding energies with the experimental RSK4 inhibitory activities

A comparison of the molecular docking binding energies with the experimental RSK4 inhibitory activities revealed a generally favourable relationship between predicted binding affinity and biological potency. Compounds **2, 8, 9, 13,** and **14**, with $IC_{50}$ values 17.08 ± 1.65 nM, 18.08 ± 0.99 nM, 15.32 ± 1.35 nM, 17.89 ± 1.35 nM, and 21.45 ± 1.69 nM respectively, emerged as the most potent candidates as remarkable nanomolar RSK4 inhibitory activity that was comparable to that of standard drug BI-D1870 with $IC_{50}$ value 33.16 ± 1.34 nM. Among these, compound 8 showed the strongest predicted binding affinity (-10.2 kcal/mol), consistent with its high inhibitory activity. In contrast, compound 9 displayed the lowest experimental $IC_{50}$ (15.32 ± 1.35 nM) despite a comparatively less favourable docking score (-7.9 kcal/mol). This observation indicates that docking scores alone do not fully account for biological potency, as enzyme inhibition is also influenced by protein flexibility, solvent-mediated interactions, ligand dynamics, and kinetic parameters that are not completely represented in rigid docking calculations. Therefore, the docking analysis provides qualitative support for the experimental findings, while the overall inhibitory activity is governed by multiple structural and physicochemical factors as were shown in Table **S5.2**

It is important to emphasize that the molecular docking and molecular dynamics simulations presented herein provide computational insights into the potential binding modes and interaction stability of the synthesized compounds with RSK4, but they do not constitute experimental evidence of

biological activity, potency, or selectivity. Likewise, the ADMET and toxicity results represent in-silico predictions and should be regarded as preliminary indicators rather than experimentally validated pharmacological or safety profiles. These computational findings are therefore intended to complement the experimental biological results and to provide a rationale for prioritizing promising compounds for further investigation. Definitive assessment of RSK4 potency and selectivity, cellular activity, mechanism of action, and in-vivo efficacy will require appropriate experimental validation.

**Table S5**: Correlate the experimental RSK4 inhibition ($IC_{50}$) with the molecular docking binding energies

| **Compound** | **Docking Score (kcal/mol)** | **RSK4 $IC_{50}$ (nM)** |
|---|---|---|
| **2** | -9.5 | 17.08 ± 1.65 |
| **8** | -10.2 | 18.08 ± 0.99 |
| **9** | -7.9 | 15.32 ± 1.35 |
| **13** | -9.0 | 17.89 ± 1.35 |
| **14** | -10.0 | 21.45 ± 1.69 |
| **Standard BI-D1870** | -9.8 | 33.16 ± 1.34 nM |
